# Systematic analysis reveals novel insight into the molecular determinants of function, diversity and evolution of sweet taste receptors T1R2/T1R3 in primates

**DOI:** 10.3389/fmolb.2023.1037966

**Published:** 2023-01-25

**Authors:** Congrui Wang, Yi Liu, Meng Cui, Bo Liu

**Affiliations:** ^1^ School of Food Science and Engineering, Qilu University of Technology (Shandong Academy of Sciences), Jinan, China; ^2^ Department of Pharmaceutical Sciences, Northeastern University, Boston, MA, United States

**Keywords:** primate, sweet taste receptor, taxonomic distribution, coevolution, epistasis, species-dependent sweet taste, molecular determinants

## Abstract

Sweet taste is a primary sensation for the preference and adaption of primates to diet, which is crucial for their survival and fitness. It is clear now that the sweet perception is mediated by a G protein-coupled receptor (GPCR)-sweet taste receptor T1R2/T1R3, and many behavioral or physiological experiments have described the diverse sweet taste sensitivities in primates. However, the structure-function relationship of T1R2s/T1R3s in primates, especially the molecular basis for their species-dependent sweet taste, has not been well understood until now. In this study, we performed a comprehensive sequence, structural and functional analysis of sweet taste receptors in primates to elucidate the molecular determinants mediating their species-dependent sweet taste recognition. Our results reveal distinct taxonomic distribution and significant characteristics (interaction, coevolution and epistasis) of specific key function-related residues, which could partly account for the previously reported behavioral results of taste perception in primates. Moreover, the prosimians Lemuriformes species, which were reported to have no sensitivity to aspartame, could be proposed to be aspartame tasters based on the present analysis. Collectively, our study provides new insights and promotes a better understanding for the diversity, function and evolution of sweet taste receptors in primates.

## Introduction

The sweet taste sensation is one of the five primary taste qualities (sweet, bitter, umami, sour and salty) enabling animals to distinguish beneficial foods and facilitating their adaption to environmental niches (Lindemann, 1996; Kim et al., 2004). Diversification of sweet taste preferences in primate species toward various sweeteners has been extensively reported, and the sweet sensory system of primates shows remarkable flexibility that essentially increases their survival capabilities (Jordan et al., 2015). It is well known that the sweet taste is mediated by a G protein-coupled receptor (GPCR)-sweet taste receptor located on the membrane of oral gustatory buds (Jordan et al., 2009). Therefore, information of the structure and function of this receptor in primates is meaningful for understanding the molecular basis of their species-dependent sweet taste as well as evolution.

**FIGURE 1:**
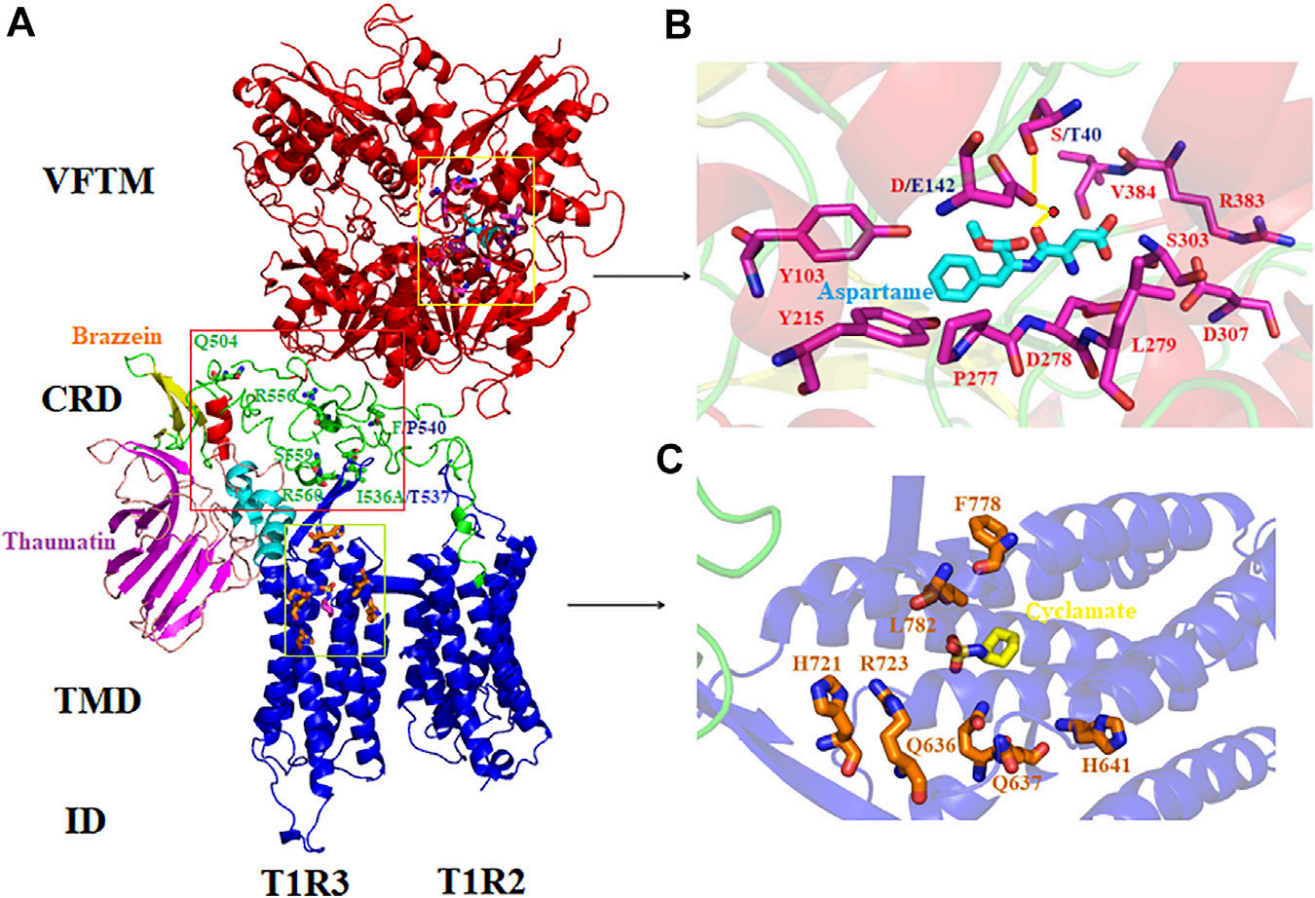
Molecular simulations and schematic representation of the human sweet taste receptor. **(A)** Modeling of the human T1R2/T1R3 and its binding sites for different sweeteners. The conserved VFTM, CRD and TMD/ID region are colored in red, green and blue, respectively. The key residues involved in the species-dependent sweeteners (thaumatin and brazzein) recognition are labeled and shown as stick model. Residues appeared in other primates that mediate species-dependent sweet taste are colored in blue. **(B)** Binding site of aspartame in the sweet taste receptor. The receptor residues are shown in stick model and the water molecule is shown as red circle. Hydrogen bonds are shown as yellow solid lines. **(C)** Binding site of cyclamate in the sweet taste receptor. The residues in the pocket involved in cyclamate binding are according to the previous result (Jiang et al., 2005b). The figures were generated with the PyMOL software.

The sweet taste receptor is a heterodimer composed of two subunits T1R2 and T1R3, which belongs to the family C GPCRs (Nelson et al., 2001; Li et al., 2002; Zhao et al., 2003). This receptor is characterized by a large extracellular domain, which consists of an N-terminal Venus flytrap module (VFTM) and a cysteine-rich domain (CRD), a heptahelical transmembrane domain (TMD) and an intracellular domain (ID) (Figure 1A) (Pin et al., 2003; Xu et al., 2004). With the methods of molecular simulations and functional mutagenesis/chimera analysis, previous studies have revealed that there are multiple binding sites in the receptor for various sweeteners (Jiang et al., 2005a; b; Nie et al., 2005; Cui et al., 2006; Winnig et al., 2007; Liu et al., 2011; Masuda et al., 2012). However, the spatial structure information of sweet taste receptor and its complexes with sweeteners is still unavailable due to some experimental obstacles.

It has been demonstrated that the basic and simple sugars or amino acids can be perceived by all of the primates found so far, as evidenced in many previous behavioral or electrophysiological tests (Hellekant et al., 1980; Nofre et al., 1996). However, the artificial sweetener aspartame can be perceived by Old World monkeys, great apes, gibbons and humans, but not by New World monkeys and rodents, as proved by both *in vivo* and vitro investigations (Glaser et al., 1995; Liu et al., 2011). With molecular simulations and functional mutagenesis analysis, previous studies have characterized the binding site of aspartame and its involved residues located in the extracellular VFTM region of human T1R2: S40, Y103, D142, Y215, P277, D278, L279, S303, D307, R383 and V384 in human T1R2. Furthermore, we have revealed that two critical residues S40 and D142 in human T1R2, which correspond to T40 and E142 in squirrel monkey T1R2 respectively, mediate the species-dependent taste toward aspartame between human (taster) and squirrel monkey (non-taster) (Liu et al., 2011). Moreover, residue at site 142 is critical that determines the switch between taste and non-taste toward aspartame *via* interacting with aspartame *via* a water bridge, while residue at site 40 modulates the intensity of taste and is suggested to mediate the signal transduction and conformation change upon receptor activation (Maillet et al., 2015).

Another intriguing species-dependent taste is toward the intensively sweet proteins. Previous studies have shown that the sweet-tasting proteins could be perceived by Old World monkeys, apes and humans, but not by New World monkeys and rodents (Glaser et al., 1978; Glaser et al., 1998). It is still unclear nowadays how these large weight and bulk sweet-tasting proteins bind and interact with the sweet taste receptor. With functional mutagenesis analysis, Jiang et al. reported that the discrepancy of residues A537 and F540 located in the CRD of human T1R3, which correspond to T542 and P545 of mouse T1R3 respectively, jointly determine the different sensitivity between human (taster) and mouse (non-taster) toward the sweet-tasting protein brazzein (Jiang et al., 2004). Furthermore, It was revealed that five residues (Q504, A537, R556, S559 and R560) located in the CRD of human T1R3 play important roles for the sensitivity to the sweet-tasting protein thaumatin (Masuda et al., 2013). Therefore, it seems that the CRD region in T1R3 mediate the species-dependent recognition toward sweet-tasting proteins. In Jiang et al.’s result, the mouse to human mutations T542A could gain the sensitivity to brazzein whereas P545F could not, indicating a more critical role of T542 (corresponding to A537 in human T1R3) (Jiang et al., 2004).

Analysis of the genomic and proteomic information available indicates that the sweet taste receptor T1R2/T1R3 is widely distributed in all kingdoms of mammalia including primates (Hellekant and Danilova, 1996; Li et al., 2009). To date, the T1R2s/T1R3s of primate species humans, squirrel monkeys and asian colobine monkeys have been characterized with the cell-based signal assays, indicating their functional differentiation (Liu et al., 2011; Nishi et al., 2018). Furthermore, many behavioral and gustatory responses tests have shown diverse sweet taste preferences in primates, suggesting a high degree of functional flexibility (Hellekant et al., 1981; Dieter et al., 1992; Hellekant et al., 1993; Hellekant and Danilova, 1996; Danilova et al., 2002). However, integrative analysis and comparison of the sequence, structure and function of T1R2s/T1R3s in primates as well as their determinative residues is still scarce. In this study, we performed a sequence and structure based analysis of the sweet taste receptors in primates to account for the experimental findings of their sweet taste, especially the key amino acids mediating their species-specific sweet taste recognition. Our findings provide new insight and deeper understanding into the molecular determinants of the diversity, function and evolution of sweet taste in primates.

## Materials and methods

### Collection of the data resources

The protein sequences of the sweet taste receptors T1R2 and T1R3 in primates were initially retrieved from the InterPro database (http://www.ebi.ac.uk/interpro) (Mitchell et al., 2015). Redundant and fragment sequences were removed manually. Additional sequences were obtained according to the published literature (Cai et al., 2006; Li et al., 2011). For the T1R2 or T1R3 sequences that are absent in the InterPro database, we searched the National Center for Biotechnology Information (NCBI) database (https://www.ncbi.nlm.nih.gov/). Furthermore, the redundant N-terminal sequences of T1R3s in sooty mangabey and crab-eating macaque were removed manually due to their identities of immature precursor/isoform. Moreover, the T1R3 sequence of black crested mangabey (Genbank accession No: KJ794728) was excluded for analysis because it is annotated as a pseudogene. To ensure the accurate prediction of the T1R2 and T1R3 gene families, the obtained putative protein sequences were screened for the presence of nine conserved cysteine residues in their CRD region using the clustalW sequence alignment tool (Thompson et al., 1994), and for the presence of seven transmembrane domains using TMHMM Server v.2.0 (https://services.healthtech.dtu.dk/service.php?TMHMM-2.0) (Krogh et al., 2001), which are characteristics of the taste receptors in class C GPCRs. The proteins identified in the databases are listed in Supplementary Data Sheet S1.

### Phylogenetic analysis and sequence similarity networks

The phylogenetic trees of T1R2 and T1R3 were generated using the MEGA 4.0 program. The trees are constructed using the method of neighbor-joining (NJ) and bootstrapping with 1,000 replications (Saitou and Nei, 1987). Construction of the sequence similarity networks (SSNs) based on the analyzed protein sequences was carried out with the Enzyme Function Initiative-Enzyme Similarity Tool (EFI-EST, https://efi.igb.illinois.edu/efi-est/), and the results were visualized with the Cytoscape 3.9 software (Shannon et al., 2003; Gerlt et al., 2015).

### Multiple sequence alignments and analysis of coevolving protein residues

Multiple sequence alignments (MSAs) of the T1R2s and T1R3s were performed with the ClustalW (version 1.83) program (Thompson et al., 1994). The coevolutionary relationship between two residues in the T1R2 and T1R3 families were analyzed based on the mutual information (MI), which was obtained according to the results of MSAs using the MISTIC web server (http://mistic.leloir.org.ar/index.php) (Simonetti et al., 2013). MI indicates the extent to which knowledge of the amino acid at one position can allow prediction of the amino acid at the other position.

### Molecular simulation of the sweet taste receptors

The homology model of full-length human T1R2/T1R3 was constructed using the Swiss-Model program (http://swissmodel.expasy.org/) with the heterodimeric human metabotropic GABA(B) receptor (PDB: 6UO8) as the template. A sequence alignment of the human T1R2 and T1R3 and template sequences was carried out with ClustalW, and the human T1R2 and T1R3 were uploaded as hetero targets for modeling, respectively. The resulted model was evaluated with the Verify 3D program with acceptable scores and was selected for following simulation (Luthy et al., 1992).

To construct the complex of aspartame and the modeled human T1R2/T1R3, molecular docking was performed using the Vina protocol in Yinfo Cloud Computing Platform (https://cloud.yinfotek.com/), with the previously well-characterized aspartame-receptor interactive residues (Y103, D142, Y215, P277, D278, L279, S303, D307, R383 and V384) as the constraints (Liu et al., 2011). The final docked complex was selected by binding energies and cluster analysis.

## Results

### Taxonomic distribution and classification of T1R2s and T1R3s in primates

**FIGURE 2:**
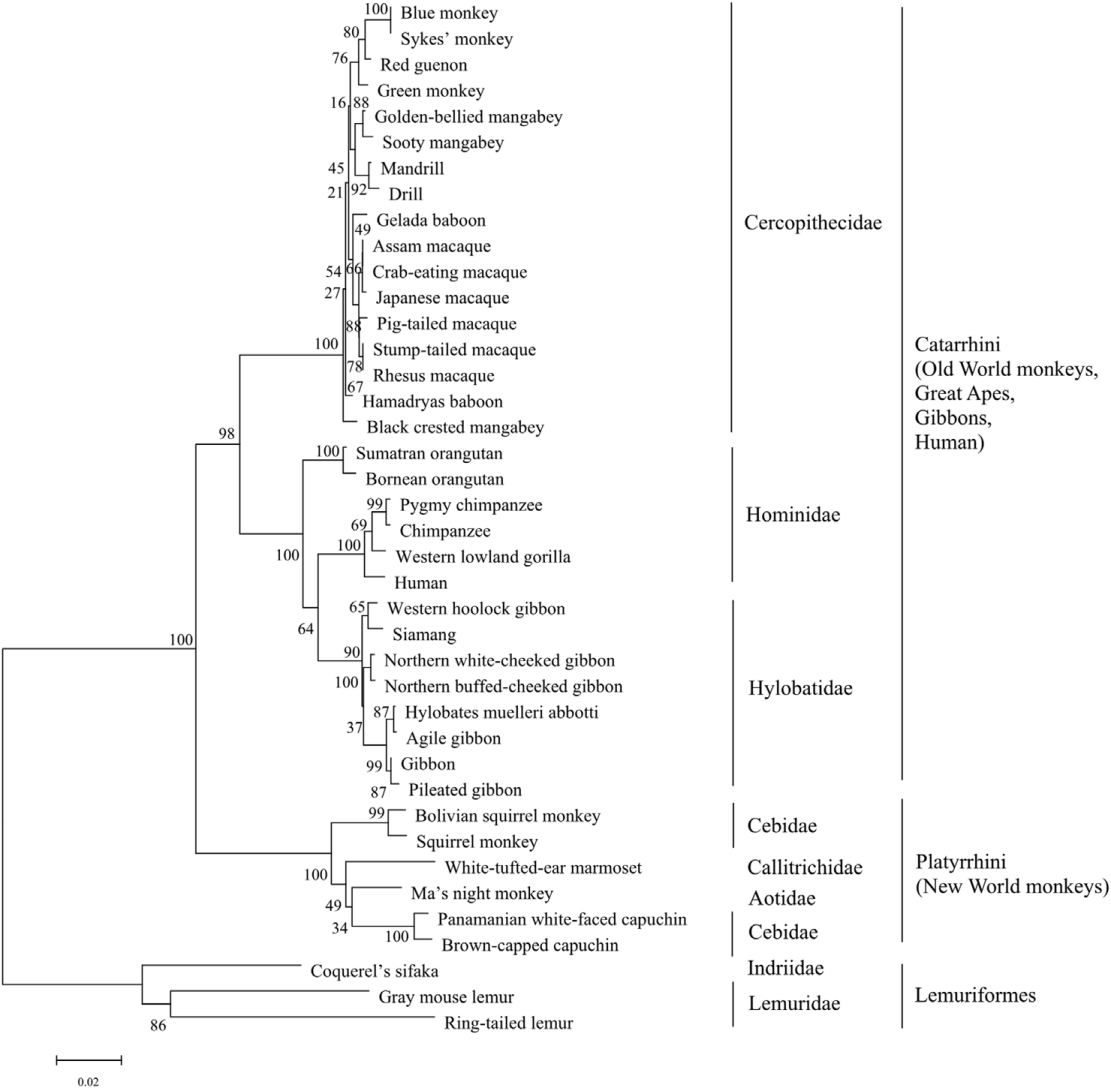
The phylogenetic tree analysis of sweet taste receptor T1R2 in primates. The percentages of replicate trees in which the associated taxa clustered together in the bootstrap test (1,000 replicates) are shown next to the branches, with branch lengths indicating the number of substitutions per site. The phyla and taxonomy of analyzed species are shown on the right.

**FIGURE 3:**
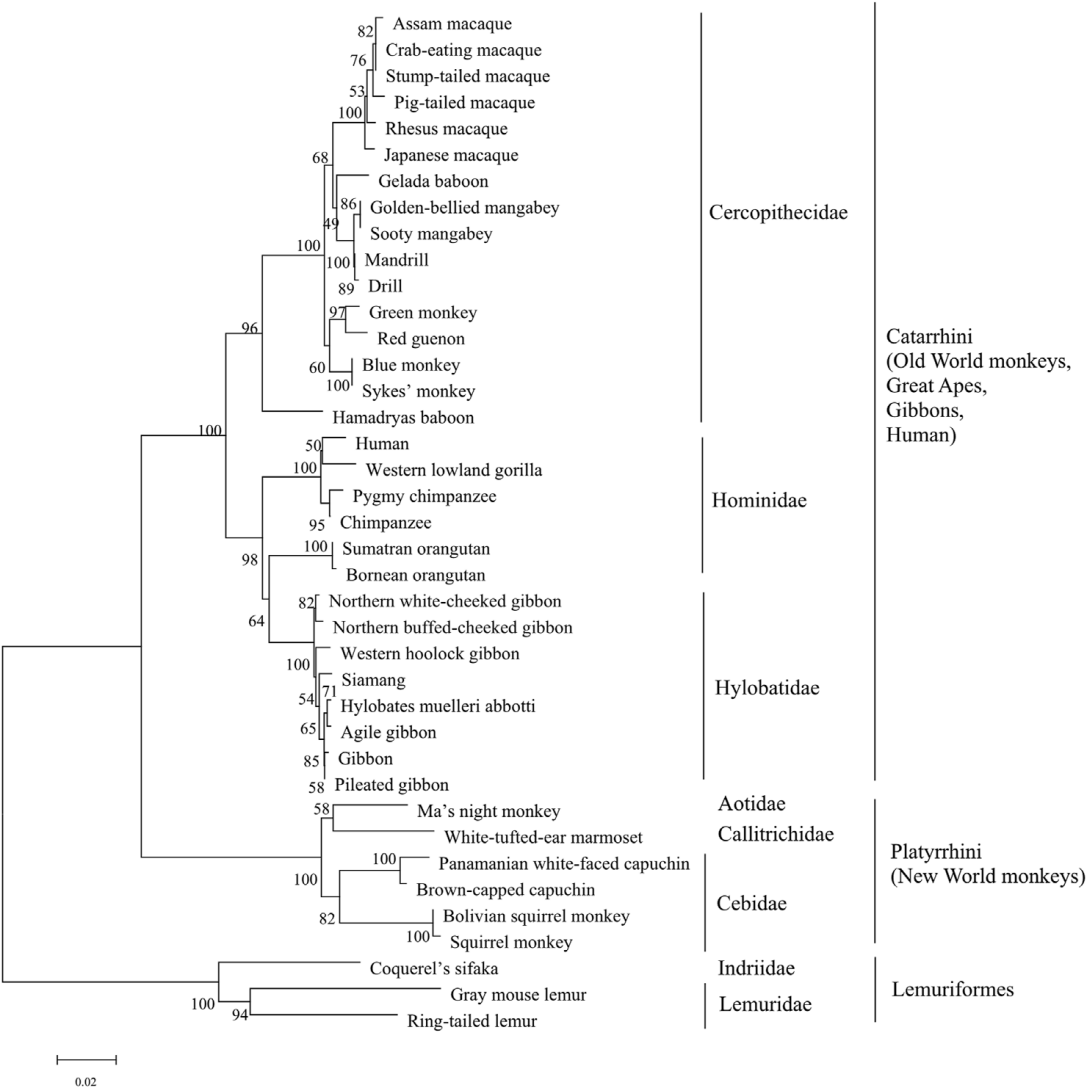
The phylogenetic tree analysis of sweet taste receptor T1R3 in primates. The percentages of replicate trees in which the associated taxa clustered together in the bootstrap test (1,000 replicates) are shown next to the branches, with branch lengths indicating the number of substitutions per site. The phyla and taxonomy of analyzed species are shown on the right.

Our initial search in the InterPro database revealed that mammals harboring sweet taste receptors could be devided into four categories: primates, ungulates, pterodactyls and carnivores. Moreover, the species in primate kingdom can be subclassified into three taxonomic groups which include simians Platyrrhini (New World monkeys), Catarrhini (Old World monkeys, great apes, gibbons and humans), and prosimians Lemuriformes (Figures 2, 3). All the T1R2 and T1R3 sequences in primates show the typical seven trans-membrane helix domain and nine conserved cysteine residues motif as revealed by the TMHMM and clustalW analysis, respectively, suggesting their intact functionality, although pseudogenization and sweet taste loss have been found in some other species, such as carnivora (Li et al., 2005).

To gain a detailed view of the evolutionary relationships, phylogenetic analysis was performed. The results show that the three taxonomic groups of T1R2s in primates mentioned above are well separated and clustered in the phylogenetic tree (Figure 2). All subgroups appear to be in separate clade in the phylogenetic tree, suggesting distinct evolutionary pressure and course for sweet taste recognition among these primate species. The varieties of ecological environment and adaption histories for these species could confer their multifarious sequence distributions of sweet taste receptors in evolution (Yarmolinsky et al., 2009; Jordan et al., 2015).

Phylogenetic analysis of T1R3s in primates was performed as that of T1R2s (Figure 3). The results show that similar clusters of taxonomic distribution in the phylogenetic tree for T1R3s as that for T1R2s, implying a coevolutionary history between T1R2 and T1R3, which is in accordance with the essential roles of each subunit for the integrated functionality of the heterodimeric sweet taste receptor T1R2/T1R3 (Li et al., 2002; Xu et al., 2004).

**FIGURE 4:**
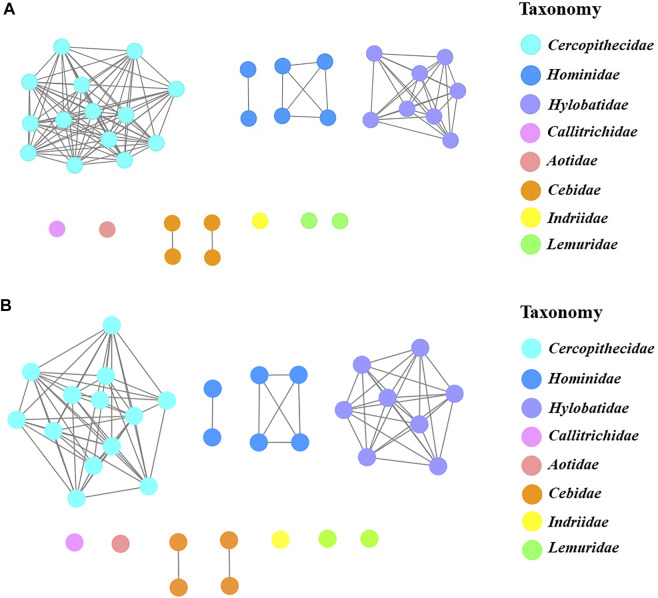
The protein sequence similarity networks (SSNs) of T1R2s and T1R3s in primates. **(A)** Protein sequences of T1R2s were analyzed to generate the network with an e-value threshold 10–5 (>96% sequence identity). The three nodes denoting T1R2 sequences of blue monkey and sykes’ monkey, rhesus macaque and stump-tailed macaque, and assam macaque and crab-eating macaque are overlapped respectively due to their same sequences. **(B)** Protein sequences of T1R3s were analyzed to generate the network with an e-value threshold 10–5 (>98% sequence identity). The three nodes denoting T1R3 sequences of blue monkey and sykes’ monkey, crab-eating macaque and stump-tailed macaque, and golden-bellied mangabey and sooty mangabey are overlapped respectively due to their same sequences. Each node represents one protein. Nodes from the same family in the networks are shown with the same color, and the colors corresponding to different families are listed on the right.

To further clarify the relationships among these sweet taste receptors, a SSN for the analyzed T1R2 or T1R3 sequences was constructed respectively by EFI-EST with an e-value threshold of 10–5 (Figure 4A) (Gerlt et al., 2015). Each protein was painted according to taxonomic classification. In each network, sequences above 96% (T1R2) or 98% (T1R3) identity were selected to draw an edge between nodes, and the proteins were classified into four clusters (with at least two edges) and four discrete groups. It is shown that all these clusters/groups belong to the same taxonomic classification. Furthermore, similar taxonomic distributions were found in primate T1R3s as that in T1R2s (Figure 4B). Moreover, the relationships of taxonomic distributions among T1R2s and T1R3s in primates identified in the SSNs are consistent with the results in phylogenetic tree analysis (Figures 2, 3), suggesting that the evolution of sweet taste receptors in primates have been very well-conserved.

### Analysis of the molecular determinants of T1R2s/T1R3s in primates for their function, diversity and evolution

#### Molecular determinants in T1R2

**FIGURE 5:**
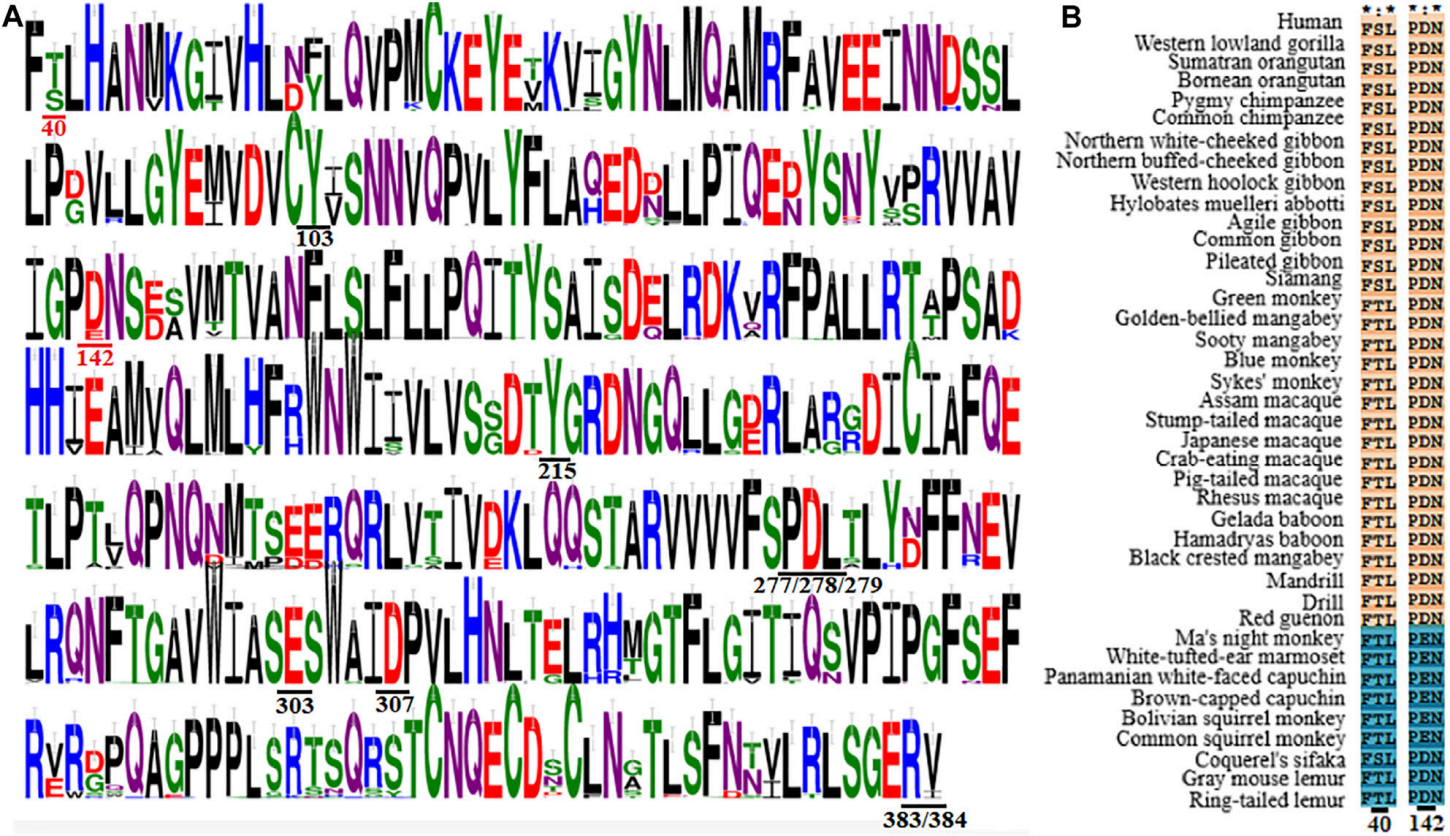
Multiple sequence alignment of T1R2s in primates. **(A)** Sequence logo representation of the conservation region and key residues mediating the sweet taste toward aspartame. The conservation level of each residue is indicated by the height of the bar above it. Residues involved in the sweet taste toward aspartame are underlined, and the two critical residues responsible for the species-dependent sweet taste toward aspartame are colored in red. This figuer was generated with the WebLogo 3 (https://weblogo.threeplusone.com/). **(B)** Sequence alignment of the two critical residues responsible for the species-dependent sweet taste toward aspartame. The proposed aspartame tasters are colored in light red while aspartame non-tasters are colored in blue.

It should be meaningful to elaborate the key receptor residues determining the species-dependent taste toward aspartame in primates based on previous findings and our present analysis. The MSA clearly show that most of the previously identified aspartame binding residues (Y103, Y215, P277, D278, L279, S303, D307, R383 and V384 in human T1R2) are conserved except the two critical residues at sites 40 and 142 described above (Figure 5; Supplementary Figure S1). Specifically, it was found that all species of Old World monkeys, great apes, gibbons and humans harbor D142 (taster type) while New World monkeys harbor E142 (non-taster type), in agreement with the crucial role of residue at site 142 for the switch of sensitivity to aspartame (Liu et al., 2011) as well as the previously reported species-dependent sweet taste in behavioral and electrophysiological tests (Hellekant et al., 1980; Nofre et al., 1996). Another key residue S40 mediating the intensity of sweet taste is conserved in great apes, gibbons and humans, whereas T40 is present in most of other analyzed primate species, which are consistent with the previous findings that humans and apes (Catarrhini) have the higher sensitivity to aspartame (lower threshold values) than monkeys (Platyrrhini) in behavioral tests (Hellekant and Danilova, 1996; Pereira et al., 2021).

Surprisingly, it is found that the three prosimians Lemuriformes species, which exhibited no sensitivity to aspartame in behavioral tests (Hellekant et al., 1981; Nofre et al., 1996), harbor the conserved crucial residue D142 as that of aspartame tasters, although another key residue at site 40 is variable (Figure 5). Notably, the species coquerel’s sifaka has the same two molecular determinative residues (D142 and S40) as aspartame tasters (Old World monkeys, great apes, gibbons and humans). These results suggest that prosimians Lemuriformes species could be aspartame tasters, which are contradictory to previous conclusions in behavioral tests (Hellekant et al., 1981; Nofre et al., 1996; Schilling et al., 2004). A plausible explanation is that the *in vitro* function of sweet taste receptor of some species may not fully reflect the behavioral outcome of response toward some sweeteners, such as an averse reaction that could be a positive response in cell-based assays (van Giesen et al., 2016). Alternatively, other residues could be involved in mediating the non-sensitivity to aspartame of sweet taste receptors in prosimians species (Chéron et al., 2019). Further investigations on the sensitivities of sweet taste receptors in prosimians species toward aspartame as well as their determinative residues should be interesting and informative.

#### Molecular determinants in T1R3

**FIGURE 6:**
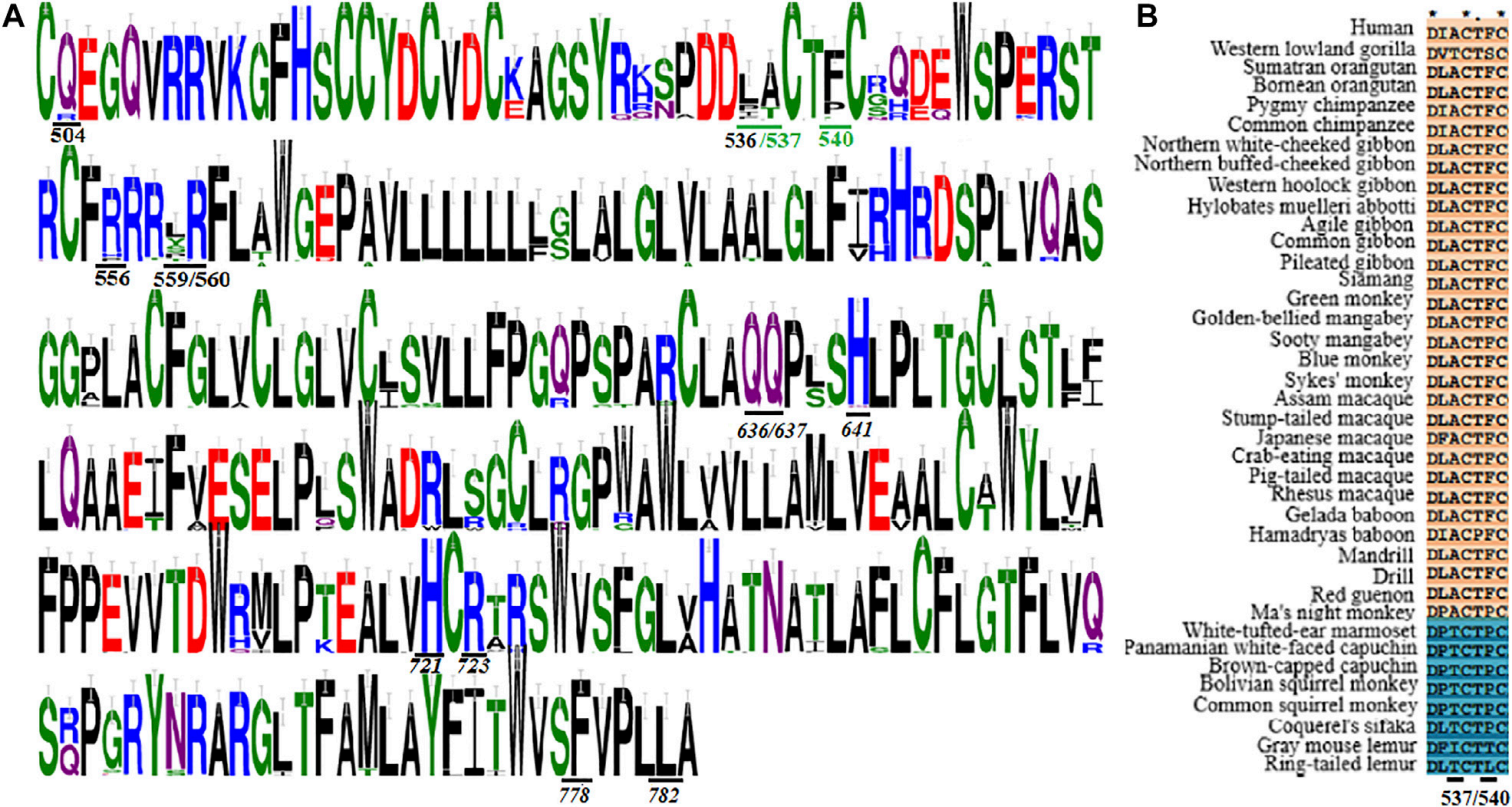
Multiple sequence alignment of T1R3s in primates. **(A)** Sequence logo representation of the conservation region and key residues mediating the sweet taste toward brazzein, thaumatin and cyclamate. The conservation level of each residue is indicated by the height of the bar above it. The two critical residues responsible for the species-dependent sweet taste toward brazzein are colored in green. This figuer was generated with the WebLogo 3 (https://weblogo.threeplusone.com/). **(B)** Sequence alignment of the two critical residues responsible for the species-dependent sweet taste toward brazzein. The proposed brazzein tasters are colored in light red while brazzein non-tasters are colored in blue.

Jiang et al. revealed the critical roles of residues A537 and F540 in the CRD of human T1R3 for the sensitivity to brazzein (Jiang et al., 2004). Consistent with this finding, MSA shows that A at human T1R3 site 537 (brazzein taster type) is conserved in Old World monkeys, great apes, gibbons and humans (sweet proteins tasters), while T at this site (brazzein non-taster type) is present in New World monkeys and other primate species (sweet proteins non-tasters) (Figure 6; Supplementary Figure S2). Residues at human site 540 exhibits similar distribution as site 537 while site 536 displays variable substitutions. However, two non-conservative exceptions, T537 and S540 of western lowland gorilla (Hominidae) and A537 of ma’s night monkey (New World monkeys) were found. Whether or not the two species respond to sweet-tasting protein brazzein remains unknown. The other identified residues (Q504, R556, S559 and R560) in Masuda et al.’s study are almost conserved except few substitutions, with S559 showing a greater extent substitution (Figure 6) (Masuda et al., 2013). Together, the sequence conservation and substitution could generally account for the reported species-dependent taste toward sweet-tasting proteins in primates in previous behavioral studies, thus enabling one to predict the responses of some species based on both previous findings and present sequence analysis.

Cyclamate is an artificial sweetener which has been identified to bind within the TMD of human T1R3 (Jiang et al., 2005b). The potential cyclamate binding pocket includes Q636, Q637, H641, H721, R723, F778 and L782. However, we found that all these residues are conserved in the T1R3s of analyzed primate species except that R723 is replaced by C for white-tufted-ear marmoset (Figure 6; Supplementary Figure S2). Previous behavioral and physiological studies have indicated that cyclamate could only be perceived by Old World monkeys, apes, gibbons and humans, but not by New World monkeys and other primates (Nofre et al., 1996). Therefore, it appears that the proposed residues aforesaid in the binding pocket are insufficient to account for the species-dependent taste toward cyclamate. In support of this, it has been widely accepted that other regions beyond the binding sites can also modulate the responses toward sweeteners *via* allosteric regulation, thus determining the sensitivity (Chéron et al., 2019). Moreover, although the potential binding sites of some other sweeteners (e.g., neohesperidin dihydrochalcone and saccharin) have been identified (Winnig et al., 2007; Masuda et al., 2012), no corresponding behavioral or physiological results are available now to interpret these mechanical findings, and an accurate elucidation of the relationships between the molecular determining residues and species-dependent sweet taste should wait for resolution of the spatial structures of sweetener-receptor complexes in the future.

### Analysis of coevolving residues in primate T1R2s/T1R3s

The residues involved in aspartame, cyclamate and sweet-tasting proteins recognition described above that exhibit almost conserved taxonomic distribution, cooperatively mediate the species-dependent taste toward these sweeteners, suggesting their interactive and coevolving relationships. To gain a deeper insight for the diversity and relationship among residues of sweet taste receptors in primates, sequence mutual information (MI) analysis of T1R2s and T1R3s was carried out, and the results are shown in Supplementary Figure S3. In this figure, A and C show the conserved and coevolving residues of T1R2s and T1R3s, and B and D show the residues with high cumulative MI (cMI) values form a connected distance network, respectively, indicating that these residues share a high MI score and are evolutionarily correlated. These results suggest significant conversation and coevolution of specific amino acids of sweet taste receptors in primates during their evolution.

It should be informative to relate the MI signal to the molecular determinant residues of T1R2s/T1R3s in primates. Most of the residues at the positions of human T1R2 (S40, Y103, D142, Y215, P277, D278, L279, S303, D307, R383 and V384) involved in aspartame recognition display considerable MI values with other residues, suggesting their coevolving interrelationships (Supplementary Data Sheet S2). Moreover, the two critical residues at human T1R2 sites (S40 and D142) for species-dependent taste toward aspartame exhibit high cMI scores 883.8 and 909.8, respectively (Table 1), highlighting their significant degree of shared mutual information and implying their important roles in the evolution of sweet taste receptors in primates. Similar patterns are also found for the determinative residues in sweet-tasting proteins recognition that Q504, I536, A537, F540, R556, and S559 at the positions of human T1R3 show remarkable cMI scores, and considerable MI values of these residues are also present (Table 1, Supplementary Data Sheet S3).

**TABLE 1:** cMI (cumulative mutual information) scores of crucial residues in T1R2s and T1R3s.

Subunit	Position in human sweet taste receptor	cMI scores
T1R2	S40	883.798002
	D142	909.760677
	V384	452.2915
	Y103, Y215, P277, D278, L279, S303, D307, R383	0
T1R3	Q504	669.414593
	I536	1075.411256
	A537	719.624656
	F540	1268.504506
	R556	94.624677
	S559	1886.547787
	R560, Q636, Q637, H641, H721, R723, F 778, L782	0

For the residues at the binding of cyclamate located at human T1R3 (Q636, Q637, H641, H721, R723, F778 and L782), it turns out that none of them display observable cMI scores (Table 1), which could be related to the fact that these residues are not involved in the species-dependent taste toward cyclamate. Furthermore, essential roles of the two T1R subunits for the functional integrity of the sweet taste receptor have been described (Xu et al., 2004), and previous studied have indicated that mutations of residues in the binding site of one sweetener located at one monomer could also affect the sensitivity of other sweeteners which bind at another monomer of the heterodimeric T1R2/T1R3 (Jiang et al., 2005a; b; Masuda et al., 2012; Yang et al., 2021). Studies on the dimeric GPCRs have revealed that cross-talk between two monomers determines receptor activation and signal integration (Rives et al., 2009). Therefore, it could be proposed that coevolution of residues between T1R2 and T1R3 as well as their interplay play significant roles for the overall activity of T1R2s/T1R3s. Lastly, it should be noted that our MI analysis can only draw a general and rough profile for predicting the interrelationships of residues, rather than precise correlations between specific positions. Nevertheless, this analysis provides helpful guidelines for better understanding the coevolutionary signal contained within sweet taste receptor families and for further investigating the molecular basis of species-dependent sweet taste in primates toward various sweeteners.

### Epistasis in the evolution of sweet taste receptors in primates

Epistasis means that the phenotypic consequences of a mutation depend on the genetic background (genetic sequence) in which it occurs (Poelwijk et al., 2007). The probable epistasis among residues of sweet taste receptors in primates could be deduced from a few of functional mutagenesis studies of T1R2s/T1R3s. For example, analysis of the magnitude of activities of squirrel monkey to human T1R2 mutations (T40S and E142D) could infer obvious epistasis in the probable evolutionary trajectory for sensitivity to aspartame, as the order of intensities of acquired sensitivity in squirrel monkey to human T1R2 mutations were E142D/T40S (increased) > E142D (acquired) > T40S (no response) (Liu et al., 2011). Similar epistatic effect was also found in the mouse to human T1R3 mutations for acquired sensitivity to sweet-tasting protein brazzein, as shown by an order of intensities of sensitivities in these mutations T542A/F541I/P545F > T542A/P545F > T542A > P545F (no response) (Jiang et al., 2004). To our knowledge, epistasis in the evolution of sweet taste receptors has not been reported up to now due to the relatively less information of the function of mutations for these receptors, thus further research on this topic should be informative for understanding the molecular mechanism of evolution of sweet taste in primates.

### Structural analysis of the molecular determinant residues of T1R2s/T1R3s in primates

Molecular modeling and docking were performed to elucidate the structural basis of key residues determining the species-dependent taste. As shown in Figure 1B, aspartame binds into a pocket *via* its interactions with the receptor residues. The two critical residues S40 and D142 are located near the binding site of aspartame, and the D142 can interact with the O1 atom of aspartame *via* hydrogen bonds bridge of a water molecule. Replacement of D142 by E (font in blue) that has a larger side chain in the squirrel monkey T1R2 could presumably reduce the volume of the binding pocket, leading to a failure of entrance into the site for aspartame. Moreover, S40 interacts with E142 *via* a hydrogen bond, which could stabilize the appropriate conformation of E142 for aspartame binding, thus exhibiting a cooperative role with D142 in aspartame recognition (Liu et al., 2011; Maillet et al., 2015).

Figure 1A shows the binding with sweet-tasting proteins brazzein or thaumatin in the CRD region of the sweet taste receptor. It is noteworthy that the two proteins could probably interact with the receptor *via* long distance surface charge complementarity due to their large bulk/volume, as proposed by the wedge model (Temussi, 2011). The binding pocket of another sweetener cyclamate, which has been characterized by Jiang et al. (Jiang et al., 2005b), is shown in Figure 1C. The key residues involved in recognizing different sweet substances and their structural properties are listed in Supplementary Table S1.

## Discussion

In this research, we carried out a comprehensive sequence, structure and function analysis of the sweet taste receptors-heterodimeric T1R2s/T1R3s in primates. Our results show well taxonomic distribution and classification of these receptors, as illustrated by phylogenetic tree and sequence similarity networks analysis (Figures 2–4). Furthermore, we reveal that the conservation/variation of molecular determinant residues of sweet taste receptors in primates, whose functions have been identified in previous mutagenesis experiments, can account for the previously reported behavioral or physiological results of sweet taste in primates toward several typical sweeteners to a large extent. These findings illuminate the relationships between the sequence/structure of sweet taste receptors in primates and their functional roles for taste perception. Nevertheless, a few exceptions were found that the prosimians Lemuriformes species, which were reported to have no sensitivity to aspartame in behavioral test (Glaser et al., 1995), harbor the similar aspartame-tasting determinative residues (D142 and S40) as aspartame tasters (Old World monkeys, great apes, gibbons and humans) (Figure 5). Our lab is now working on the function of T1R2/T1R3 in these prosimians species to unravel this intriguing discrepancy.

The sweet taste receptor belongs to the class C GPCRs. By quantitatively mapping the global network of amino acid interactions in GPCRs, a comparative analysis has revealed a conserved network of non-covalent contacts that defines the GPCR fold (named as “molecular signatures”) (Venkatakrishnan et al., 2013), and a small subset of residues forms physically connected networks (named as “protein sectors”) that link distant functional sites, while each sector has a distinct functional role (Süel et al., 2003; Halabi et al., 2009). Therefore, the small subsets of residues responsible for the species-dependent taste toward sweeteners described in present study could be regarded as specific “protein sectors” or “molecular signatures” of sweet taste receptors. Furthermore, because sweet taste is a complex process involving many signal transduction pathways, we propose that future studies should apply multidisciplinary approaches in physiology, biochemistry, neuroscience, biophysics and evolutionary biology to elucidate the molecular mechanism of diversity, function and evolution of sweet taste receptors in primates as well as other mammals species (Yang et al., 2021). The schemes “molecular signatures” and “protein sectors” of GPCRs highlighted above thus could provide meaningful strategies for further investigation on these topics.

## Data Availability

The datasets presented in this study can be found in online repositories. The names of the repository/repositories and accession number(s) can be found in the article/Supplementary Material.
