# Supplementary material for: Systematic analysis reveals novel insight into the molecular determinants of function, diversity and evolution of sweet taste receptors T1R2/T1R3 in primates
Source: Front Mol Biosci. 2023 Jan 25;10:1037966. doi: 10.3389/fmolb.2023.1037966 (PMC9905694; doi:10.3389/fmolb.2023.1037966)
Supplement: Supplementary file 1 [file DataSheet4.pdf]

```
                                  *      .. * :** :    *  ***:*.*********:****:** ***::**** ***** *: ****** ***:******.*.***.* ****:*****:******.*****:.: :***:*:**.* .********:**::* **
Human                             MGPRAKTISSLFFLLWVLAEPAENSDFYLPGDYLLGGLFSLHANMKGIVHLNFLQVPMCKEYEVKVIGYNLMQAMRFAVEEINNDSSLLPGVLLGYEIVDVCYISNNVQPVLYFLAHEDNLLPIQEDYSNYISRVVAVIGPDNSESVMTV   150
Western lowland gorilla           MGTRATTICSLFFLLWVLAEPAENSDFYLPGDYLLGGLFSLHANMKGIVHLNFLQVPMCKEYEVKVIGYNLMQAMRFAVEEINNDSSLLPGVLLGYEIVDVCYISNNVQPVLYFLAHEDNLLPIQEDYSNYISRVVAVIGPDNSESVMTV   150
Sumatran orangutan                MGPRATTICSLFFLLWVLAEPAENSDFYLPGDYLLGGLFSLHANMKGIVHLNFLQVPMCKEYEVKVIGYNLMQAMRFAVEEINNDSSLLPGVLLGYEMVDVCYVSNNVQPVLYFLAHEDNLLPIQEDYSDYVSRVVAVIGPDNSESVMTV   150
Bornean orangutan                 MGPRATTICSLFFLLWVLAEPAENSDFYLPGDYLLGGLFSLHANMKGIVHLNFLQVPMCKEYEVKVIGYNLMQDMRFSVEEINNDSSLLPGVLLGYEMVDVCYVSNNVQPVLYFLAHEDNLLPIQEDYSDYVSRVVAVIGPDNSESVMTV   150
Pygmy chimpanzee                  MGPRAKTICSLFFLLWVLAEPAENSDFYLPGDYLLGGLFSLHANMKGIVHLNFLQVPMCKEYEVKVIGYNLMQAMRFAVEEINNDSSLLPGVLLGYEIVDVCYISNNVQPVLYFLAHEDNLLPIQEDYSNYISRVVAVIGPDNSESVMTV   150
Common chimpanzee                 MGPRAKTICSLFFLLWVLAEPAENSDFYLPGDYLLGGLFSLHANMKGIVHLNFLQVPMCKEYEVKVIGYNLMQAMRFAVEEINNDSSLLPGVLLGYEIVDVCYISNNVQPVLYFLAHEDNLLPIQEDYSNYISRVVAVIGPDNSESVMTV   150
Northern white-cheeked gibbon     MGPRATTICSLFFLLRILSEPAENSDFYLPGDYLLGGLFSLHANMKGIVHLNFLQVPMCKEYEVKVIGYNLMQAMRFAVEEINNDSSLLPGVLLGYEIVDVCYISNNVQPVLYFLAHEDNLLPIQEDYSNYSSRVVAVIGPDNSESVITV   150
Northern buffed-cheeked gibbon    MGPRATTICSLFFLLRILSEPAENSDFYLPGDYLLGGLFSLHANMKGIVHLNFLQVPMCKEYEVKVIGYNLMQAMRFAVEEINNDSSLLPGVLLGYEIVDVCYISNNVQPVLYFLAHEDNLLPIQEDYSNYSSRVVAVIGPDNSESVMTV   150
Western hoolock gibbon            MGPRAMTICSLFFLLRILSESAENSDFYLPGDYLLGGLFSLHANMKGIVHLNFLQVPMCKEYEVKVIGYNLMQAMRFAVEEINNDSSLLPGVLLGYEIVDVCYISNNVQPVLYFLAHEDNLLPIQEDYSNYSSRVVAVIGPDNSESVMTV   150
Hylobates muelleri abbotti        MGPRATTICSLFFLLRILSESAENSDFYLPGDYLLGGLFSLHANMKGIVHLNFLQVPMCKEYEVKVIGYNLMQAMRFTVEEINNDSNLLPGVLLGYEIVDVCYISNNVQPVLYFLAHEDNLLPIQEDYSNYSSRVVAVIGPDNSESVMTV   150
Agile gibbon                      MGPRATTICSLFFLLRILSESAENSDFYLPGDYLLGGLFSLHANMKGIVHLNFLQVPMCKEYEVKVIGYNLMQAMRFTVEEINNDSNLLPGVLLGYEIVDVCYISNNVQPVLYFLAHEDNLLPIQEDYSNYSSRVVAVIGPDNSESVMTV   150
Common gibbon                     MGPRATTICSLFFLLRILSESAENSDFYLPGDYLLGGLFSLHANMKGIVHLNFLQVPMCKEYEVKVIGYNLMQAMRFAVEEINNDSNLLPGVLLGYEIVDVCYISNNVQPVLYFLAHEDNLLPIQEDYSNYSSRVVAVIGPDNSESVTTV   150
Pileated gibbon                   MGPRATTICSLFFLLRILSESAENSDFYLPGDYLLGGLFSLHANMKGIVHLNFLQVPTCKEYEVKVIGYNLMQAMRFAVEEINNDSNLLPGVLLGYEIVDVCYISNNVQPVLYFLAHEDNLLPIQEDYSNYSSRVVAVIGPDNSESVTTV   150
Siamang                           MGPRAMTICSLFFLLRILSEPAENSDFYLPGDYLLGGLFSLHANMKGIVHLNFLQVPMCKEYEVKVIGYNLMQAMRFAVEEINNDSSLLPGVLLGYEIVDVCYISNNVQPVLYFLAHEDNLLPIQEDYSNYSSRVVAVIGPDNSESVLTV   150
Green monkey                      MRPRATTICSLFFLLQVLAEPAKNSDFYLPGDYLLGGLFTLHANMKGIVHLDYLQVPMCKEYETKVIGYNLMQAMRFAVEEINNDSSLLPDVLLGYEMVDVCYVSNNVQPVLYFLAQEDDLLPIQENYSNYVPRVVAVIGPDNSDAVMTV   150
Golden-bellied mangabey           MRPRATTICSLFFLLQVLAEPAKNSDFYLPGDYLLGGLFTLHANMKGIVHLDYLQVPMCKEYETKVIGYNLMQAMRFAVEEINNDSSLLPDVLLGYEMVDVCYVSNNVQPVLYFLAQEDDLLPIQENYSNYVPRVVAVIGPDNSDAVMTV   150
Sooty mangabey                    MRPRATTICSLFFLLQVLAEPAKISDFYLPGDYLLGGLFTLHANMKGIVHLDYLQVPMCKEYETKVIGYNLMQAMRFAVEEINNDSSLLPDVLLGYEMVDVCYVSNNVQPVLYFLAQEDDLLPIQENYSNYVPRVVAVIGPDNSDAVMTV   150
Blue monkey                       MRPRATTICSLFFLLQVLAEPAKNSDFYLPGDYLLGGLFTLHANVKGIVHLDYLQVPMCKEYETKVIGYNLMQAMRFAVEEINNDSSLLPDVRLGYEMVDVCYISNNVQPVLYFLAQEDDLLPIQENYSNYVPRVVAVIGPDNSDAVMTV   150
Sykes' monkey                     MRPRATTICSLFFLLQVLAEPAKNSDFYLPGDYLLGGLFTLHANVKGIVHLDYLQVPMCKEYETKVIGYNLMQAMRFAVEEINNDSSLLPDVRLGYEMVDVCYISNNVQPVLYFLAQEDDLLPIQENYSNYVPRVVAVIGPDNSDAVMTV   150
Assam macaque                     MRPRATTICSLFFLLRVLAEPAKNSDFYLPGDYLLGGLFTLHANMKGIVHLDYLQVPMCKEYETKVIGYNLMQAMRFAVEEINNDSSLLPDVLLGYEMVDVCYVSNNVQPVLYFLAQEDDLLPIQENYSNYVPRVVAVIGPDNSDAVMTV   150
Stump-tailed macaque              MRPRATTICSLFFLLRVLAEPAKNSDFYLPGDYLLGGLFTLHANMKGIVHLDYLQVPMCKEYETKVIGYNLMQAMRFAVEEINNDSSLLPDVLLGYEMVDVCYVSNNVQPVLYFLAQEDDLLPIQENYSNYVPRVVAVIGPDNSDAVMTV   150
Japanese macaque                  MRPRATTICSLFFLLWVLAEPAKNSDFYLPGDYLLGGLFTLHANMKGIVHLDYLQVPMCKEYETKVIGYNLMQAMRFAVEEINNDSSLLPDVLLGYEMVDVCYVSNNVQPVLYFLAQEDDLLPIQENYSNYVPRVVAVIGPDNSDAVMTV   150
Crab-eating macaque               MRPRATTICSLFFLLRVLAEPAKNSDFYLPGDYLLGGLFTLHANMKGIVHLDYLQVPMCKEYETKVIGYNLMQAMRFAVEEINNDSSLLPDVLLGYEMVDVCYVSNNVQPVLYFLAQEDDLLPIQENYSNYVPRVVAVIGPDNSDAVMTV   150
Pig-tailed macaque                MRPRATTICSLFFLLRVLAEPAKNSDFYLPGDYLLGGLFTLHANMKGIVHLDYLQVPMCKEYETKVIGYNLMQAMRFAVEEINNDSSLLPDVLLGYEMVDVCYVSNNVQPVLYFLAQEDDLLPIQENYSNYVPRVVAVIGPDNSDAVMTV   150
Rhesus macaque                    MRPRATTICSLFFLLRVLAEPAKNSDFYLPGDYLLGGLFTLHANMKGIVHLDYLQVPMCKEYETKVIGYNLMQAMRFAVEEINNDSSLLPDVLLGYEMVDVCYVSNNVQPVLYFLAQEDDLLPIQENYSNYVPRVVAVIGPDNSDAVMTV   150
Gelada baboon                     MRPRETTICSLFFLLRVLAEPAKNSDFYLPGDYLLGGLFTLHANVKGIVHLDYLQVPMCKEYETKVIGYNLMQAMRFAVEEINNDSSLLPDVLLGYEMVDVCYVSNNVQPVLYFLAQEDDLLPIQENYSNYVPRVVAVIGPDNSDAVMTV   150
Hamadryas baboon                  MRPRATTICSLFFLLRVLAEPAKNSDFYLPGDYLLGGLFTLHANMKGIVHLDYLQVPMCKEYETKVIGYNLMQAMRFAVEEINNDSSLLPDVLLGYEMVDVCYVSNNVQPVLYFLAQEDDLLPIQENYSNYVSRVVAVIGPDNSDAVMTV   150
Black crested mangabey            MRPRATTICSLFFLLRVLAEPAKNSDFYLPGDYLLGGLFTLHANMKGIVHLDYLQVPMCKEYETKVIGYNLMQAMRFAVEEINNDSSLLPDVLLGYEMVDVCYVSNNVQPVLYFLAQEDDLLPIQENYSNYVPRVVAVIGPDNSDAVMTV   150
Mandrill                          MRPRATTICSLFFLLRVLAEPAKNSDFYLPGDYLLGGLFTLHANMKGIVHLDYLQVPMCKEYETKVIGYNLMQAMRFAVEEINNDSSLLPDVLLGYEMVDVCYVSNNVQPVLYFLAQEDDLLPIQENYSNYMPRVVAVIGPDNSDAVMTV   150
Drill                             MRPRATTICSLFFLLQVLAEPAKNSDFYLPGDYLLGGLFTLHANMKGIVHLDYLQVPMCKEYETKVIGYNLMQAMRFAVEEINNDSSLLPDVLLGYEMVDVCYVSNNVQPVLYFLAQEDDLLPIQENYSNYMPRVVAVIGPDNSDAVMTV   150
Red guenon                        MRPRATTICSLFFLLQVLAEPAKNSDFYLPGDYLLGGLFTLHANMKGIVHLDYLQVPMCKEYETKVIGYNLMQAMRFAVEEINNDSSLLPDVLLGYEMVDVCYVSNNVQPVLYFLAQEDDLLPIQENYSNYVPRVVAVIGPDNSDAVMTV   150
Ma's night monkey                 MGSRARTVCFLFFLLWVLAEPAENSDFHLPGDYLLGGLFTLHANMKGTVHLNFLQVPMCKEYEMKVSGYNLMQAMRFAVEEINNDSSLLPDVLLGYEMVDVCYISNNVQPVLYFLAQEDSLLPIQEDYSNYVPRVVAVIGPENSESVTTV   150
White-tufted-ear marmoset         MGPRARTVCFLFFLLWVLAELAENSDFHLPGDYLLGGLFTLHANMKGIVHLNFLQVPMCKEYEMKVSGYNLMQAMRFAVEEINNDSSLLPNVLLGYEMVDVCYISNNVQPALYFLAQEDNLLPIREDYSNYVPRVVAVIGPENSESVMTV   150
Panamanian white-faced capuchin   MGPRARTVCFLFFLLWVLAELAENSDFHLPGDYLLGGLFTLHANMKGTVHLNFLQVPMCKEYEMKVSGYNLMQAMRFAVEEINNDSSLLPGVLLGYEMVDVCYISNNVQPVLYFLAQEDSLLPIQEDYSNYVPRVVAVIGPENSESVMTV   150
Brown-capped capuchin             MGPRVRTVCFLFFLLWVLAEPAETSDFHLPGDYLLGGLFTLHANMKGTVHLNFLQVPMCKEYEMKVSGYNLMQAMRFAVEEINNDSSLLPDVLLGYEMVDVCYISNNVQPVLYFLAQEDSLLPIQEDYSNYVPRVVAVIGPENSESVMTV   150
Bolivian squirrel monkey          MEPRVRTVCFLFFLLWVLAEPAENSDFHLPGDYLLGGLFTLHANMKGIVHLNFLQVPMCKEYEVKLSGYNLMQAMRFAVEEINNDSSLLPDVRLGYEMVDVCYVSNNVQPVLYFLAQEDNLLPIQEDYSNYVPRVVAVIGPENSESVTTV   150
Common squirrel monkey            MEPRVRTVCFLFFLLRVLAEPAKNSDFYLPGDYLLGGLFTLHANMKGTVHLNFLQVPMCKEYEVKLSGYNLMQAMRFAVEEINNDSSLLPDVRLGYEMVDVCYVSNNVQPVLYFLAQEDNLLPIQEDYSNYVPRVVAVIGPENSESVTTV   150
Coquerel's sifaka                 MGRWARMICSLFLLLQVLVEPAENSDFYLTGDYLLGGLFSLHANVKGTVHLNYLQVPKCKEYEMKVLGYNLMQAMRFAVEEINNHSSLLPGVLLGYEMVDVCYISNNVQPVLYFLAQKDYFLPIQEDYSSYVPRVVAVIGPDNSESVVTV   150
Gray mouse lemur                  MGPWARMVCSLSLLLQVPAGPAESSDFYLAGDYLLGGLFTLHANVKGTVHLNYLQVPKCKEYEMKVLGYNLMQAMRFAVEEINNHSSLLPGVQLGYEMVDVCYISNNVQPVLYFLAREDYFLPIKEDYSQYVPRVVAVIGPDNSESVVTV   150
Ring-tailed lemur                 MGPWARVFCSLFLLLQVLAEPAGNSDFYLAGDYLLGGLFTLHANVKGIVHLNYLQVPKCKEYEMKVLGYNLMQAMRFAVEEINNHSSLLPGVVLGYEMVDVCYVSNNVQPVLYFLAQDNYFLPIQEDYSQYVPRVVAVIGPDNSEAVVTV   150
```

```
                               *:***************.*:*::* *:****** ***.**:**:.**:*:*:**** ****.* ******* *.:*:.  ********:**. *.**   .::: ** :**:***::*********** *:.** **:* *:**.****
Human                          ANFLSLFLLPQITYSAISDELRDKVRFPALLRTTPSADHHIEAMVQLMLHFRWNWIIVLVSSDTYGRDNGQLLGERVARRDICIAFQETLPTLQPNQNMTSEERQRLVTIVDKLQQSTARVVVVFSPDLTLYHFFNEVLRQNFTGAVWIA   300
Western lowland gorilla        ANFLSLFLLPQITYSAISDELQDKVRFPALLRTTPSADHHVEAMVQLMLHFRWNWIIVLVSSDTYGRDNGQLLGERLARRDICIAFQETLPTLQPNQNMTSEERQRLVTIVDKLQQSTARVVVVFSPDLSLYDFFNEVLRQNFTGAVWIA   300
Sumatran orangutan             ANFLSLFLLPQITYSAISDELRDKVRFPALLRTTPSADHHIEAMVQLMLHFRWNWIIVLVSSDTYGRDNGQLLGERLARRDICIAFQETLPTLQPNQNMTSEERQRLVTIVDKLQQSTARVVVVFSPDLTLYDFFNEVLRQNFTGAVWIA   300
Bornean orangutan              ANFLSLFLLPQITYSAISDELRDKVRFPALLRTTPSADHHIEAMVQLMLHFRWNWIIVLVSSDTYGRDNGQLLGERLARRDICIAFQETLPTLQPNQNMTSEERQRLVTIVDKLQQSTARVVVVFSPDLTLYDFFNEVLRQNFTGAVWIA   300
Pygmy chimpanzee               ANFLSLFLLPQITYSAIGDELRDKVRFPALLRTTPSADHHVEAMVQLMLHFRWNWIIVLVSSDTYGRDNGQLLGERLARRDICIAFQETLPALQPNQNMTSEERQRLVTIVDKLQQSTARVVVVFSPDLSLYDFFNEVLRQNFTGAVWIA   300
Common chimpanzee              ANFLSLFLLPQITYSAIGDELRDKVRFPALLRTTPSADHHVEAMVQLMLHFRWNWIIVLVSSDTYGRDNGQLLGERLARRDICIAFQETLPALQPNQNMTSEERQRLVTIVDKLQQSTARVVVVFSPDLSLYDFFNEVLRQNFTGAVWIA   300
Northern white-cheeked gibbon  ANFLSLFLLPQITYSAISDELRDKVRFPALLRTTPSADHHIEAMVQLMLHFRWNWIIVLVSSDTYGRDNGQLLGERLARRDICIAFQETLPTLQPNQNMTSDERQRLVTIVDKLQQSTARVVVVFSPDLTLYDFFNEVLRQNFTGAVWIA   300
Northern buffed-cheeked gibbon ANFLSLFLLPQITYSAISDELRDKVRFPALLRTTPSADHHIEAMVQLMLHFRWNWIIVLVSSDTYGRDNGQLLGERLARRDICIAFQETLPTLQPNQNMTSDERQRLVTIVDKLQQSTARVVVVFSPDLTLYDFFNEVLRQNFTGAVWIA   300
Western hoolock gibbon         ANFLSLFLLPQITYSAISDELRDKARFPALLRTTPSADHHIEAMVQLMLHFHWNWIIVLVSSDTYGRDNGQLLGERLARRDICIAFQETLPTLQPNQNMTSDERQRLVTIVDKLQQSTARVVVVFSPDLTLYDFFNEVLRQNFTGAVWIA   300
Hylobates muelleri abbotti     ANFLSLFLLPQITYSAIGDELRDKARFPALLRTTPSADHHIEAMVQLMLHFRWNWIIVLVSSDTYGRDNGQLLGERLARRDICIAFQETLPTLQPNQNMTSDERQRLVTIVDKLQQSTARVVVVFSPDLTLYDFFNEVLRQNFTGAVWIA   300
Agile gibbon                   ANFLSLFLLPQITYSAIGDELRDKARFPALLRTTPSADHHIEAMVQLMLHFRWNWIIVLVSSDTYGRDNGQLLGERLARRDICIAFQETLPTLQPNQNMTSDERQRLVTIVDKLQQSTARVVVVFSPDLTLYDFFNEVLRQNFTGAVWIA   300
Common gibbon                  ANFLSLFLLPQITYSAISDELRDKARFPALLRTTPSADHHIEAMVQLMLHFRWNWIIVLVSSDTYGRDNGQLLGERLARRDICIAFQETLPTLQPNQNMTSDERQRLVTIVDKLQQSTARVVVVFSPDLTLYDFFNEVLRQNFTGAVWIA   300
Pileated gibbon                ANFLSLFLLPQITYSAISDELRDKARFPALLRTTPSADHHIEAMVQLMLHFRWNWIIVLVSSDTYGRDNGQLLGERLARRDICIAFQETLPTLQPNQNMTSDERQRLVTIVDKLQQSTARVVVVFSPDLTLYDFFNEVLRQNFTGAVWIA   300
Siamang                        ANFLSLFLLPQITYSAISDELRDKARFPALLRTTPSADHHIEAMVQLMLHFRWNWIIVLVSSDTYGRDNGQLLGERLARRDICIAFQETLPTLQPNQNMTSDERQRLVTIVDKLQQSTARVVVVFSPDLTLYDFFNEVLRQNFTGAVWIA   300
Green monkey                   ANFLSLFLLPQITYSAISDELRDKVRFPALLRTAPSADHHIEAIVQLMLHFRWNWIIVLVSGDTYGRDNGQLLGDRLARGDICIAFQETLPTVQPNQNMTSEERQRLVTIVDKLQQSTARVVVVFSPDLTLYNFFNEVLRQNFTGAVWIA   300
Golden-bellied mangabey        ANFLSLFLLPQITYSAISDELRDKVRFPALLRTAPSADHHIEAMVQLMLHFHWNWIIVLVSGDTYGRDNGQLLGDRLARGDICIAFQETLPTVQPNQNMTSEERQRLVTIVDKLQQSTARVVVVFSPDLTLYNFFNEVLRQNFTGAVWIA   300
Sooty mangabey                 ANFLSLFLLPQITYSAISDELRDKVRFPALLRTAPSADHHIEAMVQLMLHFHWNWIIVLVSGDTYGRDNGQLLGDRLARGDICIAFQETLPTVQPNQNMTSEERQRLVTIVDKLQQSTARVVVVFSPDLTLYNFFNEVLRQNFTGAVWIA   300
Blue monkey                    ANFLSLFLLPQITYSAISDELRDKVRFPALLRTAPSADHHIEAMVQLMLYFHWNWIIVLVSGDTYGRDNGQLLGDRLARGDICIAFQETLPTVQPNQNMTSEERQRLVTIVDKLQQSTARVVVVFSPDLTLYNFFNEVLRQNFTGAVWIA   300
Sykes' monkey                  ANFLSLFLLPQITYSAISDELRDKVRFPALLRTAPSADHHIEAMVQLMLYFHWNWIIVLVSGDTYGRDNGQLLGDRLARGDICIAFQETLPTVQPNQNMTSEERQRLVTIVDKLQQSTARVVVVFSPDLTLYNFFNEVLRQNFTGAVWIA   300
Assam macaque                  ANFLSLFLLPQITYSAISDELRDKVRFPALLRTAPSADHHIEAMVQLMLHFRWNWIIVLVSGDTYGRDNGQLLGDRLARGDICIAFQETLPTVQPNQNMTSEERQRLVTIVDKLQQSTARVVVVFSPDLTLYNFFNEVLRQNFTGAVWIA   300
Stump-tailed macaque           ANFLSLFLLPQITYSAISDELRDKVRFPALLRTAPSADHHIEAMVQLMLHFRWNWIIVLVSGDTYGRDNGQLLGDRLARGDICIAFQETLPTVQPNQNMTSEERQRLVTIVDKLQQSTARVVVVFSPDLTLYNFFNEVLRQNFTGAVWIA   300
Japanese macaque               ANFLSLFLLPQITYSAISDELRDKVRFPALLRTAPSADHHIEAMVQLMLHFRWNWIIVLVSGDTYGRDNGQLLGDRLARGDICIAFQETLPTVQPNQNMTSEERQRLVTIVDKLQQSTARVVVVFSPDLTLYNFFNEVLRQNFTGAVWIA   300
Crab-eating macaque            ANFLSLFLLPQITYSAISDELRDKVRFPALLRTAPSADHHIEAMVQLMLHFRWNWIIVLVSGDTYGRDNGQLLGDRLARGDICIAFQETLPTVQPNQNMTSEERQRLVTIVDKLQQSTARVVVVFSPDLTLYNFFNEVLRQNFTGAVWIA   300
Pig-tailed macaque             ANFLSLFLLPQITYSAISDELRDKVRFPALLRTAPSADHHIEAMVQLMLHFRWNWIIVLVSGDTYGRDNGQLLGDRLARGDICIAFQETLPTVQPNQNMTSEERQRLVTIVDKLQQSTARVVVVFSPDLTLYNFFNEVLRQNFTGAVWIA   300
Rhesus macaque                 ANFLSLFLLPQITYSAISDELRDKVRFPALLRTAPSADHHIEAMVQLMLHFRWNWIIVLVSGDTYGRDNGQLLGDRLARGDICIAFQETLPTVQPNQNMTSEERQRLVTIVDKLQQSTARVVVVFSPDLTLYNFFNEVLRQNFTGAVWIA   300
Gelada baboon                  ANFLSLFLLPQITYSAISDELRDKVRFPALLRTAPSADHHIEAMVQLMLHFRWNWIIVLVSGDTYGRDNGQLLGDRLARGDICIAFQETLPTVQPNQNMTSEERQRLVTIVDKLQQSTARVVVVFSPDLTLYNFFNEVLRQNFTGAVWIA   300
Hamadryas baboon               ANFLSLFLLPQITYSAISDELRDKVRFPALLRTAPSADHHIEAMVQLMLHFRWNWIIVLVSGDTYGRDNGQLLGDRLARGDICIAFQETLPTVQPNQNMTSEERQRLVTIVDKLQQSTARVVVVFSPDLTLYNFFNEVLRQNFTGAVWIA   300
Black crested mangabey         ANFLSLFLLPQITYSAISDELRDKVRFPALLRTAPSADHHIEAMVQLMLHFRWNWIIVLVSGDTYGRDNGQLLGDRLARGDICIAFQETLPTVQPNQNMTSEERQRLVTIVDKLQQSTARVVVVFSPDLTLYDFFNEVLRQNFTGAVWIA   300
Mandrill                       ANFLSLFLLPQITYSAISDELRDKVRFPALLRTAPSADHHIEAMVQLMLHFRWNWIIVLVSGDTYGRDNGQLLGDRLARGDICIAFQETLPTVQPNQNMTSEERQRLVTIVDKLQQSTARVVVVFSPDLTLYNFFNEVLRQNFTGAVWIA   300
Drill                          ANFLSLFLLPQITYSAISDELRDKVRFPALLRTAPSADHHIEAMVQLMLHFRWNWIIVLVSGDTYGRDNGQLLGDRLARGDICIAFQETLPTVQPNQNMTSEERQRLVTIVDKLQQSTARVVVVFSPDLTLYNFFNEVLRQNFTGAVWIA   300
Red guenon                     ANFLSLFLLPQITYSAISDELRDKVRFPALLRTAPSADHHIEAMVQLMLYFHWNWIIVLVSGDTYGRDNGQLLGDRLARGDICIAFQETLPTVQPNQNMTSEERQRLVTIVDKLQQSTARVVVVFSPDLTLYNFFNEVLRQNFTGAVWIA   300
Ma's night monkey              ANFLSLFLLPQITYSAISDQLRDKQRFPALLRTAPSAKHHIEAMVQLMLHFHWNWISVLVSSDTYGRDNGQLLGDRLAGGDICIAFQETLPTLQPNQDITPEDRQRLVSIVEKLQQSTARVVVVFSPDLNLYDFFREVVRQNFTGAVWIA   300
White-tufted-ear marmoset      AHFLSLFLLPQITYSAISDQLQDKQRFPALLRTTPSAKHHIEAMVQLMLHFHWNWISVLVSSDTYGRDNGQMLGDRLAGGDICIAFQETLPTLQSNQDIMPEDHQRLVSIVEKLQQSTARVVVVFSPDLSLYNFFREVLRQNFTGAVWIA   300
Panamanian white-faced capuchin ANFLSLFLLPQITYSAISDQLRDKQRFPALLRTAPSAKHHIEAMVQLMLHFHWNWISVLVSSDTYGRDNGQLLGDRLAGSDICIAFQETLPTLQPNQDIMPEDRQRLVSIVEKLQHSTARVVVVFSPDLTLYDFFREVLRQNFTGAVWIA   300
Brown-capped capuchin          ANFLSLFLLPQITYSAISDQLRDKQRFPALLRTAPSAKHHIEAMVQLMLHFHWNWISVLVSSDTYGRDNGQLLGDRLVGSDICIAFQETLPTLQPNQDIMPEDRQRLVSIVEKLQHSTARVVVVFSPDLTLYDFFREVLRQNFTGAVWIA   300
Bolivian squirrel monkey       ANFLSLFLLPQITYSAISDQLRDKQRFPALLRTTPSAKHHIEAMVQLMLHFRWNWISVLVSSDTYGRDNGQLLGDRLAGGDICIAFQETLPTLQPNQDMMPEDRQRLVSIVEKLQQSTARVVVVFSPDLTLYDFFREVLRQNFTGAVWIA   300
Common squirrel monkey         ANFLSLFLLPQITYSAISDQLRDKQRFPALLRTTPSAKHHIEAMVQLMLHFRWNWISVLVSSDTYGRDNGQLLGDRLAGGDICIAFQETLPTLQPNQDMMPEDRQRLVSIVEKLQQSTARVVVVFSPDLTLYDFFREVLRQNFTGAVWIA   300
Coquerel's sifaka              ANFLSLFLLPQITYSAISDQLRNKHRFPALLRTMPSADHHVEAIAQLMLHFRWNWIVVLVSSDDYGRDNGQRLSERLTGRDICIAFQESLPAPQPNQVATPEEHSRLGAIVDKLQQSTARVVVVFSPDLALHNFFREVLRRNFTGVVWIA   300
Gray mouse lemur               ANFLSLFLLPQITYSAISDQLRDKDRFPALLRTMPSADHHVEAIAQLLLHFRWNWIVVLVSSDDYGRDNGQRLSERLTGRDICIAFQESLPTPQPNQVMTTEEHRRLRAIVDKLQQTSARVVVVFSPDLALHNFFHEVVRWNFTGAVWIA   300
Ring-tailed lemur              ANFLSLFLLPQITYSAISDQLRDKQRYPALLRTMPSADHHVEAIAQLMLYFRWNWIVVLVSSDDYGRDNGQRLSERLTGHDICIAFQESLPVPQPNQVVTPEEHSRLGAIVEKLQQSSARVVVVFSPDLALHNFFLEVMRRNYTGAVWIA   300
```

```
                                ****:******..  *:: ****.:* *.*** .*.***    :* .     . :  ******* *:* * .:: :* *****:**.** ***.******.:*.*.   * *  ****:**:*:*****:**.::: *: .**:.: *:.
Human                           SESWAIDPVLHNLTELRHLGTFLGITIQSVPIPGFSEFREWGPQAGPPPLSRTSQSYTCNQECDNCLNATLSFNTILRLSGERVVYSVYSAVYAVAHALHSLLGCDKSTCTKRVVYPWQLLEEIWKVNFTLLDHQIFFDPQGDVALHLEI   450
Western lowland gorilla         SESWAIDPVLHNLTELRHLGTFLGITIQSVPIPGFSEFREWSPQAGPPPLSRTSQSYTCNQECDNCLNATLSFNTILRLSGERVVYSVYSAVYAVAHALHSLLGCDNSTCTKRVVYPWQLLEEIWKVNFTLLDHQIFFDPQGDVALHLEI   450
Sumatran orangutan              SESWAIDPVLHNLTELRHTGTFLGITIQSVPIPGFSEFRERDSQAGPPPLGKTSQRSTCNQECDNCLNATLSFNTILRLSGERVVYSVYSAVYAVAHALHSLLGCDHSTCTKRVVYPWQLLEEIWKVNFTLLDHQIFFDPQGDMALHLEI   450
Bornean orangutan               SESWAIDPVLHNLTELRHTGTFLGITIQSVPIPGFSEFRERDSQAGPPPLGKTSQRSTCNQECDNCLNATLSFNTILRLSGERVVYSVYSAVYAVAHALHSLLGCDHSTCTKRVVYPWQLLEEIWKVNFTLLDHQIFFDPQGDMALHLEI   450
Pygmy chimpanzee                SESWAIDPVLHNLTELRHLGTFLGITIQSVPIPGFSEFREWGPQAGPPPLSRTSQSYTCNQECDNCLNATLSFNTILRLSGERVVYSVYSAVYAVAHALHSLLGCDNSTCTKRVVYPWQLLEEIWKVNFTLLDHQIFFDPQGDVALHLEI   450
Common chimpanzee                SESWAIDPVLHNLTELRHLGTFLGITIQSVPIPGFSEFREWGPQAGPPPLSRTSQSYTCNQECDNCLNATLSFNTILRLSGERVVYSVYSAVYAVAHALHSLLGCDNSTCTKRVVYPWQLLEEIWKVNFTLLDHQIFFDPQGDVALHLEI   450
Northern white-cheeked gibbon   SESWAIDPVLHNLTELRHMGTFLGITIQSVPIPGFSEFRERGPQAGPPPLSRSSQRSTCNQECDNCLNATLSFNTVLRLSGERVVYSVYSAVYAVAHALHSLLNCDHSTCTKREVYPWQLLEEVWKVNFTLLDHQIFFDSQGDLALHLEI   450
Northern buffed-cheeked gibbon  SESWAIDPVLHNLTELRHMGTFLGITIQSVPIPGFSEFRERGPQAGPPPLSRSSQRSTCNQECDNCLNATLSFNTVLRLSGERVVYSVYSAVYAVAHALHSLLNCDHSTCTKREVYPWQLLEEVWKVNFTLLDRQIFFDSQGDLALHLEI   450
Western hoolock gibbon          SESWAIDPVLHNLTELRHMGTFLGITIQSVPIPGFSEFRERGPQAGPPPLSRSSQRSTCNQECDNCLNATLSFNTVLRLSGERVVYSVYSAVYAVAHALHSLLGCDHSTCTKREVYPWQLLEEVWKVNFTLLDHQIFFDSQGDLALHLEI   450
Hylobates muelleri abbotti      SESWAIDPVLHNLTELRHMGTFLGITIQSVPIPGFSEFRERGQQAGPPPLSRTSQRSTCNQECDDCLNATLSFNTVLRLSGERVVYSVYSAVYAVAHALHSLLDCDHSTCTKREVYPWQLLEEVWKVNFTLLDHQIFFDSQGDLALHLEI   450
Agile gibbon                    SESWAIDPVLHNLTELRHMGTFLGITIQSVPIPGFSEFRERSQQAGPPPLSRTSQRSTCNQECDDCLNATLSFNTVLRLSGERVVYSVYSAVYAVAHALHSLLDCDHSTCTKREVYPWQLLEEVWKVNFTLLDHQIFFDSQGDLALHLEI   450
Common gibbon                   SESWAIDPVLHNLTELRHMGTFLGITIQSVPIPGFSEFRERGQQAGPPPLSRTSQRSTCNQECDDCLNATLSFNTVLRLSGERVVYSVYSAVYAVAHALHSLLDCDHSTCTKREVYPWQLLEEVWKVNFTLLDHQIFFDSQGDLALHLEI   450
Pileated gibbon                 SESWAIDPVLHNLTELRHMGTFLGITIQSVPIPGFSEFRERGQQAGPPPLSRTSQRSTCNQECDDCLNATLSFNTVLRLSGERVVYSVYSAVYAVAHALHSLLDCDHSTCTKREVYPWQLLEEVWKVNFTLLDHQIFFDSQGDLALHLEF   450
Siamang                         SESWAIDPVLHNLTELRHMGTFLGITIQSVPIQGFSEFRERGPQAGPPPLSRSSQRSTCNQECDNCLNATLSFNTVLRLSGERVVYSVYSAVYAVAHALHSLLGCDHSTCTKREVYPWQLLEEVWKVNFTLLDHQIFFDSQGDLALHLEI   450
Green monkey                    SESWAIDPVLHNLTELRHMGTFLGITIQSVPIPGFSEFRVRDPQAGPPPLSRTSQRSTCNQECDSCLNGTLSFDNVLRLSGERVVYSVYSAVYAVAHALHSLLGCDHGTCTKKEVYPWQLLKEIWKVNFTLLDHQISFDPQGDMALHLEI   450
Golden-bellied mangabey         SESWAIDPVLHNLTELRHMGTFLGITIQSVPIPGFSEFRVRDPQAGPPPLSRTSQRSTCNQECDSCLNGTLSFNNVLRLSGERVVYSVYAAVYAVAHALHSLLGCDHGTCTKTEVYPWQLLKEIWKVNFTLLDHEISFDPQGDMALHLEI   450
Sooty mangabey                  SESWAIDPVLHNLTELRHMGTFLGITIQSVPIPGFSEFRVRDPQAGPPPLSRTSQRSTCNQECDSCLNGTLSFNNVLRLSGERVVYSVYAAVYAVAHALHSLLGCDHGTCTKKEVYPWQLLKEIWKVNFTLLDHEVSFDPQGDMALHLEI   450
Blue monkey                     SESWAIDPVLHNLTELRHMGTFLGITIQSVPIPGFSEFRVRDPQAGPPPLSRTSQRSTCNQECDSCLNGTLSFDNVLRLSGERVVYSVYSAVYAVAHALHSLLGCDHGACIKKEVYPWKLLKEIWKVNFTLLDHQISFDPQGDMALHLEI   450
Sykes' monkey                   SESWAIDPVLHNLTELRHMGTFLGITIQSVPIPGFSEFRVRDPQAGPPPLSRTSQRSTCNQECDSCLNGTLSFDNVLRLSGERVVYSVYSAVYAVAHALHSLLGCDHGACIKKEVYPWKLLKEIWKVNFTLLDHQISFDPQGDMALHLEI   450
Assam macaque                   SESWAIDPVLHNLTELRHMGTFLGITIQSVPIPGFSEFRVRDPQAGPPPLSRTSQRSTCNQECDSCLNGTLSFNNVLRLSGERVVYSVYSAVYAVAHALHSLLGCDHGTCTKREVYPWQLLKEIWKVNFTLLDHEISFDPQGDMALHLEI   450
Stump-tailed macaque            SESWAIDPVLHNLTELRHMGTFLGITIQSVPIPGFSEFRVRDPQAGPPPLSRTSQRSTCNQECDSCLNGTLSFNNVLRLSGERVVYSVYSAVYAVAHALHSLLGCDHGTCTKREVYPWQLLKEIWKVNFTLLDHEISFDPQGDMALHLEI   450
Japanese macaque                SESWAIDPVLHNLTELRHMGTFLGITIQSVPIPGFSEFRVRDPQAGPPPLSRTSQRSTCNQECDSCLNGTLSFNNVLRLSGERVVYSVYSAVYAVAHALHSLLGCDHGTCTKREVYPWQLLKEIWKVNFTLLDHEISFDPQGDMALHLEI   450
Crab-eating macaque             SESWAIDPVLHNLTELRHMGTFLGITIQSVPIPGFSEFRVRDPQAGPPPLSRTSQRSTCNQECDSCLNGTLSFNNVLRLSGERVVYSVYSAVYAVAHALHSLLGCDHGTCTKREVYPWQLLKEIWKVNFTLLDHEISFDPQGDMALHLEI   450
Pig-tailed macaque              SESWAIDPVLHNLTELRHMGTFLGITIQSVPIPGFSEFRVRDPQAGPPPLSRTSQRSTCNQECDSCLNGTLSFNNVLRLSGERVVYSVYSAVYAVAHALHSFLGCDHGTCTKREVYPWQLLKEIWKVNFTLLDHEISFDPQGDMALHLEI   450
Rhesus macaque                  SESWAIDPVLHNLTELRHMGTFLGITIQSVPIPGFSEFRVRDPQAGPPPLSRTSQRSTCNQECDSCLNGTLSFNNVLRLSGERVVYSVYSAVYAVAHALHSLLGCDHGTCTKREVYPWQLLKEIWKVNFTLLDHEISFDPQGDMALHLEI   450
Gelada baboon                   SESWAIDPVLHNLTELRHVGTFLGITIQSVPIPGFSEFRVRDPQAGPPPLSRTSQRSTCNQECDSCLNGTLSFNNVLRLSGERVVYSVYSAVYAVAHALHSLLGCDHGTCTKTEVYPWQLLKEIWKVNFTLLDHEISFDPQGDMALHLEI   450
Hamadryas baboon                SESWAIDPVLHNLTELRHMGTFLGITIQSVPIPGFSEFRVRDPQAGPPPLSRTSQRSTCNQECDSCLNGTLSFNNVLRLSGERVVYSVYSAVYAVAHALHSLLGCDHGTCTKTEVYPWQLLKEIWKVNFTLLDHQISFDPQGDMALHLEI   450
Black crested mangabey          SESWAIDPVLHNLTELRHMGTFLGITIQSVPIPGFSEFRVRDPQAGPPPLSRTSQRSTCNQECDSCLNGTLSFNNVLRLSGERVVYSVYSAVYAVAHALHSLLGCDHGTCAKTEVYPWQLLKEIWKVNFTLLDHQIAFDPQGDMALHLEI   450
Mandrill                        SESWAIDPVLHNLTELRHMGTFLGITIQSVPIPGFSEFRVRDPQAGPPPLSRTSQRSTCNQECDSCLNGTLSFNNVLRLSGERVVYSVYSAVYAVAHALHSLLGCDYGTCAKKEVYPWQLLKEIWKVNFTLLDHEISFDPQGDMALHLEI   450
Drill                           SESWAIDPVLHNLTELRHMGTFLGITIQSVPIPGFSEFRVRDPQAGPPPLSRTSQRSTCNQECDSCLNGTLSFNNVLRLSGERVVYSVYCAVYAVAHALHSLLGCDYGTCAKKEVYPWQLLKEIWKVNFTLLDHEISFDPQGDMALHLEI   450
Red guenon                      SESWAIDPVLHNLTELRHMGTFLGITIQSVPIPGFSEFRVRDPQAGPPPLSRSSQRSTCNQECDSCLNGTLSFDNVLRLSGERVVYSVYSAVYAVAHALHSLLGCDHGTCTKKEVYPWQLLKEIWKVNFTLLDHQISFDPQGDMALHLEI   450
Ma's night monkey               SESWAIDPVLHNVTGLHRTGTFLGITIQNVPIPGFSEFRVRGPQAGP-----TNQRSTCNQECDTCLNSTLSFNTILRLSGERIVYSVYSAVYAVAHALHSLLGCDHSACTKRVVYPWQLLEEIWKVNFTLLDHQIFFDPQGDVALHLEI   445
White-tufted-ear marmoset       SESWAIDPVLHNLTGLHRTGTFLGITIQNVPIPGFSEFRVRGPQAEP-----TNQRSTCNQECDTCLNSTLSFNTVLRLSGERIVYSVYSAVYAVAHALHSLLSCDHSTCTKRVVYPWQLLEEIWKVNFTLLDHQIFFDPQGDVALHLEI   445
Panamanian white-faced capuchin SESWSIDPVLHNLTGLHRTGTFLGITIQNVPIPGFSEFRVRGPQAEP-----TNQRSTCNQECDTCLNSTLSFNSILRLSGERIVYSVYSAVYVVAHALHSLLGCDHSACTKRVVYPWQLLEEIWKVNFTLLDHQISFDPHGDVALHLEI   445
Brown-capped capuchin           SESWSIDPVLHNLMGLHRTGTFLGITIQNVPIPGFSEFRVRGPQAEP-----TNQRSTCNQECDTCLNSTLSFNSILRLSGERIVYSVYSAVYAVAHALHSLLGCDHSACTKRVVYPWQLLEEIWKVNFTLLDHQISFDPQGDVALHLEI   445
Bolivian squirrel monkey        SESWAIDPVLHNLTGLHRTGTFLGITLQNVPIPGFNEFRVRGPQAGP-----THQRSTCNQECDTCLNSTLSFNTILRLSGERVVYSVYSAVYAVAHALHSLLGCDHSACTKRVVYPWQLLEEIWKVNFTLLDHQISFDPQGDVALHLEI   445
Common squirrel monkey          SESWAIDPVLHNLTGLHRTGTFLGITLQNVPIPGFNEFRVRGPQAGP-----THQRSTCNQECDTCLNSTLSFNTILRLSGERVVYSVYSAVYAVAHALHSLLGCDHSACTKRGVYPWQLLEEIWKVNFSLLDHQIFFDPQGDVALHLEI   445
Coquerel's sifaka               SESWAIDPVLHSFTELRHAGTFLAVTTQSVPIPGFSEFRVRRWQAGPPS-GRNSQGSTCNQECDECFNTTKSFNTILTLSGERVVYGVYSAVYAVAHALHSLLGCGETNCTKEVVYPWQLLKEIWKVNFTLLGHQIIFDHEGDMAMPLDI   449
Gray mouse lemur                SESWAIDPVLHNLTELRHTGTFLGVTIQNVPIPGFSEFRMRRWQAGSPP-SRNGRGATCNQECDKCFNTTETFNEVLTLSGERIVYSVYSAVYAVAHALHSLLGCGETGCTKEVVYPWQLLKEIWKVNFTLLGHQIAFDKDGDMPMPLEV   449
Ring-tailed lemur               SESWAIDPVLHNLIELRHTGTFLGVTTQNVPIPSFSEFRVRSRRAGPPP-RRSSQASTCNQECDECFNTTENYNNVLTLSGERVVYSVYTAVYAVAHALHNLLGCSETGCTKKVVYPWQLLKELWKVNFTLLGHQIKFNQEGDMPIPLEV   449
```

```
                                  :****. .*. ***:*** * * :*. *  ***** ** :*:****: *:.**:**.:* * ******** ******:: :*::**.* ..:**::. :***:*:* *******.** **:******:*****: :****:.**:*****
Human                             VQWQWDRSQNPFQSVASYYPLQRQLKNIQDISWHTINNTIPMSMCSKRCQSGQKKPVGIHVCCFECIDCLPGTFLNHTEDEYECQACPNNEWSYQSETSCFKRQLVFLEWHEAPTIAVALLAALGFLSTLAILVIFWRHFQTPIVRSAG   600
Western lowland gorilla           VQWQWDRSQNPFQSVASYYPLQRQLKHIQDISWHTINNTIPVSMCSKRCQSGQKKPVGIHVCCFECIDCLPGTFLNHTEDEYECQACPNNEWSYQSETSCFKRQLVFLEWHEAPTIAVALLAALGFLSTLAILVIFWRHFQTPIVRSAG   600
Sumatran orangutan                VQWQWDRSQNPFQSVASYHPLQRQLKNIQDISWHTINNTIPVSMCSKRCQSGQKKPVGIHVCCFECIDCLPGTFLNHTEDEYECQACPSNEWSYQSETSCFKRQLAFLEWHEAPTIAVALLAALGFLSTLAILVIFWRHFQTPMVRSAG   600
Bornean orangutan                 VQWQWDRSQNPFQSVASYHPLQRQLKNIQDISWHTINNTIPVSMCSKRCQSGQKKPVGIHVCCFECIDCLPGTFLNHTEDEYECQACPSNEWSYQSETSCFKRQLAFLEWHEAPTIAVALLAALGFLSTLAILVIFWRHFQTPMVRSAG   600
Pygmy chimpanzee                  VQWQWDRSQNPFQSVASYYPLQRQLKNIQDISWHTINNTIPVSMCSKRCQSGQKKPVGIHVCCFECIDCLPGTFLNHTEDEYECQACPNNEWSYQSETSCFKRQLVFLEWHEAPTIAVALLAALGFLSTLAILVIFWRHFQTPIVRSAG   600
Common chimpanzee                 VQWQWDRSQNPFQSVASYYPLQRQLKNIQDISWHTINNTIPVSMCSKRCQSGQKKPVGIHVCCFECIDCLPGTFLNHTEDEYECQACPNNEWSYQSETSCFKRQLVFLEWHEAPTIAVALLAALGFLSTLAILVIFWRHFQTPIVRSAG   600
Northern white-cheeked gibbon     VQWQWDQSQNPFQSVASYYPLQRQLKNIQDISWHTINNTIPVSMCSKRCQSGQKKPVGIHVCCFECIDCLPGTFLNHTEDEYECQACPNNEWSHQSETSCFKRQLVFLEWHEAPTIAVALLAALGFLSTLAILVVFWRHFQTPMVRSAG   600
Northern buffed-cheeked gibbon    VQWQWDQSQNPFQSVASYYPLQRQLKNIQDISWHTINNTIPVSMCSKRCQSGQKKPVGIHVCCFECIDCLPGTFLNHTEDEYECQACPNNEWSHQSETSCFKRQLVFLEWHEAPTIAVALLAALGFLSTLAILVVFWRHFQTPMVRSAG   600
Western hoolock gibbon            VQWQWDKSQNPFQSVASYYPLQRQLKNIQDISWHTINNTIPVSMCSKRCQSGQKKPVGIHVCCFECIDCLPGTFLNHTEDEYECQACPSNEWSHQSETSCFKRQLVFLEWHEAPTIAVALLAALGFLSTLAILVVFWRHFQTPMVRSAG   600
Hylobates muelleri abbotti        VQWQWDQSQNPFQSVASYYPLQRQLKNIQDISWHTINNTIPVSMCSKRCQSGQKKPVGIHVCCFECIDCLPGTFLNHTEDEYECQACPNNEWSHQSETSCFKRQLVFLEWHEAPTIAVALLAALGFLSTLAILVVFWRHFQTPMVRSAG   600
Agile gibbon                      VQWQWDQSQNPFQSVASYYPLQRQLKNIQDISWHTINNTIPVSMCSKRCQSGQKKPVGIHVCCFECIDCLPGTFLNHTEDEYECQACPNNEWSHQSETSCFKRQLVFLEWHEAPTIAVALLAALGFLSTLAILVVFWRHFQTPMVRSAG   600
Common gibbon                     VQWQWDQSQNPFQSVASYYPLQRQLKNIQDISWHTINNTIPVSMCSKRCQSGQKKPVGIHVCCFECIDCLPGTFLNHTEDEYECQACPNNEWSHQSETSCFKRQLVFLEWHEAPTIAVALLAALGFLSTLAILVVFWRHFQTPMVRSAG   600
Pileated gibbon                   VQWQWDQSQNPFQSVASYYPLQRQLKNIQDISWHTINNTIPVSMCSKRCQSGQKKPVGIHVCCFECIDCLPGTFLNHTEDEYECQACPNNEWSHQSETSCFKRQLVFLEWHEAPTIAVALLAALGFLSTLAILVVFWRHFQTPMVRSAG   600
Siamang                           VQWQWDQSQNPFQSVASYYPLQRQLKNIQDISWHTINNTIPVSMCSKRCQSGQKKPVGIHVCCFECIDCLPGTFLNHTEDEYECQACPSNEWSHQSETSCFKRQLVFLEWHEAPTIAVALLAALGFLSTLAILVVFWRHFQTPMVRSAG   600
Green monkey                      VQWQWGLSQNPFQSVASYYPLQRQLKKIQDISWHTINNTIPVSMCSKRCQSGQKKPVGIHICCFECIDCLPGTFLNQTEDEYECQACPSNEWSHQSEASCFKRRLAFLEWHEAPTIVVALLAALGFLSTLAILVIFWRHFQTPMVRSAG   600
Golden-bellied mangabey           VQWQWDLSQNPFQSVASYYPLQRQLKTIQDISWHTINNTIPVSMCSKRCQSGQKKPVGIHICCFECIDCLPGTFLNQTEDEYECQACPSNEWSHQSEASCFKRRLAFLEWHEAPTIVVALLAALGFLSTLAILVIFWRHFQTPMVRSAG   600
Sooty mangabey                    VQWQWDLSQNPFQSVASYYPLQRQLKTIQDISWHTINNTIPVSMCSKRCQSGQKKPVGIHICCFECIDCLPGTFLNQTEDEYECQACPSNEWSHQSEASCFKRRLAFLEWHEAPTIVVALLAALGFLSTLAILVIFWRHFQTPMVRSAG   600
Blue monkey                       VQWQWGLSQNPFQSVASYYPLQRQLKIIQDISWHTINNTIPVSMCSKRCQSGQKKPVGIHICCFECIDCLPGTFLNQTEDEYECQACPSNEWSHQSEASCFKRRLAFLEWHEAPTIVVALLAALGFLSTLAILVIFWRHFQTPMVRSAG   600
Sykes' monkey                     VQWQWGLSQNPFQSVASYYPLQRQLKIIQDISWHTINNTIPVSMCSKRCQSGQKKPVGIHICCFECIDCLPGTFLNQTEDEYECQACPSNEWSHQSEASCFKRRLAFLEWHEAPTIVVALLAALGFLSTLAILVIFWRHFQTPMVRSAG   600
Assam macaque                     VQWQWGLSQNPFQSVASYYPLQRQLKKIQDISWHTINNTIPVSMCSKRCQSGQKKPVGIHICCFECIDCLPGTFLNQTEDEYECQACPSNEWSHQSEASCFKRRLAFLEWHEAPTIVVALLAALGFLSTLAILVIFWRHFQTPMVRSAG   600
Stump-tailed macaque              VQWQWGLSQNPFQSVASYYPLQRQLKKIQDISWHTINNTIPVSMCSKRCQSGQKKPVGIHICCFECIDCLPGTFLNQTEDEFECQACPSNEWSHQSEASCFKRRLAFLEWHEAPTIVVALLAALGFLSTLAILVIFWRHFQTPMVRSAG   600
Japanese macaque                  VQWQWGLSQNPFQSVASYYPLQRQLKKIQDISWHTINNTIPVSMCSKRCQSGQKKPVGIHICCFECIDCLPGTFLNQTEDEYECQACPSNEWSHQSEASCFKRRLAFLEWHEAPTIVVALLAALGFLSTLAILVIFWRHFQTPMVRSAG   600
Crab-eating macaque               VQWQWGLSQNPFQSVASYYPLQRQLKKIQDISWHTINNTIPVSMCSKRCQSGQKKPVGIHICCFECIDCLPGTFLNQTEDEYECQACPSNEWSHQSEASCFKRRLAFLEWHEAPTIVVALLAALGFLSTLAILVIFWRHFQTPMVRSAG   600
Pig-tailed macaque                VQWQWGLSQNPFQSVASYYPLQRQLKKIQDISWHTINNTIPVSMCSKRCQSGQKKPVGIHICCFECIDCLPGTFLNQTEDEYECQACPSNEWSHQSEASCFKRRLAFLEWHEAPTIVVALLAALGFLSTLAILVIFWRHFQTPMVRSAG   600
Rhesus macaque                    VQWQWGLSQNPFQSVASYYPLQRQLKKIQDISWHTINNTIPVSMCSKRCQSGQKKPVGIHICCFECIDCLPGTFLNQTEDEFECQACPSNEWSHQSEASCFKRRLAFLEWHEAPTIVVALLAALGFLSTLAILVIFWRHFQTPMVRSAG   600
Gelada baboon                     VQWQWDLSQNPFQSVASYYPLQRQLKKIQDISWHTINNTIPVSMCSKRCQSGQKKPVGIHICCFECIDCLPGTFLNQTEDEYECQACPSNEWSHQSEASCFKRRLAFLEWHEAPTIVVALLAALGFLSTLAILVIFWRHFQTPMVRSAG   600
Hamadryas baboon                  VQWQWDLSQNPFQSVASYYPLQRQLKTIQDISWHTINNTIPVSMCSKRCQSGQKKPVGIHICCFECIDCLPGTFLNQTEDEYECQACPSNEWSHQSEASCFKRRLAFLEWHEAPTIVVALLAALGFLSTLAILVIFWRHFQTPMVRSAG   600
Black crested mangabey            VQWQWDLSQNPFQSVASYYPLQRQLKTIQDISWHTINNTIPVSMCSKRCQSGQKKPVGIHICCFECIDCLPGTFLNQTEDEYECQACPSNEWSHQSEASCFKRRLAFLEWHEAPTIVVALLAALGFLSTLAILVIFWRHFQTPMVRSAG   600
Mandrill                          VQWQWDLSQNPFQSVASYYPLQRQLKTIQDISWHTINNTIPVSMCSKRCQSGQKKPVGIHICCFECIDCLPGTFLNQTEDEYECQACPSNEWSHQSEASCFKRRLAFLEWHEAPTIVVALLAALGFLSTLAILVIFWRHFQTPMVRSAG   600
Drill                             VQWQWDLSQNPFQSVASYYPLQRQLKTIQDISWHTINNTIPVSMCSKRCQSGQKKPVGIHICCFECIDCLPGTFLNQTEDEYECQACPSNEWSHQSEASCFKRRLAFLEWHEAPTIVVALLAALGFLSTLAILVIFWRHFQTPMVRSAG   600
Red guenon                        VQWQWGLSQNPFQSVASYYPLQRQLKKIQDISWHTINNTIPVSMCSKRCQSGQKKPVGIHICCFECIDCLPGTFLNQTEDEYECQACPSNEWSHQSEASCFKRRLAFLEWHEAPTIVVALLAALGFLSTLAILVIFWRHFQTPMVRSAG   600
Ma's night monkey                 VQWQWDLSQNPFQSIASYSPLQGQLKHIQDISWHTVNNTIPVSMCSKRCQSGQKKPVGIHTCCFECIDCLPGTFLNQTANEYDCQTCPSNEWSHQSETSCFKRRLSFLEWHEAPTIAVALLAALGFLSTLAILVIFWRHFQTPMVRSAG   595
White-tufted-ear marmoset         VQWQWDLSQNPFQSIASYNPLQGRLKHIQDISWHTINNTIPVSMCSKRCQSGQKKPVGIHTCCFECIDCLPGTFLNQTANEYDCQACLSNEWSHQSETSCFKRRLSFLEWHEAPTIAVALLAALGFLSTLAILVIFWRHFHTPMVRSAG   595
Panamanian white-faced capuchin   VQWQWDLNQNLFQSVASYSPLQGHLKDIQDISWHTVNNTIPVSMCSKRCQSGQKKPMGIHTCCFECIDCLPGTFLNQTANEYDCQACPSNEWSHQSETSCFKRRLSFLEWHEAATIAVALLAALGFLSTLAILVIFWRHFQTPMVRSAG   595
Brown-capped capuchin             VQWQWDLNQNLFQSVASYSPLQGHLKDIQDISWHTVNNTIPVSMCSKRCQSGQKKPMGIHTCCFECIDCLPGTFLNQTANEYDCQACPSNEWSHQSETSCFKRRLSFLEWHEAATIAVALLAALGFLSTLAILVIFWRHFQTPMVRSAG   595
Bolivian squirrel monkey          VQWQWDLSQNFFQSVASYSPLQGHLKDIQDISWHTVNNTIPVSMCSKRCQSGQKKPVGIHTCCFECIDCPPGTFLNQTANEYDCQACPSNEWSHQSETSCFKRRLSFLEWHEAATIAVALLAALGFLSTLAILVIFWRHFETPMVRSAG   595
Common squirrel monkey            VQWQWDLSQNPFQSVASYQPLQGHLKDIQDISWHTVNNTIPVSMCSKRCQSGQKKPVGIHTCCFECIDCPPGTFLNQTANEYDCQACPSNEWSHQSETSCFKRRLSFLEWHEAATIAVALLAALGFLSTLAILVIFWRHFETPMVRSAG   595
Coquerel's sifaka                 IQWQWNMSQSPFQSIASYYPAQRQLKNISYISWHTPNNTVPVSMCSKSCQPGQRKKPVGTHSCCFECIDCLPGTFLNQTADEYDCQSCPNNKWSHRSGTSCFRRRLAFLEWHEAPTIIVAMLAALGFFSTLAIMVIFWRHFQTPMVRSAG   599
Gray mouse lemur                  VQWQWDLSQSPFQSVASYYPLQRQLTNIGHISWHTPNNMVPVSMCSKHCQPGQRKKPVGIHPCCFECIDCLPGTFLNQTADEYDCQPCPSNKWSHRNGTSCFKRRLAFLEWHEAPTIIVAMLAALGFLSTLAILAIFWRHLQTPMVRSAG   599
Ring-tailed lemur                 IQWQWGMNQNPFQSVASYYPTQRQLTNIGSISWHTPNNTVPLSMCSRSCHPGQRKKSVGIHPCCFECIDCLPGTFLNQSADEYDCQPCPSTKWSHRNATSCFKRRLAFLEWHEAPTIFVALLAALGFLSTLAILLIFWRHFQTPVVRSAG   599
```

```
                                   ********: ***:* *****:* *..:**: * :::*:***:*****::*****:  *****::**** *****:*. **: . * **:  **   *.*. .**:* *.***:: *:****.:*. *:..:.*** ***.**.** :*:
Human                              GPMCFLMLTLLLVAYMVVPVYVGPPKVSTCLCRQALFPLCFTICISCIAVRSFQIVCAFKMASRFPRAYSYWVRYQGPYVSMAFITVLKMVIVVIGMLATGLSPTTRTDPDDPKITIVSCNPNYRNSLLFNTSLDLLLSVVGFSFAYMGK    750
Western lowland gorilla            GPMCFLMLTLLLVAYMVVPVYVGPPKVSTCLCRQALFPLCFTICISCIAVRSFQIVCAFKMASRFPRAYSYWVRYQGPYVSMAFITVLKMVIVVIGMLATGLSPTTRTDPDDPKITIVSCNPNYRNSLLFNTSLDLLLSVVGFSFAYMGK    750
Sumatran orangutan                 GPMCFLMLTLLLVAYMVVPVYVGPPKVSTCLCRQALFPLCFTICISCIAVRSFQIICAFKMASRFPRAYSYWVRYQGPYVSMAFITVLKMVIVVIGMLATGLSPTTRTDPDDPKIMIVSCNPNYRNSLLFNTSLDLLLSVVGFSFAYMGK    750
Bornean orangutan                  GPMCFLMLTLLLVAYMVVPVYVGPPKVSTCLCRQALFPLCFTICISCIAVRSFQIICAFKMASRFPRAYSYWVRYQGPYVSMAFITVLKMVIVVIGMLATGLNPTTRTDPDDPKIMIVSCNPNYRNSLLFNTSLDLLLSVVGFSFANMGK    750
Pygmy chimpanzee                   GPMCFLMLTLLLVAYMVVPVYVGPPKVSTCLCRQALFPLCFTICISCIAVRSFQIVCAFKMASRFPRAYSYWVRYQGPYVSMTFITVLKMVIVVIGMLATGLSPTTRTDPDDPKITIVSCNPNYRNSLLFNTSLDLLLSVVGFSFAYMGK    750
Common chimpanzee                  GPMCFLMLTLLLVAYMVVPVYVGPPKVSTCLCRQALFPLCFTICISCIAVRSFQIVCAFKMASRFPRAYSYWVRYQGPYVSMAFITVLKMVIVVIGMLATGLSPTTRTDPDDPKITIVSCNPNYRNSLLFNTSLDLLLSVVGFSFAYMGK    750
Northern white-cheeked gibbon      GPMCFLMLTLLLVAYMVVPVYMGPPKVSTCLCRQALFPLCFTICISCIAVRSFQIVCAFKMASRFPRAYSYWVRYQGPYVSMAFITVLKMVIVVIGMLATGLSPTTRTDPDDPKITIVSCNPNYRNSLLFNTSLDLLLSVVGFSFAYMGR    750
Northern buffed-cheeked gibbon     GPMCFLMLTLLLVAYMVVPVYMGPPKVSTCLCRQALFPLCFTICISCIAVRSFQIVCAFKMASRFPRAYSYWVRYQGPYVSMAFITVLKMVIVVIGMLATGLSPTTRTDPDDPKITIVSCNPNYRNSLLFNTSLDLLLSVVGFSFAYMGR    750
Western hoolock gibbon             GPMCFLMLTLLLVAYMVVPVYMGPPKVSTCLCRQALFPLCFTICISCIAVRSFQIVCAFKMASRFPRAYSYWVRYQGPYVSMAFITVLKMVIVVIGMLATGLSPTTRTDPDDPKITIVSCNPNYRNSLLFNTSLDLLLSVVGFSFAYMGR    750
Hylobates muelleri abbotti         GPMCFLMLTLLLVAYMVVPVYMGPPKVSTCLCRQALFPLCFTICISCITVRSFQIVCAFKMASRFPRAYSYWVRYQGPYVSMAFITVLKMVIVVIGMLATGLSPTTRTDPDDPKITIVSCNPNYRNSLLFNTSLDLLLSVVGFSFAYMGR    750
Agile gibbon                       GPMCFLMLTLLLVAYMVVPVYMGPPKVSTCLCRQALFPLCFTICISCITVRSFQIVCAFKMASRFPRAYSYWVRYQGPYVSMAFITVLKMVIVVIGMLATGLSPTTRTDPDDPKITIVSCNPNYRNSLLFNTSLDLLLSVVGFSFAYMGR    750
Common gibbon                      GPMCFLMLTLLLVAYMVVPVYMGPPKVSTCLCRQALFPLCFTICISCITVRSFQIVCAFKMASRFPRAYSYWVRYQGPYVSMAFITVLKMVIVVIGMLATGLSPTTRTDPDDPKITIVSCNPNYRNSLLFNTSLDLLLSVVGFSFAYMGR    750
Pileated gibbon                    GPMCFLMLTLLLVAYMVVPVYMGPPKVSTCLCRQALFPLCFTICISCITVRSFQIVCAFKMASRFPRAYSYWVRYQGPYVSMAFITVLKMVIVVIGMLATGLSPTTRTDPDDPKITIVSCNPNYRNSLLFNTSLDLLLSVVGFSFAYMGR    750
Siamang                            GPMCFLMLTLLLVAYMVVPVYMGPPKVSTCLCRQALYPLCFTICISCIAVRSFQIVCAFKMASRFPRAYSYWVRYQGPYVSMAFITVLKMVIVVIGMLATGLSPTTRTDPDDPKITIVSCNPNYRNSLLFNTSLDLLLSVVGFSFAYMGR    750
Green monkey                       GPMCFLMLTLLLVAYMVVPVYVGPPKVSTCFCRQALFPLCFTICISCIAVRSFQIVCVFKMASRFPRAYSYWVRYQGPYVSMAFITVLKMVTVVIGMLATGLNPTTRIDPDDPKIMIVSCNPNYRNSLFFNTSLDLLLSVVGFSFAYMGK    750
Golden-bellied mangabey            GPMCFLMLTLLLVAYMVVPVYVGPPKVSTCFCRQALFPLCFTICISCIAVRSFQIVCVFKMASRFPRAYSYWVRYQGPYVSMAFITVLKMVTVVIGMLATGLNPTTRIDPDDPKIMIVSCNPNYRNSLFFNTSLDLLLSVVGFSFAYMGK    750
Sooty mangabey                     GPMCFLMLTLLLVAYMVVPVYVGPPKVSTCFCRQALFPLCFTICISCIAVRSFQIVCVFKMASRFPRAYSYWVRYQGPYVSMAFITVLKMVTVVIGMLATGLNPTTRIDPDDPKIMIVSCNPNYRNSLFFNTSLDLLLSVVGFSFAYMGK    750
Blue monkey                        GPMCFLMLTLLLVAYMVVPVYVGPPKVSTCFCRQALFPLCFTICISCIAVRSFQIVCVFKMASRFPRAYSYWVRYQGPYVSMAFITVLKMVTVVIGMLATGLNPTTRIDPDDPKIMIVSCNPNYRNSLFFNTSLDLLLSVVGFSFAYMGK    750
Sykes' monkey                      GPMCFLMLTLLLVAYMVVPVYVGPPKVSTCFCRQALFPLCFTICISCIAVRSFQIVCVFKMASRFPRAYSYWVRYQGPYVSMAFITVLKMVTVVIGMLATGLNPTTRIDPDDPKIMIVSCNPNYRNSLFFNTSLDLLLSVVGFSFAYMGK    750
Assam macaque                      GPMCFLMLTLLLVAYMVVPVYVGPPKVSTCFCRQALFPLCFTICISCIAVRSFQIVCVFKMASRFPRAYSYWVRYQGPYVSMAFITVLKMVTVVIGMLVTGLNPTTRIDPDDPKIMIVSCNPNYRNSLFFNTGLDLLLSVVGFSFAYMGK    750
Stump-tailed macaque               GPMCFLMLTLLLVAYMVVPVYVGPPKVSTCFCRQALFPLCFTICISCIAVRSFQIVCVFKMASRFPRAYSYWVRYQGPYVSMAFITVLKMVTVVIGMLATGLNPTTRIDPDDPKIMIVSCNPNYRNSLFFNTGLDLLLSVVGFSFAYMGK    750
Japanese macaque                   GPMCFLMLTLLLVAYMVVPVYVGPPKVSTCFCRQALFPLCFTICISCIAVRSFQIVCVFKMASRFPRAYSYWVRYQGPYVSMAFITVLKMVTVVIGMLVTGLNPTTRIDPDDPKIMIVSCNPNYRNSLFFNTGLDLLLSVVGFSFAYMGK    750
Crab-eating macaque                GPMCFLMLTLLLVAYMVVPVYVGPPKVSTCFCRQALFPLCFTICISCIAVRSFQIVCVFKMASRFPRAYSYWVRYQGPYVSMAFITVLKMVTVVIGMLVTGLNPTTRIDPDDPKIMIVSCNPNYRNSLFFNTGLDLLLSVVGFSFAYMGK    750
Pig-tailed macaque                 GPMCFLMLTLLLVAYMVVPVYVGPPKVSTCFCRQALFPLCFTICISCIAVRSFQIVCVFKMASRFPRAYRYWVRYQGPYVSMAFITVLKMVTVVIGMLATGLNPTTRIDPDDPKIMIVSCNPNYRNSLFFNTGLDLLLSVVGFSFAYMGK    750
Rhesus macaque                     GPMCFLMLTLLLVAYMVVPVYVGPPKVSTCFCRQALFPLCFTICISCIAVRSFQIVCVFKMASRFPRAYSYWVRYQGPYVSMAFITVLKMVTVVIGMLATGLNPTTRIDPDDPKIMIVSCNPNYRNSLFFNTGLDLLLSVVGFSFAYMGK    750
Gelada baboon                      GPMCFLMLTLLLVAYMVVPVYVGPPKVSTCFCRQALFPLCFTICISCIAVRSFQIVCVFKMASRFPRAYSYWVRYQGPYVSMAFITVLKMVTVVIGMLATGLNPTTRIDPDDPKIMIVSCNPNYRNSLFFNTGLDLLLSVVGFSFAYMGK    750
Hamadryas baboon                   GPMCFLMLTLLLVAYMVVPVYVGPPKVSTCFCRQALFPLCFTICISCIAVRSFQIVCVFKMASRFPRAYSYWVRYQGPYVSMAFITVLKMVTVVIGMLATGLNPTTRIDPDDPKIMIVSCNPNYRNSLFFNTGLDLLLSVVGFSFAYMGK    750
Black crested mangabey             GPMCFLMLTLLLVAYMVVPVYVGPPKVSTCFCRQALFPLCFTICISCIAVRSFQIVCVFKMASRFPRAYSYWVRYQGPYVSMAFITVLKMVTVVIGMLATGLNPTTRIDPDDPKIMIVSCNPNYRNSLFFNTSLDLLLSVVGFSFAYMGK    750
Mandrill                           GPMCFLMLTLLLVAYMVVPVYVGPPKVSTCFCRQALFPLCFTICISCIAVRSFQIVCVFKMASRFPRAYSYWVRYQGPYVSMAFITVLKMVTVVIGMLATGLNPTTRIDPDDPKIMIVSCNPNYRNSLFFNTSLDLLLSVVGFSFAYMGK    750
Drill                              GPMCFLMLTLLLVAYMVVPVYVGPPKVSTCFCRQALFPLCFTICISCIAVRSFQIVCVFKMASRFPRAYSYWVRYQGPYVSMAFITVLKMVTVVIGMLATGLNPTTRIDPDDPKIMIVSCNPNYRNSLFFNTSLDLLLSVVGFSFAYMGK    750
Red guenon                         GPMCFLMLTLLLVAYMVVPVYVGPPKVSTCFCRQALFPLCFTICISCIAVRSFQIVCVFKMASRFPRAYSYWVRYQGPYVSMAFITVLKMVTVVIGMLATGLNPTTRIDPDDPKIMIVSCNPNYRNSLFFNTSLDLLLSVVGFSFAYMGK    750
Ma's night monkey                  GPMCFLMLTLLLVAYTVVPVYVGPPKVSTCLCRQALFPVCFTICISCITVRSFQIVCVFKMASRFPRAYSYWVRYQGTYVSVAFITALKIVTVVISSLATGLNPTTRTDTDDPKIMIISCNPNYRNSLLFNTSLDLLLSVVGFSFAYMGK    745
White-tufted-ear marmoset          GPMCFLMLTLLLVAYMVVPVYVGPPKVTTCLCRQALFPVCFTICISCITMRSFQIVCVFKMASRFPRAYSYWVRYQGSYVSVAFITALKVVTVVISLLATGLNPTTRADTDDPKIMIISCNPNYRNSLLFNTSLDLLLSVVGFSFAYMGK    745
Panamanian white-faced capuchin    GPMCFLMLTLLLVAYMVVPVYVGLPKVSTCLCRQALFPVCFTICISCIAVRSFQIVCVFKMASRFPRAYSYWVRYQGSYVSVAFITALKMITVVISLLATGLNPTTRTDTDDPKIMIISCNPNYRNSLLFNTSLDLLLSVVGFSFAYMGK    745
Brown-capped capuchin              GPMCFLMLTLLLVAYMVVPVYVGLPKVSTCLCRQALFPVCFTICISCIAVRSFQIVCVFKMASRFPRAYSYWVRYQGSYVSVAFITALKMITVVISLLATGLNPTTRTDTDDPKIMIISCNPNYRNSLLFNTSLDLLLSVVGFSFAYMGK    745
Bolivian squirrel monkey           GPMCFLMLTLLLVAYMVVPVYVGLPKVSTCLCRQALFPVCFTICISCIAVRSFQIVCVFKMASRFPRAYSYWVRYQGSYVSVAFITALKMVTVVISLLATGLNPTTRTDTDDPKIMIISCNPNYRNSLLFNTSLDLLLSVAGFSFAYMGK    745
Common squirrel monkey             GPMCFLMLTLLLVAYMVVPVYVGLPKVSTCLCRQALFPVCFTICISCIAVRSFQIVCVFKMASRFPRAYSYWVRYQGSYVSVAFITALKMVTVVISLLATGLNPTTRTDTDDPKIMIISCNPNYRNSLLFNTSLDLLLSVAGFSFAYMGK    745
Coquerel's sifaka                  GPMCFLMLTPLLVAYMVVPVYVGSPTVSTCLCRQTLFPICFTICISCITVRSFQIVCIFKMASRLPRAYSYWVRYHGPYVSVGFITALKVATVVGILLTTSPNPTTRADPDDPQIMILSCNPNFRGLLLFSTGLDLALSVVGFSFAYMGK    749
Gray mouse lemur                   GPMCFLMLAPLLVSYMVVPVYIGPPTAATCLCRQTLFPICFTVCISCITVRSFQIVYVFKMASHLPRAYGYWVRYHGPCVSVAFITSLKVAIVVGNLLTTSPSPTTRADPDDPQIMILSCNPSFRGLLMISTGLDLALSVVGFSFAYMGK    749
Ring-tailed lemur                  GPMCFLMLTPLLVAYTVVPVYVGLPTVSTCLWRLTVFPICFTVCISCITVRSFQIVYIFKMASRLPRAYSYWVRYHGPYVSVVVTTALKVAIVVGNLLTTSPSPTARADPDDPQVMILSCNPNFRSLLLVNAGLDLALSVAGFGFAYVGK    749
```

```
                                 *************:*********:*******.  .******:******:*********************:***  *****:******  :*
Human                            ELPTNYNEAKFITLSMTFYFTSSVSLCTFMSAYSGVLVTIVDLLVTVLNLLAISLGYFGPKCYMILFYPERNTPAYFNSMIQGYTMRRD   839
Western lowland gorilla          ELPTNYNEAKFITLSMTFYFTSSVSLCTFMSAYSGVLVTIVDLLVTVLNLLAISLGYFGPKCYMILFYPERNTPAYFNSMIQGYTMRRD   839
Sumatran orangutan               ELPTNYNEAKFITLSMTFYFTSSISLCTFMSAYSGVLVTIVDLLVTVLNLLAISLGYFGPKCYMILFYPERNTPAYFNSVIQGYTMTRD   839
Bornean orangutan                ELPTNYNEAKFITLSMTFYFTSSISLCTFMSAYSGVLVTIVDLLVTVLNLLAISLGYFGPKCYMILFYPERNTPAYFNSVIQGYTMTRD   839
Pygmy chimpanzee                 ELPTNYNEAKFITLSMTFYFTSSVSLCTFMSAYSGVLVTIVDLLVTVLNLLAISLGYFGPKCYMILFYPERNTPAYFNSMIQGYTMRRD   839
Common chimpanzee                ELPTNYNEAKFITLSMTFYFTSSVSLCTFMSAYSGVLVTIVDLLVTVLNLLAISLGYFGPKCYMILFYPERNTSAYFNSMIQGYTMRRD   839
Northern white-cheeked gibbon    ELPTNYNEAKFITLSMTFYFTSSVSLCTFMSAYNGVLVTIVDLLVTVLNLLAISLGYFGPKCYMILFYPERNTPAYFNSMIQGYTMRRD   839
Northern buffed-cheeked gibbon   ELPTNYNEAKFITLSMTFYFTSSVSLCTFMSAYNGVLVTIVDLLVTVLNLLAISLGYFGPKCYMILFYPERNTPAYFNSMIQGYTMRRD   839
Western hoolock gibbon           ELPTNYNEAKFITLSMTFYFTSSVSLCTFMSAYNGVLVTIVDLLVTVLNLLAISLGYFGPKCYMILFYPERNTPAYFNSMIQGYTMRRD   839
Hylobates muelleri abbotti       ELPTNYNEAKFITLSMTFYFTSSVSLCTFMSAYNGVLVTIVDLLVTVLNLLAISLGYFGPKCYMILFYPERNTPAYFNSMIQGYTMRRD   839
Agile gibbon                     ELPTNYNEAKFITLSMTFYFTSSVSLCTFMSAYNGVLVTIVDLLVTVLNLLAISLGYFGPKCYMILFYPERNTPAYFNSMIQGYTMRRD   839
Common gibbon                    ELPTNYNEAKFITLSMTFYFTSSVSLCTFMSAYNGVLVTIVDLLVTVLNLLAISLGYFGPKCYMILFYPERNTPAYFNSMIQGYTMRRD   839
Pileated gibbon                  ELPTNYNEAKFITLSMTFYFTSSVSLCTFMSAYNGVLVTIVDLLVTVLNLLAISLGYFGPKCYMILFYPERNTPAYFNSMIQGYTMRRD   839
Siamang                          ELPTNYNEAKFITLSMTFYFTSSVSLCTFMSAYNGVLVTIVDLLVTVLNLLAISLGYFGPKCYMILFYPERNTPAYFNSMIQGYTMRRD   839
Green monkey                     ELPTNYNEAKFITLSMTFYFTSSVSLCTFMSASNGVLVTIMDLLVTVLNLLAISLGYFGPKCYMILFYPERNTPAYFNSMIQGYTMRRD   839
Golden-bellied mangabey          ELPTNYNEAKFITLSMTFYFTSSVSLCTFMSAYNGVLVTIMDLLVTVLNLLAISLGYFGPKCYMILFYPERNTPAYFNSMIQGYTMRRD   839
Sooty mangabey                   ELPTNYNEAKFITLSMTFYFTSSVSLCTFMSAYNGVLVTIMDLLVTVLNLLAISLGYFGPKCYMILFYPERNTPAYFNSMIQGYTMRRD   839
Blue monkey                      ELPTNYNEAKFITLSMTFYFTSSVSLCTFMSAYNGVLVTIMDLLVTVLNLLAISLGYFGPKCYMILFYPERNTPAYFNSMIQGYTMRRD   839
Sykes' monkey                    ELPTNYNEAKFITLSMTFYFTSSVSLCTFMSAYNGVLVTIMDLLVTVLNLLAISLGYFGPKCYMILFYPERNTPAYFNSMIQGYTMRRD   839
Assam macaque                    ELPTNYNEAKFITLSMTFYFTSSVSLCTFMSAYNGVLVTIMDLLVTVLNLLAISLGYFGPKCYMILFYPERNTPAYFNSMIQGYTMRRD   839
Stump-tailed macaque             ELPTNYNEAKFITLSMTFYFTSSVSLCTFMSAYNGVLVTIMDLLVTVLNLLAISLGYFGPKCYMILFYPERNTPAYFNSMIQGYTMRRD   839
Japanese macaque                 ELPTNYNEAKFITLSMTFYFTSSVSLCTFMSAYNGVLVTIMDLLVTVLNLLAISLGYFGPKCYMILFYPERNTPAYFNSMIQGYTMRRD   839
Crab-eating macaque              ELPTNYNEAKFITLSMTFYFTSSVSLCTFMSAYNGVLVTIMDLLVTVLNLLAISLGYFGPKCYMILFYPERNTPAYFNSMIQGYTMRRD   839
Pig-tailed macaque               ELPTNYNEAKFITLSMTFYFTSSVSLCTFMSAYNGVLVTIMDLLVTVLNLLAISLGYFGPKCYMILFYPERNTPAYFNSMIQGYTMRRD   839
Rhesus macaque                   ELPTNYNEAKFITLSMTFYFTSSVSLCTFMSAYNGVLVTIMDLLVTVLNLLAISLGYFGPKCYMILFYPERNTPAYFNSMIQGYTMRRD   839
Gelada baboon                    ELPTNYNEAKFITLSMTFYFTSSVSLCTFMSAYNGVLVTIMDLLVTVLNLLAISLGYFGPKCYMILFYPERNTPAYFNSMIQGYTMRRD   839
Hamadryas baboon                 ELPTNYNEAKFITLSMTFYFTSSVSLCTFMSAYNGVLVTIMDLLVTVLNLLAISLGYFGPKCYMILFYPERNTPAYFNSMIQGYTMRRD   839
Black crested mangabey           ELPTNYNEAKFITLSMTFYFTSSVSLCTFMSAYNGVLVTIMDLLVTVLNLLAISLGYFGPKCYMILFYPERNTPAYFNSMIQGYTMRRD   839
Mandrill                         ELPTNYNEAKFITLSMTFYFTSSVSLCTFMSVYNGVLVTIMDLLVTVLNLLAISLGYFGPKCYMILFYPERNTPAYFNSMIQGYTMRRD   839
Drill                            ELPTNYNEAKFITLSMTFYFTSSVSLCTFMSVYNGVLVTIMDLLVTVLNLLAISLGYFGPKCYMILFYPKRNTPAYFNSMIQGYTMRRD   839
Red guenon                       ELPTNYNEAKFITLSMTFYFTSSVSLCTFMSAYNGVLVTIMDLLVTVLNLLAISLGYFGPKCYMILFYPERNTPAYFNSMIQGYTMRRD   839
Ma's night monkey                ELPTNYNEAKFITFSMTFYFTSSVSLCTFMSVYNGVLVTIVDLLVTVFNLLAISLGYFGPKCYMILFYPERNTPAYFNSMIQGYTMRRD   834
White-tufted-ear marmoset        ELPTNYNEAKFITFSMTFYFTSSVSLCTFMSVYDGVLVTIVDLLVTVFNLLAISLGYFGPKCYMILFYPERNTPAYFNSMIQGYTMRRD   834
Panamanian white-faced capuchin  ELPTNYNEAKFITFSMTFYFTSSVSLCTFMSVYDGVLVTIVDLLVTVFNLLAISLGYFGPKCYMILFYPERNTPAYFNSMIQGYTMRRD   834
Brown-capped capuchin            ELPTNYNEAKFITFSMTFYFTSSVSLCTFMSVYDGVLVTIVDLLVTVFNLLAISLGYFGPKCYMILFYPERNTPAYFNSMIQGYTMRRD   834
Bolivian squirrel monkey         ELPTNYNEAKFITFSMTFYFTSSVSLCTFMSVYDGVLVTIVDLLVTVFNLLAISLGYFGPKCYMILFYPERNTPAYFNSMIQGYTMRRD   834
Common squirrel monkey           ELPTNYNEAKFITFSMTFYFTSSVSLCTFMSVYDGVLVTIVDLLVTVFNLLAISLGYFGPKCYMILFYPERNTPAYFNSMIQGYTMRRD   834
Coquerel's sifaka                ELPTNYNEAKFITLSMTFYFTSSVSLCTFMSVYSGVLVTIMDLLVTVLNLLAISLGYFGPKCYMILFYPERNTQAYFNSMIQGYTMRKD   838
Gray mouse lemur                 ELPTNYNEAKFITLSMTFYFTSSVSLCTFMSVYSGVLVTIMDLLVTVLNLLAISLGYFGPKCYMILFYPERNTQAYFNSMIQGYTMRKD   838
Ring-tailed lemur                ELPTNYNEAKFITLSMTFYFTSSVSLCTFMSVYSGVLVTIMDLLVTVLNLLAISLGYFGPKCYMILFYPERNTQAYFNSMIQGYTMGKD   838
```
