# Supplementary material for: Systematic analysis reveals novel insight into the molecular determinants of function, diversity and evolution of sweet taste receptors T1R2/T1R3 in primates
Source: Front Mol Biosci. 2023 Jan 25;10:1037966. doi: 10.3389/fmolb.2023.1037966 (PMC9905694; doi:10.3389/fmolb.2023.1037966)
Supplement: Supplementary file 2 [file Table1.DOCX]

**Supplemental Table 1** Key residues involved in recognizing different sweeteners and their locations.

| **Sweeteners** | **Key residues** | **Subunit location** | **Domain** |
| --- | --- | --- | --- |
| Aspartame | S40, Y103, D142, Y215, P277, D278, L279, S303, D307, R383 and V384 | T1R2 | VFTM |
| Brazzein | I536, A537 and F540 | T1R3 | CRD |
| Thaumatin | Q504, A537, R556, S559 and R560 | T1R3 | CRD |
| Cyclamate | Q636, Q637, H641, H721, | T1R3 | TMD |
|  | R723, F778 and L782 |  |  |
