# Supplementary figures and images for: Systematic analysis reveals novel insight into the molecular determinants of function, diversity and evolution of sweet taste receptors T1R2/T1R3 in primates
Source: Front Mol Biosci. 2023 Jan 25;10:1037966. doi: 10.3389/fmolb.2023.1037966 (PMC9905694; doi:10.3389/fmolb.2023.1037966)

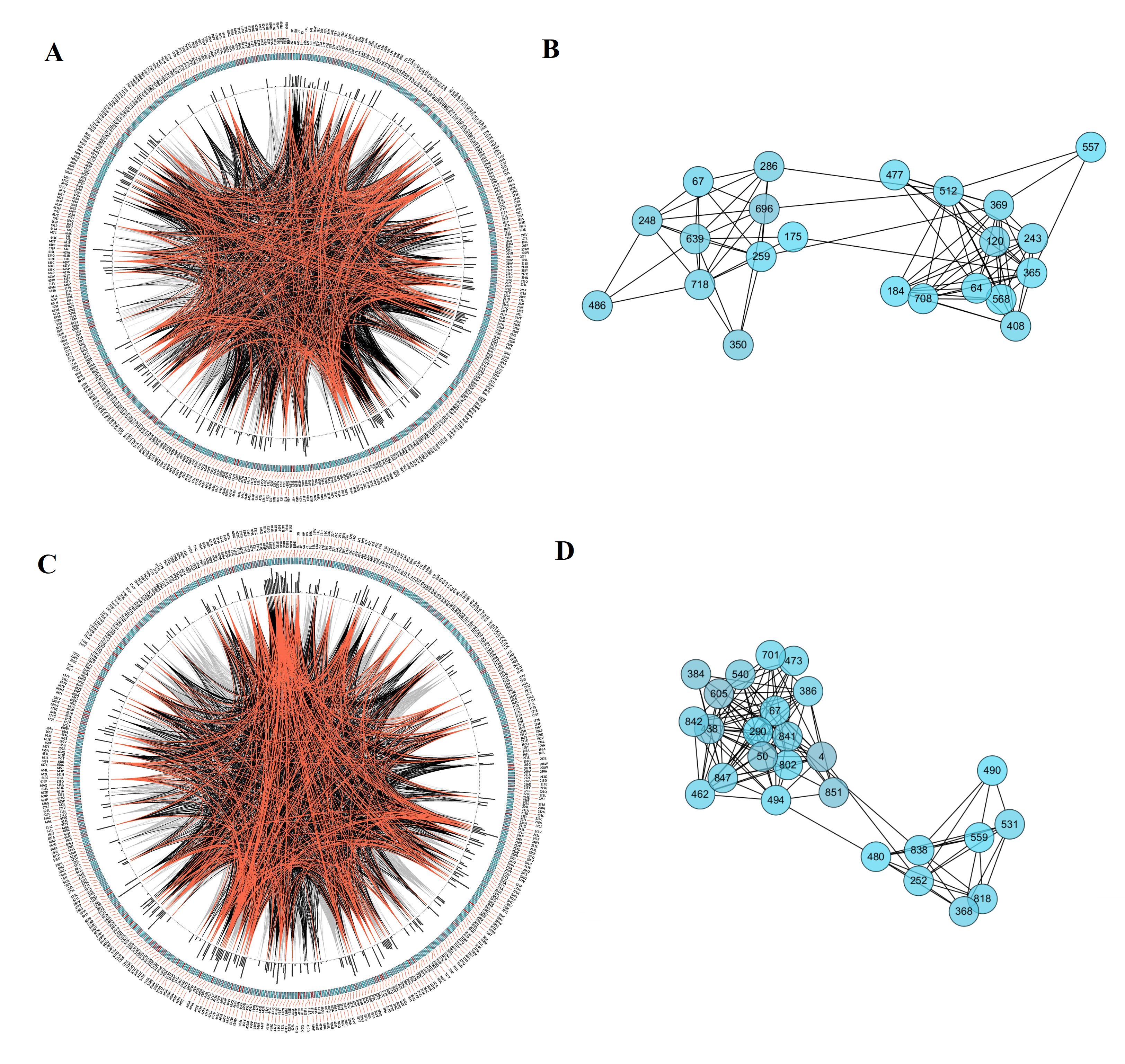

Supplement: Supplementary file 3 [file Image1.tif]
