# Supplementary material for: Systematic analysis reveals novel insight into the molecular determinants of function, diversity and evolution of sweet taste receptors T1R2/T1R3 in primates
Source: Front Mol Biosci. 2023 Jan 25;10:1037966. doi: 10.3389/fmolb.2023.1037966 (PMC9905694; doi:10.3389/fmolb.2023.1037966)
Supplement: Supplementary file 5 [file DataSheet5.pdf]

```
                                 *    .***:.* :.*     * * ***:**:*.***:****.*** * :..: .: :* . **:*:*  ******::.:********   **.**:**:*:******..*:*****:***. .* :*****:**:*:****************
Human                            MLGPAVLGLSLWALLHPGTGAPLCLSQQLRMKGDYVLGGLFPLGEAEEAGLRSRTRPSSPVCTRFSSNGLLWALAMKMAVEEINNKSDLLPGLRLGYDLFDTCSEPVVAMKPSLMFLAKAGSRDIAAYCNYTQYQPRVLAVIGPHSSELA   150
Western lowland gorilla          MLGPAVLGLSLWALLQPGAGAPLCLSQQLRMKGDYMLGGLFPLGEAEEAGFRSRTRPSSPVCTRFSSNGLLWALAMKMAVEEINNKSDLLPGLRLGYDLFDTCSEPVVAMKPSLMFLAKAGSRDIAAYCNYTQYQPRVLAVIGPHSSELA   150
Sumatran orangutan               MLGPAVLGLSLWALLHSGTGAPLCLSQQLRMKGDYVLGGLFPLGEAEEAGLRSRTRPSSPVCTRFSSNGLLWALAMKMAVEEINNKSDLLPGLRLGYDLFDTCSEPVVAMKPSLMFLAKADSRDIAAYCNYTQYQPRVLAVIGPHSSELA   150
Bornean orangutan                MLGPAVLGLSLWALLHSGTGAPLCLSQQLRMKGDYVLGGLFPLGEAEEAGLRSRTRPSSPVCTRFSSNGLLWALAMKMAVEEINNKSDLLPGLRLGYDLFDTCSEPVVAMKPSLMFLAKADSRDIAAYCNYTQYQPRVLAVIGPHSSELA   150
Pygmy chimpanzee                 MLGPAVLGLSLWALLHPGTGAPLCLSQQLRMKGDYVLGGLFPLGEAEEAGLRSRTRPSSPVCTRFSSNGLLWALAMKMAVEEINNKSDLLPGLRLGYDLFDTCSEPVVAMKPSLVFLAKAGSRDIAAYCNYTQYQPRVLAVIGPHSSELA   150
Common chimpanzee                MLGPAVLGLSLWALLHPGTGAPLCLSQQLRMKGDYVLGGLFPLGEAEEAGLRSRTRPSSPVCTRFSSNGLLWALAMKMAVEEINNKSDLLPGLRLGYDLFDTCSEPVVAMKPSLVFLAKAGSRDIAAYCNYTQYQPRVLAVIGPHSSELA   150
Northern white-cheeked gibbon    MLGPAVLGLSLWALLHPGTGAPLCLSQQLRMKGDYVLGGLFPLGEAEEAGIRSRTRPSSPVCTRFSSNGLLWALAMKMAVEEINNKSDLLPGLRLGYDLFDTCSEPVVAMKPSLMFLARADSRDIAAYCNYTQYQPRVLAVIGPHSSELA   150
Northern buffed-cheeked gibbon   MLGPAVLGLSLWALLHPGTGAPLCLSQQLRMKGDYVLGGLFPLGEAEEAGIRSRTRPSSPVCTRFSSNGLLWALAMKMAVEEINNKSDLLPGLRLGYDLFDTCSEPVVAMKPSLMFLARADSRDIAAYCNYTQYQPRVLAVIGPHSSELA   150
Western hoolock gibbon           MLGPAVLGLSLWALLHPGMGAPLCLSQQLRMKGDYVLGGLFPLGEAEEAGIHSRTRPSSPVCTRFSSNGLLWALAMKMAVEEINNKSDLLPGLRLGYDLFDTCSEPVVAMKPSLMFLARADSRDIAAYCNYTQYQPRVLAVIGPHSSELA   150
Hylobates muelleri abbotti       MLGPAVLGLSLWALLHPGTGAPLCLSQQLRMKGDYMLGGLFPLGEAEEAGIRSRTRPSSPVCTRFSSNGLLWALAMKMAVEEINNKSDLLPGLRLGYDLFDTCSEPVVAMKPSLMFLARADSRDIAAYCNYTQYQPRVLAVIGPHSSELA   150
Agile gibbon                     MLGPAVLGLSLWALLHPGTGAPLCLSQQLRMKGDYMLGGLFPLGEAEEAGIRSRTRPSSPVCTRFSSNGLLWALAMKMAVEEINNKSDLLPGLRLGYDLFDTCSEPVVAMKPSLMFLARADSRDIAAYCNYTQYQPRVLAVIGPHSSELA   150
Common gibbon                    MLGPAVLGLSLWALLHPGTGAPLCLSQQLRMKGDYMLGGLFPLGEAEEAGIRSQTRPSSPVCTRFSSNGLLWALAMKMAVEEINNKSDLLPGLRLGYDLFDTCSEPVVAMKPSLMFLARADSRDIAAYCNYTQYQPRVLAVIGPHSSELA   150
Pileated gibbon                  MLGPAVLGLSLWALLHPGTGAPLCLSQQLRMKGDYMLGGLFPLGEAEEAGIRSRTRPSSPVCTRFSSNGLLWALAMKMAVEEINNKSDLLPGLRLGYDLFDTCSEPVVAMKPSLMFLARADSRDIAAYCNYTQYQPRVLAVIGPHSSELA   150
Siamang                          MLGPAVLGLSLWALLHPGTGAPLCLSQQLRMKGDYMLGGLFPLGEAEEAGIRSRTRPSSPVCTRFSSNGLLWALAMKMAVEEINNKSDLLPGLRLGYDLFDTCSEPVVAMKPSLMFLARADSRDIAAYCNYTRYQPRVLAVIGPHSSELA   150
Green monkey                     MLHPAVLGLSLWALLHLGTGAPLCLSQQLRMKGDYVLGGLFPLGEAEEAGLGSRTRPSSPVCTRFSSNGLLWALAMKMAVEEINNRSDLLPGLRLGHDLFDTCSEPVVAMKPSLMFLAKADSRNIAAYCNYTQYQPRVLAVIGPHSSELA   150
Golden-bellied mangabey          MLRPAVLGLSLWAVLHLGTGAPLCLSQQLRMKGDYVLGGLFPLGEAEEAGLGSRTRPSSPVCTRFSSNGLLWALAMKMAVEEINNRSDLLPGLRLGHDLFDTCSEPVVAMKPSLMFLAKADSRDIAAYCNYTQYQPRVLAVIGPHSSELA   150
Sooty mangabey                   MLRPAVLGLSLWAVLHLGTGAPLCLSQQLRMKGDYVLGGLFPLGEAEEAGLGSRTRPSSPVCTRFSSNGLLWALAMKMAVEEINNRSDLLPGLRLGHDLFDTCSEPVVAMKPSLMFLAKADSRDIAAYCNYTQYQPRVLAVIGPHSSELA   150
Blue monkey                      MLRPAVLGLSLWALLHLGTGAPLCLSQQLRMKGDYVLGGLFPLGEAEEAGLGSRTRPSSPVCTRFSSNGLLWALAMKMAVEEINNRSDLLPGLRLGHDLFDTCSEPVVAMKPSLMFLAKADSRDIAAYCNYTQYQPRVLAVIGPHSSELA   150
Sykes' monkey                    MLRPAVLGLSLWALLHLGTGAPLCLSQQLRMKGDYVLGGLFPLGEAEEAGLGSRTRPSSPVCTRFSSNGLLWALAMKMAVEEINNRSDLLPGLRLGHDLFDTCSEPVVAMKPSLMFLAKADSRDIAAYCNYTQYQPRVLAVIGPHSSELA   150
Assam macaque                    MLCPAVLGLSLWALLHLGTGAPLCLSQQLRMKGDYVLGGLFPLGEAEEAGLGSRTRPSSPVCTRFSSNGLLWALAMKMAVEEINNRSDLLPGLRLGHDLFDTCSEPVVAMKPSLMFLAKADSRDIAAYCNYTQYQPRVLAVIGPHSSELA   150
Stump-tailed macaque             MLCPAVLGLSLWALLHLGTGAPLCLSQQLRMKGDYVLGGLFPLGEAEEAGLGSRTRPSSPVCTRFSSNGLLWALAMKMAVEEINNRSDLLPGLRLGHDLFDTCSEPVVAMKPSLMFLAKADSRDIAAYCNYTQYQPRVLAVIGPHSSELA   150
Japanese macaque                 MLRPAVLGLSLWALLHLGTGAPLCLSQQLRMKGDYVLGGLFPLGEAEEAGLGSRTRPSSPVCTRFSSNGLLWALAMKMAVEEINNRSDLLPGLRLGHDLFDTCSEPVVAMKPSLMFLAKADSRDIAAYCNYTQYQPRVLAVIGPHSSELA   150
Crab-eating macaque              MLCPAVLGLSLWALLHLGTGAPLCLSQQLRMKGDYVLGGLFPLGEAEEAGLGSRTRPSSPVCTRFSSNGLLWALAMKMAVEEINNRSDLLPGLRLGHDLFDTCSEPVVAMKPSLMFLAKADSRDIAAYCNYTQYQPRVLAVIGPHSSELA   150
Pig-tailed macaque               MLCPAVLGVSLWALLHLGTGAPLCLSQQLRMKGDYVLGGLFPLGEAEEAGLGSRTRPSSPVCTRFSSNGLLWALAMKMAVEEINNRSDLLPGLRLGHDLFDTCSEPVVAMKPSLMFLAKADSRDIAAYCNYTQYQPRVLAVIGPHSSELA   150
Rhesus macaque                   MLCPAVLGLSLWALLHLGTGAPLCLSQQLRMKGDYVLGGLFPLGEAEEAGLGSRTRPSSPVCTRFSSNGLLWALAMKMAVEEINNRSDLLAGLRLGHDLFDTCSEPVVAMKPSLMFLAKADSRDIAAYCNYTQYQPRVLAVIGPHSSELA   150
Gelada baboon                    MLRPAVLGLSLWALLHLGTGAPLCLSQQLRMKGDYVLGGLFPLGEAEEAGLSSRTRPSSPVCTRLSSNGLLWALAMKMAVEEINNRSDLLPGLRLGHDLFDTCSEPVVAMKPSLMFLAKADSRNIAAYCNYTQYQPRVLAVIGPHSSELA   150
Hamadryas baboon                 MLRPAVLGLSLWALLHLGTGAPLCLSQQLRMKGDYVLGGLFPLGEAEEAGLRSRTRPSSPVCTRFSSNGLLWALAMKVAVEEINNKSDLLPGLRLGYDLFDTCSEPVVAMKPSLMFLAKAGSRDIAAYCNYTQYQPRVLAVIGPHSSELA   150
Mandrill                         MLRPAVLGLSLWAVLHLGTGAPLCLSQQLRMKGDYVLGGLFPLGEAEEAGLGSRTRPSSPVCTRFSSNGLLWALAMKMAVEEINNRSDLLPGLRLGHDLFDTCSEPVVAMKPSLMFLAKADSRDIAAYCNYTQYQPRVLAVIGPHSSELA   150
Drill                            MLRPAVLGLSLWAVLHLGTGAPLCLSQQLRMKGDYVLGGLFPLGEAEEAGLGSRTRPSSPVCTRFSSNGLLWALAMKMAVEEINNRSDLLPGLRLGHDLFDTCSEPVVAMKPSLMFLAKADSRDIAAYCNYTQYQPRVLAVIGPHSSELA   150
Red guenon                       MLRPAVLGLSLWALLHLGTGAPLCLSQQLRMKGDYVLGGLFPLGEAEEVGLGSRTRPSSPVCTRFSSNGLLWALAMKMAVEEINNRSDLLPGLRLGHDLFDTCSEPVVAMKPSLMFLAKADSRNIAAYCNYTQYQPRVLAVIGPHSSELA   150
Ma's night monkey                MLGSAVLGLSLWALLHLRTGAPLCLSRQLKMKGDYMLGGLFPLGEAGEAALHSRTRPTSLVCTRFSWNGLLWALTMKMAVEEINNRSDLLPGLRLGYDLFDTCSESVVTMKPSLMFLAKANSHDIAAYCNYTQYQPRVLAVIGPHSSELA   150
White-tufted-ear marmoset        MLGSAVLGLSLWALLHLRTGAPLCLSRQLKMKGDYMLGGLFPLGEAGEAAFHSRTRPSSLVCTRFSWNGLLWALAMKMAVEEINNQSDLLPGLRLGYDLFDTCSESVVTMKPSLMFLAKVNSHDIAAYCNYTQYQPRVLAVIGPHSSELA   150
Panamanian white-faced capuchin  MVGSAVLGLGLWALLHLRTGAPLCLSQQLKMKGDYVLGGLFPLGEAGETALHSRTQPNSLVCTRFSWNGLLWALAMKMAVEEINNRSDLLPGLRLGYDVFDTCSEPTVTMKPSLMFLAKANSHDIAAYCNYTQYQPRVLAVIGPHSSELA   150
Brown-capped capuchin            MLGSAVLGLGLWALLHLRTGAPLCLSQQLKMKGDYVLGGLFPLGEAGETALHSRTRPNSLVCTRFSWNGLLWALAMKMAVEEINNRSDLLPGLRLGYDLFDTCSEPTVTMKPSLMFLAKANSHDIAAYCNYTQYQPRVLAVIGPHSSELA   150
Bolivian squirrel monkey         MLGSGVLGLSLWTLLHLRTGAPSCLSRQLKMKGDYVLGGLFPLGEAGEAALHSRTRPSSLVCTRFSWNGLLWALAMKMAVEEINNRLDLLPGLRLGYDLFDTCSEPTVTMKPSLMFLAKANSHDIAAYCNYTQYQPRVLAVIGPHSSELA   150
Common squirrel monkey           MLGSGVLGLSLWTLLHLRTGAPSCLSRQLKMKGDYVLGGLFPLGEAGEAALHSRTRPSSLVCTRFSWNGLLWALAMKMAVEEINNRLDLLPGLRLGYDLFDTCSEPTVTMKPSLMFLAKANSHDIAAYCNYTQYQPRVLAVIGPHSSELA   150
Coquerel's sifaka                MPSLAVLGLSLAALLSPGTGAPLCLSQQLKMSGDYMLGGLFPLGTAEEASLRGRMQPSSPVCTRFSAPGLLWALTVKMAVEEINNESTLLPGLRLGYDLFDTCSEPVVAMKPSLMFLAEIGSRDIAAYCDYTRYRPRVLAVIGPHSSELA   150
Gray mouse lemur                 MPSLAVLGLSLAALLSPGTGDPLCLSRQLKMSGDYVLGGLFPLGTAEDASLRGRTQPRGPVCSRFSALGLLWALTVTMAVEEINNGSALLPGLRLGYDLFDTCSEPVVAMKPSLMFLARTGSCDIAAYCDYTRYRPRVLAVIGPHSSELA   150
Ring-tailed lemur                MPSFAVLGLSLAAFLSPGMGAPLCLSRQLKMSGDYVLGGLVPLGTAEETSLRGRTQPSSPVCTRFSAVGLLWALTVKMAVEEINNGSALLPGLHLGYDLFDTCSEPVVAMKPSLMFLARVGSCDIAAYCDYTRYRPRVLAVIGPHSSELA   150
```

```
                                  :******.***:*******.*: **:****************** * .:***.:********************* **.** ::************.:::    .***::* *:**:.:***:***: :**  :* : *  :*  ******
Human                             MVTGKFFSFFLMPQVSYGASMELLSARETFPSFFRTVPSDRVQLTAAAELLQEFGWNWVAALGSDDEYGRQGLSIFSALAAARGICIAHEGLVPLPRADDSRLGKVQDVLHQVNQSSVQVVLLFASVHAAHALFNYSISSRLSPKVWVAS   300
Western lowland gorilla           MVTGKFFSFFLMPQVSYGASMELLSARETFPSFFRTVPSDRVQLTAAAELLQEFGWNWVAALGSDDEYGRQGLSIFSALAAARGICIAHEGLVPLPRADDSRLGKVQDVLHQVNQSSVQVVLLFASVHAAHALFNYSISSRLSPKVWVAS   300
Sumatran orangutan                LVTGKFFSFFLMPQVSYGASMELLSARETFPSFFRTVPSDRVQLTAAAELLQQFGWNWVAALGSDDEYGRQGLSIFSALAAARGICIAHEGLVPLPRADDLRLGKVQDVLHQVNQSSVQVVLLFASVHAAYALFNYSISSRLSPKVWVAS   300
Bornean orangutan                 LVTGKFFSFFLMPQVSYGASMELLSARETFPSFFRTVPSDRVQLTAAADLLQQFGWNWVAALGSDDEYGRQGLSIFSALAAARGICIAHEGLVPLPRADDLRLGKVQDVLHQVNQSSVQVVLLFASVHAAYALFNYSISSRLSPKVWVAS   300
Pygmy chimpanzee                  MVTGKFFSFFLMPQVSYGASMELLSARETFPSFFRTVPSDRVQLTAAAELLQEFGWNWVAALGSDDEYGRQGLSIFSALAAARGICIAHEGLVPLPRADDSRLGKVQDVLHQVNQSSVQVVLLFASVHAAHALFNYSISSRLSPKVWVAS   300
Common chimpanzee                 MVTGKFFSFFLMPQVSYGASMELLSARETFPSFFRTVPSDRVQLTAAAELLQEFGWNWVAALGSDDEYGRQGLSIFSALAAARGICIAHEGLVPLPRADDSRLGKVQDVLHQVNQSSVQVVLLFASVHAAHALFNYSISSRLSPKVWVAS   300
Northern white-cheeked gibbon     LVTGKFFSFFLMPQVSYGASMELLSARETFPSFFRTVPSDRVQLTAAAELLQEFGWNWVAALGSDDEYGRQGLSIFSALAAARGICIAHEGLVPLPRADDLRLGKVQDVLHQVNQSNVQVVLLFASVHAAHALFNYSISSKLSPKVWVAS   300
Northern buffed-cheeked gibbon    LVTGKFFSFFLMPQVSYGASMELLSARETFPSFFRTVPSDRVQLTAAAELLQEFGWNWVAALGSDDEYGRQGLSIFSALAAARGICIAHEGLVPLPRADDLRLGKVQDVLHQVNQSNVQVVLLFASVHAAHALFNYSISSKLSPKVWVAS   300
Western hoolock gibbon            LVTGKFFSFFLMPQVSYGASMELLSARETFPSFFRTVPSDRVQLTAAAELLQEFGWNWVAALGSDDEYGRQGLSIFSALAAARGICIAHEGLVPLPHADDLRLGKVQDVLHQVNQSNVQVVLLFASVHAAHALFNYSISSRLSPKVWVAS   300
Hylobates muelleri abbotti        LVTGKFFSFFLMPQVSYGASMELLSARETFPSFFRTVPSDRVQLTAAAELLQQFGWNWVAALGSDDEYGRQGLSIFSALAAARGICIAHEGLVPLPRADDLRLGKVQDVLHQVNQSNVQVVLLFASVHAAHALFNYSISSRLSPKVWVAS   300
Agile gibbon                      LVTGKFFSFFLMPQVSYGASMELLSARETFPSFFRTVPSDRVQLTAAAELLQQFGWNWVAALGSDDEYGRQGLSIFSALAAARGICIAHEGLVPLPRADDLRLGKVQDVLHQVNQSNVQVVLLFASVHAAHALFNYSISSRLSPKVWVAS   300
Common gibbon                     LVTGKFFSFFLMPQVSYGASMELLSARETFPSFFRTVPSDRVQLTAAAELLQQFGWNWVAALGSDDEYGRQGLSIFSALAAARGICIAHEGLVPLPRADDLRLGKVQDVLHQVNQSNVQVVLLFASVHAAHALFNYSISSRLSPKVWVAS   300
Pileated gibbon                   LVTGKFFSFFLMPQVSYGASMELLSARETFPSFFRTVPSDRVQLTAAAELLQQFGWNWVAALGSDDEYGRQGLSIFSALAAARGICIAHEGLVPLPRADDLRLGKVQDVLHQVNQSNVQVVLLFASVHAAHALFNYSISSRLSPKVWVAS   300
Siamang                           LVTGKFFSFFLMPQVSYGASMELLSARETFPSFFRTVPSDRVQLTAAAELLQEFGWNWVAALGSDDEYGRQGLSIFSALAAARGICIAHEGLVPLPRADDLRLGKVQDVLHQVNQSNVQVVLLFASVHAAHALFNYSISSRLSPKVWVAS   300
Green monkey                      VVTGKFFGFFLMPQVSYGASMELLSARETFPSFFRTVPSDRVQLTAAAELLQEFGWNWVAALGSDDEYGRQGLSIFSALAAARGICIAHEGLVPLPRANSLLLGKVQEVLHQVNQSSVQVVLLFASARAAHALFSYSISSKLSRKVWVAS   300
Golden-bellied mangabey           VVTGKFFGFFLMPQVSYGASMELLSARETFPSFFRTVPSDRVQLTAAAELLQEFGWNWVAALGSDDEYGRQGLSIFSALAAARGICIAHEGLVPLPRANSPLLGKVQEVLHQVNQSSVQVVLLFASARAAHALFSYSISSKLSRKVWVAS   300
Sooty mangabey                    VVTGKFFGFFLMPQVSYGASMELLSARETFPSFFRTVPSDRVQLTAAAELLQEFGWNWVAALGSDDEYGRQGLSIFSALAAARGICIAHEGLVPLPRANSPLLGKVQEVLHQVNQSSVQVVLLFASARAAHALFSYSISSKLSRKVWVAS   300
Blue monkey                       VVTGKFFGFFLMPQVSYGASMELLSARETFPSFFRTVPSDRVQLTAAAELLQEFGWNWVAALGSDDEYGRQGLSIFSALAAARGICIAHEGLVPLPRANSPLLGKVQEVLHQVNQSSVQVVLLFASVRAAHALFSYSISSKLSRKVWVAS   300
Sykes' monkey                     VVTGKFFGFFLMPQVSYGASMELLSARETFPSFFRTVPSDRVQLTAAAELLQEFGWNWVAALGSDDEYGRQGLSIFSALAAARGICIAHEGLVPLPRANSPLLGKVQEVLHQVNQSSVQVVLLFASVRAAHALFSYSISSKLSRKVWVAS   300
Assam macaque                     VVTGKFFGFFLMPQVSYGAGMELLSARETFPSFFRTVPSDRVQLVAAAELLQEFGWNWVAALGSDDEYGRQGLSTFSALAASRGICIAHEGLVPLPRANSPLLGKVQEVLHQVNQSSVQVVLLFASARAAHALFSYSISSKLSRKVWVAS   300
Stump-tailed macaque              VVTGKFFGFFLMPQVSYGAGMELLSARETFPSFFRTVPSDRVQLVAAAELLQEFGWNWVAALGSDDEYGRQGLSTFSALAASRGICIAHEGLVPLPRANSPLLGKVQEVLHQVNQSSVQVVLLFASARAAHALFSYSISSKLSRKVWVAS   300
Japanese macaque                  VVTGKFFGFFLMPQVSYGAGMELLSARETFPSFFRTVPSDRVQLVAAAELLQEFGWNWVAALGSDDEYGRQGLSIFSALAASRGICIAHEGLVPLPRANSPLLGKVQEVLHQVNQSSVQVVLLFASARAAHALFSYSISSKLSRKVWVAS   300
Crab-eating macaque               VVTGKFFGFFLMPQVSYGAGMELLSARETFPSFFRTVPSDRVQLVAAAELLQEFGWNWVAALGSDDEYGRQGLSTFSALAASRGICIAHEGLVPLPRANSPLLGKVQEVLHQVNQSSVQVVLLFASARAAHALFSYSISSKLSRKVWVAS   300
Pig-tailed macaque                VVTGKFFGFFLMPQVSYGAGMELLSARETFPSFFRTVPSDRVQLVAAAELLQEFGWNWVAALGSDDEYGRQGLSTFSALAASRGICIAHEGLVPLPRANSPLLGKVQEVLHQVNQSSVQVVLLFASARAAHALFSYSISSKLSRKVWVAS   300
Rhesus macaque                    VVTGKFFGFFLMPQVSYGAGMELLSARETFPSFFRTVPSDRVQLVAAAELLQEFGWNWVAALGSDDEYGRQGLSIFSALAASRGICIAHEGLVPLPRANSPLLGKVQEVLHQVNQSSVQVVLLFASARAAHALFSYSISSKLSRKVWVAS   300
Gelada baboon                     VVTGKFFGFFLMPQVSYGASMELLSARETFPSFFRTVPSDRVQLTAAAELLQEFGWNWVAALGSDDEYGRQGLSIFSALAAARGICIAHEGLVPLPRANSPLLGKVQEVLHQVNQSSVQVVLLFASPRAAHALFSYSISSKLSRKVWVAS   300
Hamadryas baboon                  MVTGKFFSFFLMPQVSYGASMELLSARETFPSFFRTVPSDRVQLTAAAELLQEFGWNWVAALGSDDEYGRQGLSIFSALAAARGICIAHEGLVPLPRANSPLLGKVQEVLHQVNQSSVQVVLLFASARAAHALFSYSISSKLSRKVWVAS   300
Mandrill                          VVTGKFFGFFLMPQVSYGASMELLSARETFPSFFRTVPSDRVQLTAAAELLQEFGWNWVAALGSDDEYGRQGLSIFSALAAARGICIAHEGLVPLPRANSPLLGKVQEVLHQVNQSSVQVVLLFASARAAHALFSYSISSKLSRKVWVAS   300
Drill                             VVTGKFFGFFLMPQVSYGASMELLSARETFPSFFRTVPSDRVQLTAAAELLQEFGWNWVAALGSDDEYGRQGLSIFSALAAARGICIAHEGLVPLPRANSPLLGKVQEVLHQVNQSSVQVVLLFASARAAHALFSYSISSKLSRKVWVAS   300
Red guenon                        VVTGKFFGFFLMPQVSYGASMELLSARETFPSFFRTVPSDRVQLTAAAELLQEFGWNWVAALGSDDEYGRQGLSIFSALAAARGICIAHEGLVPLPRANSLLLGKVQEVLHQVNQSSVQVVLLFASARAAHALFSYSISSKLSRKVWVAS   300
Ma's night monkey                 LVTGKFFGFFLMPQVSYGASMDLLSTRETFPSFFRTVPSDRVQLTAAVELLQRLGWNWVAALGSDDEYGRQGLSIFSGLAAARGICIAHEGLVPLPRADSPWAGKVQELLPQVNQSGVQVVLLFASARAAHAFFSYSISRRLLPKVWVAS   300
White-tufted-ear marmoset         LVTGKFFGFFLMPQVSYGASMDLLSTRETFPSFFRTVPSDRVQLMATVELLQQLGWNWVAALGSDDEYGRQGLSIFSGLAAARGICIAHEGLVPLPRADGLWVGKVQELLPQVNQSSIQVVLLFASAHAAHAFFRHSINRRLLPKVWVAS   300
Panamanian white-faced capuchin   LVTGKFFGFFLMPQVSYGASMDLLSTRETFPSFFRTVPSDRVQLVATVELLQQLGWNWVAALGSDDEYGRQGLSIFSGLAAARGICIAHEGLVPLPHADSPWVSKVQELLPQVNQSSIQVVLLFASARAAQAFFSYGISRRLSPKVWVAS   300
Brown-capped capuchin             LVTGKFFGFFLMPQVSYGASMDLLSTRETFPSFFRTVPSDRVQLVATVELLQQLGWNWVAALGSDDEYGRQGLSIFSGLAAARGICIAHEGLVPLPHADSPWVSKVQELLPQVNQSSIQVVLLFASVRAAQAFFSYGISRRLSPKVWVAS   300
Bolivian squirrel monkey          LVTGKFFGFFLMPQVSYGASMDLLSTRETFPSFFRTVPSDRVQLVATVELLQQLGWNWVAALGSDDEYGRQGLSIFSGLAAARGICIAHEGLVPLPRANSPWVGKVQELLPQLNQTSIQVVLLFASARAAHTFFSHIISRRLSPKVWVAS   300
Common squirrel monkey            LVTGKFFGFFLMPQVSYGASMDLLSTRETFPSFFRTVPSDRVQLVATVELLQQLGWNWVAALGSDDEYGRQGLSIFSGLAAARGICIAHEGLVPLPRANSPWVGKVQELLPQLNQTSIQVVLLFASARAAHTFFSHIISRRLSPKVWVAS   300
Coquerel's sifaka                 LVTGKFFSFFLMPQVSYGASMDRLSTRETFPSFFRTVPSDRVQLTAMVELLQEFGWNWVAALGSDDEYGRQGLSIFSSLANAQGICIAHEGLVPLPHADGPQLGKVQDVLHQVNQSNVQVVVLFASVHAAYNLFSYSIRYKLSPKVWVAS   300
Gray mouse lemur                  LVTGKFFSFFLVPQVSYGASMDRLSTRETFPSFFRTVPSDRVQLTATVELLQEFGWNWVAALGSDDEYGRQGLSIFSGLANAQGICIAHEGLVPLSHTDRQQLGKVQDILHQVNQSNVQVVVLFASAHAASALFTYSIRYKLSPKVWVAS   300
Ring-tailed lemur                 LVTGKFFSFFLVPQVSYGASMDRLSTRETFPSFFRTVPSDRVQLTAMVELLQEFGWNWVAALGSDDEYGRQGLSIFSGLANAQGICIAHEGLVPLSQADGLQLGKVQDVLHQVNQSNVQVVVLFATAHAAYALFTYSISCKLSPKVWVAS   300
```

```
                                *.****: :*.*.**:::******:::.  * :*.:**:* * **::*.**::*.  :  ***.*** ****** ::***:.: *  :: *..*****.***.**.:* *.:**** ::**:**::*.****::*:. .   :*: .***
Human                           EAWLTSDLVMGLPGMAQMGTVLGFLQRGAQLHEFPQYVKTHLALATDPAFCSALGEREQGLEEDVVGQRCPQCDCITLQNVSAGLNHHQTFSVYAAVYSVAQALHNTLQCNASGCPAQDPVKPWQLLENMYNLTFHVGGLPLRFDSSGNV   450
Western lowland gorilla         EAWLTSDLVMGLPGMAQMGTVLGFLQRGAQLHEFPQYVKTHLALAADPAFCSALGEREQGLEEDVVGQRCPQCDCITLQNVSAGLNHHQTFSVYAAVYSVAQALHNTLQCNASGCPAQDPVKPWQLLENMYNLTFHAGGLMLRFDSSGNV   450
Sumatran orangutan              EAWLTSDLIMGLPGMAQVGTVLGFLQKGAQLHEFSQYVKTHLALAADPAFCAALGEREQGLEEDVVGQRCPQCDCITLQNVSAGLNHHQMFSVYAAVYSVAQALHNTLQCNASGCPAQDPVKPWQLLENMYNLTFHVGGLTLRFNSSGNV   450
Bornean orangutan               EAWLTSDLIMGLPGMAQVGTVLGFLQKGAQLHEFSQYVKTHLALAADPAFCAALGEREQGLEEDVVGQRCPQCDCITLQNVSAGLNHHQMFSVYAAVYSVAQALHNTLQCNASGCPAQDPVKPWQLLENMYNLTFHVGGLTLRFNSSGNV   450
Pygmy chimpanzee                EAWLTSDLVMGLPGMAQMGTVLGFLQRGAQLHEFPQYVKTHLALAADPAFCSSLGEREQGLEEDVVGQRCPQCDCITLQNVSAGLNHHQTFSVYAAVYSVAQALHNTLQCNASGCPAQDPVKPWQLLENMYNLTFHAGGLMMRFDSSGNV   450
Common chimpanzee                EAWLTSDLVMGLPGMAQMGTVLGFLQRGAQLHEFPQYVKTHLALAADPAFCSALGEREQGLEEDVVGQRCPQCDCITLQNVSAGLNHHQTFSVYAAVYSVAQALHNTLQCNASGCPAQDPVKPWQLLENMYNLTFHAGGLMLRFDSSGNV   450
Northern white-cheeked gibbon   EAWLTSDLVMGLPGMAQVGTVLGFLQRGAQLHEFSQYVKTHLALAADPAFCATLGKREQVLEEDVVGQRCPQCDCITLQNVSAGLNHHQTFSVYAAVYSVAQALHNTLQCNASGCPTQDPVKPWQLLENMYNLTFHAGGLTLRFNSSGNV   450
Northern buffed-cheeked gibbon  EAWLTSDLVMGLPGMAQVGTVLGFLQRGAQLHEFSQYVKTHLALAADPAFCATLGKREQVLEEDVVGQRCPQCDCITLQNVSAGLNHHQTFSVYAAVYSVAQALHNTLQCNASGCPTQDPVKPWQLLENMYNLTFHAGGLTLRFNSSGNV   450
Western hoolock gibbon          EAWLTSDLVMGLPGMAQVGTVLGFLQRGAQLHEFSQYVKTHLALAADPAFCATLGKREQVLEEDVVGQRCPQCDCITLQNVSAGLNHHQTFSVYAAVYSVAQALHNTLQCNASGCPTQDPVKPWQLLENMYNLTFHAGGLTLRFNSSGNV   450
Hylobates muelleri abbotti      EAWLTSDLVMGLPGMAQVGTVLGFLQRGAQLHEFSQYVKTHLALAADPAFCATLGKREQVLEEDVVGQRCPQCDCITLQNVSAGLNHHQTFSVYAAVYSVAQALHNTLQCNASGCPTQDPVKPWQLLENMYNLTFHAGGLTLRFNSSGNV   450
Agile gibbon                    EAWLTSDLVMGLPGMAQVGTVLGFLQRGAQLHEFSQYVKTHLALAADPAFCATLGKREQVLEEDVVGQRCPQCDCITLQNVSAGLNHHQTFSVYAAVYSVAQALHNTLQCNASGCPTQDPVKPWQLLENMYNLTFHAGGLTLRFNSSGNV   450
Common gibbon                   EAWLTSDLVMGLPGMAQVGTVLGFLQRGAQLHEFSQYVKTHLALAADPAFCATLGKREQVLEEDVVGQRCPQCDCITLQNVSAGLNHHQTFSVYAAVYSVAQALHNTLQCNASGCPTQDPVKPWQLLENMYNLTFHAGGLTLRFNSSGNV   450
Pileated gibbon                 EAWLTSDLVMGLPGMAQVGTVLGFLQRGAQLHEFSQYVKTHLALAADPAFCATLGKREQVLEEDVVGQRCPQCDCITLQNVSAGLNHHQTFSVYAAVYSVAQALHNTLQCNASGCPTQDPVKPWQLLENMYNLTFHAGGLTLRFNSSGNV   450
Siamang                         EAWLTSDLVMGLPGMAQVGTVLGFLQRGAQLHEFSQYVKTHLALAADPAFCATLGKREQVLEEDVVGQRCPQCDCITLQNVSTGLNHHQTFSVYAAVYSVAQALHNTLQCNASGCPTQDPVKPWQLLENMYNLTFHAGGLTLRFNSSGNV   450
Green monkey                    EAWLTSDLVMGLPGMAQVGTVLGFLQRGAQLHKFSQYVKTRLALAADPVFCTALGEREQGLEEDVVGRRCPQCDCITLQNVSAGLNHHQTFSVYAAVYSVAQALHNTLQCNASGCPMRDPVKPWQLLENMYNLTFHAGGLTLRFNSNGNV   450
Golden-bellied mangabey         EAWLTSDLVMGLPGMAQVGTVLGFLQRGAQLHKFSQYVKTRLALAADPAFCAALGEREQGLEEDVVGRRCPQCDCITLQNVSAGLNHHQTFSVYAAVYSVAQALHNTLHCNASGCPVQEPVKPWQLLDNMYNLTFHAGGLTLRFNSNGNV   450
Sooty mangabey                  EAWLTSDLVMGLPGMAQVGTVLGFLQRGAQLHKFSQYVKTRLALAADPAFCAALGEREQGLEEDVVGRRCPQCDCITLQNVSAGLNHHQTFSVYAAVYSVAQALHNTLHCNASGCPVQEPVKPWQLLDNMYNLTFHAGGLTLRFNSNGNV   450
Blue monkey                     EAWLTSDLVMGLPGMAQVGTVLGFLQRGAQLHKFSQYVKTRLALAADPVFCAALGEREQGLEEDVVGRRCPQCDCITLQNVSAGLNHHQTFSVYAAVYSVAQALHNTLQCNASGCPMRDPVKPWQLLENMYNLTFHAGGLMLRFNSNGNV   450
Sykes' monkey                   EAWLTSDLVMGLPGMAQVGTVLGFLQRGAQLHKFSQYVKTRLALAADPVFCAALGEREQGLEEDVVGRRCPQCDCITLQNVSAGLNHHQTFSVYAAVYSVAQALHNTLQCNASGCPMRDPVKPWQLLENMYNLTFHAGGLMLRFNSNGNV   450
Assam macaque                   EAWLTSDLVMGLPGMAQVGTVLGFLQRGAQLHKFSQYVKTRLALAADPAFCAALGEREQGLEEDVVGRRCPQCDCITLQNVSAGLNHHQTFSVYAAVYSVAQALHNALQCSASGCPVQDPVKPWQLLENMYNLTFHAGGLTLRFNSNGNV   450
Stump-tailed macaque            EAWLTSDLVMGLPGMAQVGTVLGFLQRGAQLHKFSQYVKTRLALAADPAFCAALGEREQGLEEDVVGRRCPQCDCITLQNVSAGLNHHQTFSVYAAVYSVAQALHNALQCSASGCPVQDPVKPWQLLENMYNLTFHAGGLTLRFNSNGNV   450
Japanese macaque                EAWLTSDLVMGLPGMAQVGTVLGFLQRGAQLHKFSQYVKTRLALAADPAFCAALGEREQGLEEDVVGRRCPQCDCITLQNVSAGLNHHQTFSVYAAVYSVAQALHNALQCSASGCPVQDPVKPWQLLENMYNLTFHAGGLTLRFNSNGNV   450
Crab-eating macaque             EAWLTSDLVMGLPGMAQVGTVLGFLQRGAQLHKFSQYVKTRLALAADPAFCAALGEREQGLEEDVVGRRCPQCDCITLQNVSAGLNHHQTFSVYAAVYSVAQALHNALQCSASGCPVQDPVKPWQLLENMYNLTFHAGGLTLRFNSNGNV   450
Pig-tailed macaque              EAWLTSDLVMGLPGMAQVGTVLGFLQRGAQLHKFSQYVKTRLALAADPAFCAALGEREQGLEEDVVGRRCPQCDCITLQNVSAGLNHHQTFSVYAAVYSVAQALHNALRCSASGCPVQDPVKPWQLLENMYNLTFHAGGLTLRFNSNGNV   450
Rhesus macaque                  EAWLTSDLVMGLPGMAQVGTVLGFLQRGAQLRKFSQYVKTRLALAADPAFCAALGEREQGLEEDVVGRRCPQCDCITLQNVSAGLNHHQTFSVYAAVYSVAQALHNALQCSASGCPVQDPVKPWQLLENMYNLTFHAGGLTLRFNSNGNV   450
Gelada baboon                   EAWLTSDLVMGLPGMAQVGTVLGFLQRGAQLHKFSQYVKTRLALAADPVFCAALGEREQGLEEDVVGRRCPQCDCITLQNVSAGLNHHQTFSVYAAVYSVAQALHNTLQCNASGCPVQDPVKPWQLLDNMYNLTFHAGGLTLRFNSNGNV   450
Hamadryas baboon                EAWLTSDLVMGLPGMAQVGTVLGFLQRGAQLHKFSQYVKTRLALAADPVFCATLGEREQGLEEDVVGRRCPQCDCITLQNVSAGLNHHQTFSVYAAVYSVAQALHNTLQCNASGCPVQDPVKPWQLLDNMYNLTFHAGGLTPRFNSSGNV   450
Mandrill                        EAWLTSDLVMGLPGMAQVGTVLGFLQRGAQLHKFSQYVKTRLALAADPAFCAALGEREQGLEEDVVGRRCPQCDCITLQNVSAGLNHHQTFSVYAAVYSVAQALHNTLHCNASGCPVQEPVKPWQLLDNMYNLTFHAGGLTLRFNSNGNV   450
Drill                           EAWLTSDLVMGLPGMAQVGTVLGFLQRGAQLHKFSQYVKTRLALAADPAFCAALGEREQGLEEDVVGRRCPQCDCITLQNVSAGLNHHQTFSVYAAVYSVAQALHNTLHCNASGCPVQEPVKPWQLLDNMYNLTFHAGGLTLRFNSNGNV   450
Red guenon                      EAWLTSDLVMGLPGMAQVGTVLGFLQRGAQLHKFSQYVKTRLALAADPVFCAALGEREQGLEEDVVGRRCPQCDCITLQNVSAGLNHHQTFSVYAAVYSVAQALHSTLQCNASGCPMRDPVKPWQLLENMYNLTFHAGGLTLRFNSNGNV   450
Ma's night monkey               EAWLTSDLVMGLPGMAEVGTVLGFLQRGAPLPKFSQYVKTHLALAADPAFCASLGESEQGLEEHVVGPRCPQCDNVTLQNVSAGLPHHRTFSVYAAVYGVAQALHNTLRCSASGCPTQDPVKPWQLLHNMYNMTFRAAGLALRFDGSGNV   450
White-tufted-ear marmoset       EAWLTSDLVMGLPGMAEVGTVLGFLQRGAQLPKFPQYVKTHLALAADPAFCASLSEREQGLEEHVVGPRCPQCDDVTLQNMSTRLQHHQTFSVYAAVYSVAQALHNTLRCNASGCPKQDPVKPWQLLQNMYNMTFHAAGLALRFDSSGNV   450
Panamanian white-faced capuchin EAWLTSDLVMGLPGMTEVGTVLGFLQRGAQLPKFSQYVKTHLALAADPAFCASLGEREQGLEEHVVGPRCPQCDDVTLQNVSARLQHHQTFSVYAAVYSVAQGLHNTLRCNASGCPVQDPVKPWQLLQNMYNMTFRVAGLVLRFDSSGNV   450
Brown-capped capuchin           EAWLTSDLVMGLPGMAEVGTVLGFLQRGAQLPKFSQYVKTHLALAADPAFCASLGEREQGLEEHVVGPRCPQCDDVTLQNVSAKLQHHQTFSVYAAVYSVAQGLHNTLRCNASGCPMQDPVKPWQLLQNMYNMTFRVAGLVLRFDSSGNV   450
Bolivian squirrel monkey        EAWLTSDLVMGLPGMAEVGTVLGFLQRGAQLPEFSQYVKTHLALAADPAFCTSLGEREQGLEEHVVGPRCPQCDDITLQNVPARLQHHQTFSVYAAVYSVAQALHNTLGCNASGCPMQDPVKPWQVLQNMYNMTFHAAGQVLRFDSSGNV   450
Common squirrel monkey          EAWLTSDLVMGLPGMAEVGTVLGFLQKGAQLPEFSQYVKTHLALAADPAFCTSLGEREQGLEEHVVGPRCPQCDDITLQNVPARLQHHQTFSVYAAVYSVAQALHNTLGCNASGCPMQDPVKPWQVLQNMYNMTFHAAGQVLRFDSSGNV   450
Coquerel's sifaka               EAWLTSNQVMALPGMAQVGTVLGFLHRGTPLPEFSNYVQTCLALAADPAFCASLSMEQPDLEEHVVGPRCPQCDHITLQNVSAELRNHRAFAAYAAVYSVAQALHNTLQCNSSGCPAREPVQPWELLENMYNMSFHVRGLALQFDINGNV   450
Gray mouse lemur                EAWLTSERVMALPGMAQVGTVLGFLHRSLPLPKFANYVKTRLHLAANPAFCASLSAEQPGLEEHVVGPRCPQCDCISLQNVSAELANQRTFPAYAAVYSVAQALHNTLQCNSSGCPAHEPVQPWQLLENMYNMSFRAHGLTLQFDVNGNV   450
Ring-tailed lemur               EPWLTSERVMALAGMAQVGTVLGFMQRGLLLPEFSDYVKTRLALAADPAFCASLSVEQPDLEEHVVGPRCPQCDHITLQNVSAELMYHRTFPAYAAVYSVAQALHNTLQCNSSGCPAREPVQPWQLLENMYNMSFRARSLELQFDVNGNV   450
```

```
                                * *.***** *   .* *  ** *:  *    :  :: ***  *: ***:**::*:********************.****:  .**  *. * :::***.***:** *  :**.*.:**** ****:.*.******:****::* *****
Human                           DMEYDLKLWVWQGSVPRLHDVGRFNGSLR-TERLKIRWHTSDNQKPVSRCSRQCQEGQVRRVKGFHSCCYDCVDCEAGSYRQNPDDIACTFCGQDEWSPERSTRCFRRRSRFLAWGEPAVLLLLLLLSLALGLVLAALGLFVHHRDSPLV   599
Western lowland gorilla         DMEYDLKLWVWQGSVPRLHDVGRFNGSLR-TERLKIRWHTSDNQKPVSRCSRQCQEGQVRRVKGFHSCCYDCVDCEAGSYRQNPDDVTCTSCGQDEWSPERSTRCFHRRSRFLAWGEPAVLLLLLLLSLALGLVLAALGLFVHHRDSPLV   599
Sumatran orangutan              DMEYDLKLWVWQGSVPKLHNVGGFNGSLW-TERLKIRWHTPDNQKPVSQCSRQCQEGQVRRVKGFHSCCYDCVDCKAGSYRHSPDDLACTFCRQDEWSPERSTRCFRRRSRFLAWGEPAVLLLLLLLSLALGLVLAALGLFIRHRDSPLV   599
Bornean orangutan               DMEYDLKLWVWQGSVPKLHNVGGFNGSLW-TERLKIRWHTPDNQKPVSQCSRQCQEGQVRRVKGFHSCCYDCVDCKAGSYRHSPDDLACTFCRQDEWSPERSTRCFRRRSRFLAWGEPAVLLLLLLLSLALGLVLAALGLFIRHRDSPLV   599
Pygmy chimpanzee                DMEYDLKLWVWQGSVPRLHDVGRFNGSLR-TERLKIHWHTSDNQKPVSRCSRQCQEGQVRRVKGFHSCCYDCVDCEAGSYRQNPDDIACTFCGQDEWSPERSTRCFRRRSRFLVWGEPAVLLLLLLLSLALGLVLAALGLFIHHRDSPLV   599
Common chimpanzee               DMEYDLKLWVWQGSVPRLHDVGRFNGSLR-TERLKIRWHTSDNQKPVSRCSRQCQEGQVRRVKGFHSCCYDCVDCEAGSYRQNPDDIACTFCGQDEWSPERSTRCFRRRSRFLAWGEPAVLLLLLLLSLALGLVLAALGLFIHHRDSPLV   599
Northern white-cheeked gibbon   DMEYDLKLWVWQGSVPKLHNVGRFNGSLW-TEHLKIRWHTLDNQKPVSQCSQQCQEGQVRRVKGFHSCCYDCVDCKAGSYRHSPDDLACTFCRQDEWSPERSTRCFRRRYRFLAWGEPAVLLLLLLLSLALGLVLAALGLFIRHRDSPLV   599
Northern buffed-cheeked gibbon  DMEYDLKLWVWQGSVPKLHNVGRFNGSLW-TEHLKIRWHTLDNQKPVSQCSQQCQEGQVRRVKGFHSCCYDCVDCKAGSYRHSPDDLACTFCRQDEWSPERSTRCFRRRYRFLAWGEPAVLLLLLLLSLALGLVLAALGLFIRHRDSPLV   599
Western hoolock gibbon          DMEYDLKLWVWQGSVPKLHNVGRFNGSLW-TEHLKIRWHTLDNQKPVSQCSQQCQEGQVRRVKGFHSCCYDCVDCKAGSYRHSPDDLACTFCRQDEWSPERSTRCFRRRYRFLAWGEPAVLLLLLLLSLALGLVLAALGLFIRHRDSPLV   599
Hylobates muelleri abbotti      DMEYDLKLWVWQGSVPKLHNVGRFNGSLW-TEHLKIRWHTLDNQKPVSQCSQQCQEGQVRRVKGFHSCCYDCVDCKAGSYRHSPDDLACTFCRQDEWSPERSTRCFRRRYRFLAWGEPAVLLLLLLLSLALGLVLAALGLFIRHRDSPLV   599
Agile gibbon                    DMEYDLKLWVWQGSVPKLHNVGRFNGSLW-TEHLKIRWHTLDNQKPVSQCSQQCQEGQVRRVKGFHSCCYDCVDCKAGSYRHSPDDLACTFCRQDEWSPERSTRCFRRRYRFLAWGEPAVLLLLLLLSLALGLVLAALGLFIRHRDSPLV   599
Common gibbon                   DMEYDLKLWVWQGSVPKLHNVGRFNGSLW-TEHLKIRWHTLDNQKPVSQCSQQCQEGQVRRVKGFHSCCYDCVDCKAGSYRHSPDDLACTFCRQDEWSPERSTRCFRRRYRFLAWGEPAVLLLLLLLSLALGLVLAALGLFIRHRDSPLV   599
Pileated gibbon                 DMEYDLKLWVWQGSVPKLHNVGRFNGSLW-TEHLKIRWHTLDNQKPVSQCSQQCQEGQVRRVKGFHSCCYDCVDCKAGSYRHSPDDLACTFCRQDEWSPERSTRCFRRRYRFLAWGEPAVLLLLLLLSLALGLVLAALGLFIRHRDSPLV   599
Siamang                         DMEYDLKLWVWQGSVPKLHNVGRFNGSLW-TERLKIRWHTLDNQKPVSQCSQQCQEGQVRRVKGFHSCCYDCVDCKAGSYRHSPDDLACTFCRQDEWSPERSTRCFRRRYRFLAWGEPAVLLLLLLLSLALGLVLAALGLFIRHRDSPLV   599
Green monkey                    DMEYDLKLWVWQGPVPELHDVGRFNGSLW-IDSLKIHWHTSNNQKPVSQCSRQCQEGQVRRVKGFHSCCYDCVDCKAGSYRKSPDDLACTFCSQDEWSPERSTRCFRRRLRFLAWGEPAVLLLLLLGLALGLVLAALGLFIRHRDSPLV   599
Golden-bellied mangabey         DMEYDLKLWVWQGPVPELHNVGRFNGSLW-IDSLKIRWHTSNNQKPVSQCSRQCQEGQVRRVKGFHSCCYDCVDCKAGSYRKSPDDLACTFCSQDEWSPERSTRCFRRRLRFLAWGEPAVLLLLLLFGLALGLVLAALGLFIRHRDSPLV   599
Sooty mangabey                  DMEYDLKLWVWQGPVPELHNVGRFNGSLW-IDSLKIRWHTSNNQKPVSQCSRQCQEGQVRRVKGFHSCCYDCVDCKAGSYRKSPDDLACTFCSQDEWSPERSTRCFRRRLRFLAWGEPAVLLLLLLFGLALGLVLAALGLFIRHRDSPLV   599
Blue monkey                     DMEYDLKLWVWQGPVPELHDVGRFNGSLW-VDSLKIRWHTSNNQMPVSQCSRQCQEGQVRRVKGFHSCCYDCVDCKAGSYRKSPDDLACTFCSQEEWSPERSTRCFRRRLRFLAWGEPAVLLLLLLGLALGLVLAALGLFIRHRDSPLV   599
Sykes' monkey                   DMEYDLKLWVWQGPVPELHDVGRFNGSLW-VDSLKIRWHTSNNQMPVSQCSRQCQEGQVRRVKGFHSCCYDCVDCKAGSYRKSPDDLACTFCSQEEWSPERSTRCFRRRLRFLAWGEPAVLLLLLLGLALGLVLAALGLFIRHRDSPLV   599
Assam macaque                   DMEYDLKLWVWQGPVPELHDVGRFNGNLW-IDSPKIRWHTSNNQKPVSQCSRQCQEGQVRRVKGFHSCCYDCVDCKAGSYRKSPDDLACTFCGQEEWSPERSTRCFRRRLRFLAWGEPAVLLLLLLFGLALGLVLAALGLFIRHRDSPLV   599
Stump-tailed macaque            DMEYDLKLWVWQGPVPELHDVGRFNGSLW-IDSPKIRWHTSNNQKPVSQCSRQCQEGQVRRVKGFHSCCYDCVDCKAGSYRKSPDDLACTFCGQEEWSPERSTRCFRRRLRFLAWGEPAVLLLLLLFGLALGLVLAALGLFIRHRDSPLV   599
Japanese macaque                DMEYDLKLWVWQGPVPELHDVGRFNGSLW-IDSPKIRWHTSNNQKPVSQCSRQCQEGQVRRVKGFHSCCYDCVDCKAGSYRKSPDDFACTFCGREEWSPERSTRCFRRRLRFLAWGEPAVLLLLLLFGLALGLVLAALGLFIRHRDSPLV   599
Crab-eating macaque             DMEYDLKLWVWQGPVPELHDVGRFNGSLW-IDSPKIRWHTSNNQKPVSQCSRQCQEGQVRRVKGFHSCCYDCVDCKAGSYRKSPDDLACTFCGQEEWSPERSTRCFRRRLRFLAWGEPAVLLLLLLFGLALGLVLAALGLFIRHRDSPLV   599
Pig-tailed macaque              DMEYDLKLWVWQGPVPELHDVGRFNGSLW-IDSPKIRWHTSNNQKPVSQCSRQCQEGQVRRVKGFHSCCYDCVDCKAGSYRKSPDDLACTFCGREEWSPERSTRCFRRRLRFLAWGEPAVLLLLLLFGLALGLVLAALGLFIRHRDSPLV   599
Rhesus macaque                  DMEYDLKLWVWQGPVPELHDVGRFNGSLW-IDSPKIRWHTSNNQKPVSQCSRQCQEGQVRRVKGFHSCCYDCVDCKAGSYRKSPDDLACTFCGREEWSPERSTRCFRRRLRFLAWGEPAVLLLLLLFGLALGLVLAALGLFIRHRDSPLV   599
Gelada baboon                   DMEYDLKLWVWQGPVPELHDVGRFNGSLW-IDSLKIRWHTSNNQKPVSQCSRQCQEGQVRRVKGFHSCCYDCVDCKAGSYRKSPDDLACTFCSQDEWSPERSTRCFRRRLRFLAWGEPAVLLLLLLFGLALGLVLAALGLFIRHRDSPLV   599
Hamadryas baboon                DMEYDLKLWVWQGSVPRLHDVGRFNGSLR-TERLKIRWHTSDNQKPVSRCSRRCQEGQVRRVKGFHSCCYDCVDCEAGSYRQNPDDIACPFCGQDEWSPERSTRCFRRRSRFLAWGEPAVLLLLLLLSLALGLVLAALGLFVHHRDSPLV   599
Mandrill                        DMEYDLKLWVWQGPVPELHDVGRFNGSLW-IDSLKIRWHTSNNQKPVSQCSRQCQEGQVRRVKGFHSCCYDCVDCKAGSYRKSPDDLACTFCSQDEWSPERSTRCFRRRLRFLAWGEPAVLLLLLLFGLALGLVLAALGLFIRHRDSPLV   599
Drill                           DMEYDLKLWVWQGPVPELHDVGRFNGSLW-IDSLKIRWHTSNNQKPVSQCSRQCQEGQVRRVKGFHSCCYDCVDCKAGSYRKSPDDLACTFCSQDEWSPERSTRCFRRRLRFLAWGEPAVLLLLLLFGLALGLVLAALGLFIRHRDSPLV   599
Red guenon                      DMEYDLKLWVWQGPVPELHDVGRFNGSLW-IDSLKIHWHTSNNQKPVSQCSRQCQEGQVRRVKGFHSCCYDCVDCKAGSYRKSPDDLACTFCSQDEWSPERSTQCFRRRLRFLAWGEPAVLLLLLLLSLALGLVLAALGLFIRHRDSPLV   599
Ma's night monkey               DAEYDLKLWVWRGPVPELHNVGVFNGSLW-PERLKMRWHTPGNQEPVSQCSRQCQEGQVRRVKGFHSCCYDCVDCEAGSYRRNPDDPACTPCRHDQWSPERSTRCFRRRPRFLTWGEPAVLLLLLLLGLALGLVLAALGLFVRHRDSPLV   599
White-tufted-ear marmoset       DVEYDLKLWVWRGPVPELHDVGVFNGSLW-PERLKMRWHTPDNQEPVSQCSRQCQEGQVRRVKGFHSCCYDCVDCEAGSYRRNPDDPTCTPCRHDQWSPERSTRCFRRRPRFLTWSEPAVLLLLLLLGLALGLVLAALGLFIHHRDSPLV   599
Panamanian white-faced capuchin DMEYDLKLWVWRGPVPELHNVGIFNGSLW-PERLKMRWHTPDNQEPVSQCSRQCQEGQVRRVKGFHSCCYDCVDCEAGSYRRNPDDPTCTPCRHDQWSPERSTRCFRRRPRFLTWGEPAVLLLLLLLGLALGLVLAALGLFIRHRDSPLV   599
Brown-capped capuchin           DMEYDLKLWVWRGPVPELHDVGIFNGSLW-PERLKMRWHTPDNQEPVSQCSRQCQEGQVRRVKGFHSCCYDCVDCEAGSYRRNPDDPTCTPCRHDQWSPERSTRCFRRRPRFLTWGEPAVLLLLLLLGLALGLVLAALGLFIRHRDSPLV   599
Bolivian squirrel monkey        DVEYDLKLWVWRGPVPELHNVGIFNGSLW-PERLKMRWHTPDNQEPVSQCSRQCQEGQVRRVKGFHSCCYDCVDCEAGSYRRNPDDPTCTPCRHDQWSPKRSTRCFHRRPRFLTWGEPAVLLLLLLLGLALGLVLATLGLFIRHRDSPLV   599
Common squirrel monkey          DVEYDLKLWVWRGPVPELHNVGIFNGSLW-PERLKMRWHTPDNQEPVSQCSRQCQEGQVRRVKGFHSCCYDCVDCEAGSYRRNPDDPTCTPCRHDQWSPKRSTRCFHRRPRFLTWGEPAVLLLLLLLGLALGLVLATLGLFIRHRDSPLV   599
Coquerel's sifaka               DMEHDLKLWVWGQRTPTLLTVGTFHKHLQ-LQHGKISWHTAHNQTPVSQCSRQCREGQVRRVKGFHSCCYDCVDCKAGSYQHSADDLTCTPCNQDEWSPERSTRCFRRTTKFLAWGEPAVLLLLLLLGLVLGLVLAALGLFVHHLDSPLV   599
Gray mouse lemur                DMEYDLKLWEWKEPTPMLRTVGTFHRRLHLLQHSQIVWHTAHNEVPVSQCSRQCREGQVRRVKGFHSCCYDCVDCKAGSYQHRADDFICTTCNHDEWSPDRSTRCFPRTTRFLAWGDPAVLGLLLLLALVLGLVLAALGLFVHHQDSPLV   600
Ring-tailed lemur               DMEFDLKLWVWEQQTPKLLTVGTFHRHLQ-VQHGKILWHTAHNQTPVSQCSRQCREGQVRRVKGFHSCCYDCVDCRAGSYQSSTDDLTCTLCNRDEWSPERSTRCFPRRTRFLAWGDPAVLLLLLLLGLVLGLVLAALGLFVHHQDSPLV   599
```

```
                                :**** ****.*.******:. *****:*..: **.*** ::*********::****: * ***** ***  : * *:*. ***.**:.:***.***:*** .*** :****::**.***.**  :***.:**.*::*: ** *.*****
Human                           QASGGPLACFGLVCLGLVCLSVLLFPGQPSPARCLAQQPLSHLPLTGCLSTLFLQAAEIFVESELPLSWADRLSGCLRGPWAWLVVLLAMLVEVALCTWYLVAFPPEVVTDWHMLPTEALVHCRTRSWVSFGLAHATNATLAFLCFLGTF   749
Western lowland gorilla         QASGGPLACFGLVCLGLVCLSVLLFPGQPSPAQCLAQQPLSHLPLTGCLSTLFLQAAEIFVESELPLSWADRLSGCLRGPWAWLVVLLAMLVEVALCTWYLVAFPPEVVTDWHMLPTEALVHCRTRSWVSFGLAHATNATLAFLCFLGTF   749
Sumatran orangutan              RASGGPLACFGLVCLGLVCLSVLLFPGRPGTARCLAQQPLSHLPLTGCLSTLFLQAAEIFVESELPLSWADRLSGCLRGPWAWLVVLLAMLVEVALCTWYLVAFPPEVVTDWHILPTEALVHCRTRSWVSFGLAHATNATLAFLCFLGTF   749
Bornean orangutan               RASGGPLACFGLVCLGLVCLSVLLFPGRPGTARCLAQQPLSHLPLTGCLSTLFLQAAEIFVESELPLSWADRLSGCLRGPWAWLVVLLAMLVEVALCTWYLVAFPPEVVTDWHILPTEALVHCRTRSWVSFGLAHATNATLAFLCFLGTF   749
Pygmy chimpanzee                QASGGPLACFGLVCLGLVCLSVLLFPGQPSPARCLAQQPLSHLPLTGCLSTLFLQAAEIFVESELPLSWADRLSGCLRGPWAWLVVLLAMLVEVALCTWYLVAFPPEVVTDWHMLPTEALVHCRTRSWVSFGLAHATNATLAFLCFLGTF   749
Common chimpanzee               QASGGPLACFGLVCLGLVCLSVLLFPGQPSPARCLAQQPLSHLPLTGCLSTLFLQAAEIFVESELPLSWADRLSGCLRGPWAWLVVLLAMLVEVALCTWYLVAFPPEVVTDWHMLPTEALVHCRTRSWVSFGLAHATNATLAFLCFLGTF   749
Northern white-cheeked gibbon   QASGGPLACFGLVCLGLVCLSVLLFPGQPSPARCLAQQPLSHLPLTGCLSTLFLQAAEIFVESELPLSWADRLSGCLRGPWAWLVVLLAMLVEAALCTWYLVAFPPEVVTDWRMLPKEALVHCRTRSWVSFGLAHATNATLAFLCFLGTF   749
Northern buffed-cheeked gibbon  QASGGPLACFGLVCLGLVCLSVLLFPGQPSPARCLAQQPLSHLPLTGCLSTLFLQAAEIFVESELPLSWADRLSGCLRGPWAWLVVLLAMLVEAALCTWYLVAFPPEVVTDWRMLPKEALVHCRTRSWVSFGLAHATNATLAFLCFLGTF   749
Western hoolock gibbon          QASGGPLACFGLVCLGLVCLSVLLFPGQPSPARCLAQQPLSHLPLTGCLSTLFLQAAEIFVESELPLSWADRLSGCLRGPWAWLVVLLAMLVEAALCTWYLVAFPPEVVTDWRMLPKEALVHCRTRSWVSFGLAHATNATLAFLCFLGTF   749
Hylobates muelleri abbotti      QASGGPLACFGLVCLGLVCLSVLLFPGQPSPARCLAQQPLSHLPLTGCLSTLFLQAAEIFVESELPLSWADRLSGCLRGPWAWLVVLLAMLVEAALCTWYLVAFPPEVVTDWRMLPKEALVHCRTRSWVSFGLAHATNATLAFLCFLGTF   749
Agile gibbon                    QASGGPLACFGLVCLGLVCLSVLLFPGQPSPARCLAQQPLSHLPLTGCLSTLFLQAAEIFVESELPLSWADRLSGCLRGPWAWLVVLLAMLVEAALCTWYLVAFPPEVVTDWRMLPKEALVHCRTRSWVSFGLAHATNATLAFLCFLGTF   749
Common gibbon                   QASGGPLACFGLVCLGLVCLSVLLFPGQPSPARCLAQQPLSHLPLTGCLSTLFLQAAEIFVESELPLSWADRLSGCLRGPWAWLVVLLAMLVEAALCTWYLVAFPPEVVTDWRMLPKEALVHCRTRSWVSFGLAHATNATLAFLCFLGTF   749
Pileated gibbon                 QASGGPLACFGLVCLGLVCLSVLLFPGQPSPARCLAQQPLSHLPLTGCLSTLFLQAAEIFVESELPLSWADRLSGCLRGPWAWLVVLLAMLVEAALCTWYLVAFPPEVVTDWRMLPKEALVHCRTRSWVSFGLAHATNATLAFLCFLGTF   749
Siamang                         QASGGPLACFGLVCLGLVCLSVLLFPGQPSPARCLAQQPLSHLPLTGCLSTLFLQAAEIFVESELPLSWADRLSGCLRGPGAWLVVLLAMLVEAALCTWYLVAFPPEVVTDWRMLPKEALVHCRTRSWVSFGLAHATNATLAFLCFLGTF   749
Green monkey                    QASGGPLACFGLVCLGLVCISVLLFPGQPSPARCLAQQPSSHLPLTGCLSTLILQAAEIFVESELPLSWADRLSGCLRGPWAWLVVLLAVLVEAALCAWYLMVFPPEVVTDWRMLPTEALVHCRARSWVSFGLVHATNATLAFLCFLGTF   749
Golden-bellied mangabey         QASGGPLACFGLVCLGLVCISVLLFPGQPSPARCLAQQPSSHLPLTGCLSTFILQAAEIFMESELPLSWADRLSGCLRGPWAWLVVLLAMLVEAALCAWYLVAFPPEVVTDWRMLPTEALVHCRARSWVSFGLVHATNATLAFLCFLGTF   749
Sooty mangabey                  QASGGPLACFGLVCLGLVCISVLLFPGQPSPARCLAQQPSSHLPLTGCLSTFILQAAEIFMESELPLSWADRLSGCLRGPWAWLVVLLAMLVEAALCAWYLVAFPPEVVTDWRMLPTEALVHCRARSWVSFGLVHATNATLAFLCFLGTF   749
Blue monkey                     QASGGPLACFGLVCLGLVCLSVLLFPGQPSPARCLAQQPSSHLPLTGCLSTLILQAAEIFVESELPLSWADRLSGCLRGPGAWLVVLLAMLVEAALCAWYLVAFPPEVVTDWRMLPTEALVHCRARSWVSFGLVHATNATLAFLCFLGTF   749
Sykes' monkey                   QASGGPLACFGLVCLGLVCLSVLLFPGQPSPARCLAQQPSSHLPLTGCLSTLILQAAEIFVESELPLSWADRLSGCLRGPGAWLVVLLAMLVEAALCAWYLVAFPPEVVTDWRMLPTEALVHCRARSWVSFGLVHATNATLAFLCFLGTF   749
Assam macaque                   QASGGPLACFGLVCLGLVCISVLLFPGQPSPARCLAQQPSSHLPLTGCLSTFILQAAEIFAESELPLSWADRLSGCLRGPWAWLVVLLAMLVEAALCAWYLVAFPPEVVTDWRMLPTEALVHCRTRSWVSFGLVHATNATLAFLCFLGTF   749
Stump-tailed macaque            QASGGPLACFGLVCLGLVCISVLLFPGQPSPARCLAQQPSSHLPLTGCLSTFILQAAEIFAESELPLSWADRLSGCLRGPWAWLVVLLAMLVEAALCAWYLVAFPPEVVTDWRMLPTEALVHCRTRSWVSFGLVHATNATLAFLCFLGTF   749
Japanese macaque                QASGGPLACFGLVCLGLVCISVLLFPGQPSPARCLAQQPSSHLPLTGCLSTFILQAAEIFVESELPLSWADRLSGCLRGPWAWLVVLLAMLVEAALCAWYLVAFPPEVVTDWRMLPTEALVHCRTRSWVSFGLVHATNTTLAFLCFLGTF   749
Crab-eating macaque             QASGGPLACFGLVCLGLVCISVLLFPGQPSPARCLAQQPSSHLPLTGCLSTFILQAAEIFAESELPLSWADRLSGCLRGPWAWLVVLLAMLVEAALCAWYLVAFPPEVVTDWRMLPTEALVHCRTRSWVSFGLVHATNATLAFLCFLGTF   749
Pig-tailed macaque              QASGGPLACFGLVCLGLVCISVLLFPGQPSPARCLAQQPSSHLPLTGCLSTFILQAAEIFAESELPLSWADRLSGCLRGPWAWLVVLLAMLVEAALCAWYLVAFPPEVVTDWRMLPTEALAHCRTRSWVSFGLVHATNATLAFLCFLGTF   749
Rhesus macaque                  QASGGPLACFGLVCLGLVCISVLLFPGQPSPARCLAQQPSSHLPLTGCLSTFILQAAEIFVESELPLSWADRLSGCLRGPWAWLVVLLAMLVEAALCAWYLVAFPPEVVTDWRMLPTEALVHCRTRSWVSFGLVHATNATLAFLCFLGTF   749
Gelada baboon                   QASGGLLACFSLVCLGLVCISVLLFPGQPSPARCLAQQPSSHLPLTGCLSTFILQAAEIFVESELPLSWADRLSGCLRGPWAWLVVLVAMLVEAALCAWYLVAFPPEMVTDWRMLPTEALVHCRARSWVSFGLVHATNATLAFLCFLGTF   749
Hamadryas baboon                QASGGPLACFGLVCLGLVCLSVLLFPGQPSPARCLAQQPLSHLPLTGCLSTLFLQAAEIFVESELPLSWADRLSGCLRGPWAWLVVLLAMLVEVALCTWYLVAFPPEVVTDWHMLPTEALVHCRTRSWVSFGLVHATNATLAFLCFLGTF   749
Mandrill                        QASGGPLACFGLVCLGLVCISVLLFPGQPSPARCLAQQPSSHLPLTGCLSTFILQAAEIFVESELPLSWADRLSGCLRGPWAWLVVLLAMLVEAALCAWYLVAFPPEVVTDWRMLPTEALVHCRARSWVSFGLVHATNATLAFLCFLGTF   749
Drill                           QASGGPLACFGLVCLGLVCISVLLFPGQPSPARCLAQQPSSHLPLTGCLSTFILQAAEIFVESELPLSWADRLSGCLRGPWAWLVVLLAMLVEAALCAWYLVAFPPEVVTDWRMLPTEALVHCRARSWVSFGLVHATNATLAFLCFLGTF   749
Red guenon                      QASGGPLACFGLVCLGLVCISVLLFPGQPSPARCLAQQPSSHLPLTGCLSTLILQAAEIFVESELPLSWADRLSGCLRGPWAWLVVLLAMLVEAALCTWYLMAFPPEVVTDWRMLPTEALVHCRARSWVSFGLVHATNATLAFLCFLGTF   749
Ma's night monkey               QASGGALACFGLVCLGLVCLSVLLFPGQPSPARCLAQQPLSQLPLTGCLSTLFLQAAETFVESELPQSWADRLRGCLRGPRAWLAVLLAMLVEAALCAWYLLAFPPEVVTDWRVLPTEALVHCRTRSWVSFGLVHATNAILAFLCFLGTF   749
White-tufted-ear marmoset       QASGGGLACFGLVCLGLVCLSVLLFPGQPSPARCLAQQPLSHLPLTGCLSTLFLQAAETFVESELPQSWADRVRGCLRGSRAWLAVLLAMLVEAALCAWYLLAFPPEVVTDWRVLPTEALVHCCTRSWVSFGLVHATNAILAFLSFLGTF   749
Panamanian white-faced capuchin QASGGALACFGLVCLGLVCLSVLLFPGQPSPARCLVQQPLSHLPLTGCLSTLFLQAAETFVESELPQSWADRLRGCLQGPWAWLAVLLAMLVEAALCAWYLLAFPPEVVTDWRVLPTEALVHCRTRSWVSFGLVHATNAILAFLCFLGTF   749
Brown-capped capuchin           QASGGALACFGLVCLGLVCLSVLLFPGQPSPARCLAQQPLSHLPLTGCLSTLFLQAAETFVESELPQSWADRLRGCLQGPWAWLVVLLAMLVEAALCAWYLLAFPPEVVTDWRVLPTEALVHCRTRSWVSFGLVHATNAILAFLCFLGTF   749
Bolivian squirrel monkey        QASGGALACFGLVCLGLVCLSVLLFPGQPSPARCLAQQPLSHLPLTGCLSTLFLQAAETFVESELPPSWADRLWGCLRGPRAWLAVLLAMLVEAALCAWYLLAFPPEVVTDWRVLPTEALVHCRTRSWVSFGLVHTTNAILAFLCFLGTF   749
Common squirrel monkey          QASGGALACFGLVCLGLVCLSVLLFPGQPSPARCLAQQPLSHLPLTGCLSTLFLQAAETFVESELPPSWADRLWGCLRGPRAWLAVLLAMLVEAALCAWYLLTFPPEVVTDWRVLPTEALVHCRTRSWVSFGLVHTTNAILAFLCFLGTF   749
Coquerel's sifaka               QASGGLLACFGLACLGLVCLGSLLFPGRPSPASCLAQQPLSHLPLTGCLSTLFLQAAKTFVESELPPSWADWLRGRLQGPWAWLAVLLAVLVEAALCTWYLTAFPPEVVTDWQVLPTEALVHCRMHSWVSLGLVHATNATLASLCFLGTF   749
Gray mouse lemur                QASGGLLACFGLACLGLVCLGSLLFPGRPSPASCLAQQPLSHLPLTGCLSTLFLQAAEIFVESELPPSWAAWLRGHLQGPWAWLVVLLAILVEAALCAWYLTAFPPEVVTDWQVLPTEALVHCRVHSWVGLGLVHAANAALASLCFLGTF   750
Ring-tailed lemur               QASGGLLACFGLACLGLVCLSSLLFPGRPSPTSCLAQQPLAHLPLTGCLSTLLLQAAEIFVESELPQSWADWLRGRLQGPWAWLAVLLPILVEAALCTWYLTAFPPAVVTDWQVLPTEALVHCRVHSWVSLGLVHATNATLASLCFLGTF   749
```

```
                                    **:*:*  *.:***:*** ***:* **********.:*. :**::***:**..****.* ***:** *: ** ****:**** .      .          : :
Human                               LVRSQPGCYNRARGLTFAMLAYFITWVSFVPLLANVQVVLRPAVQMGALLLCVLGILAAFHLPRCYLLMRQPGLNTPEFFLGGGPGDAQGQN--DGNTGNQGKHE   852
Western lowland gorilla             LVRSQPGRYNRARGLTFAMLAYFITWVSFVPLLANVQVVLRPAVQMGALLLCVLGILAAFHLPRCYLLIRQPGLNTPEFFLGGGPGDAQGRN--DGDTGNQGKHE   852
Sumatran orangutan                  LVQSRPGRYNRARGLTFAMLAYFITWVSFVPLLANVQVVLRPAVQMGALLLCVLGILAAFHLPRCYLLMRQPGLNTPEFFLGGGPGDAQGRN--DGDTGNQRKHE   852
Bornean orangutan                   LVQSRPGRYNRARGLTFAMLAYFITWVSFVPLLANVQVVLRPAVQMGALLLCVLGILAAFHLPRCYLLMRQPGLNTPEFFLGGGPGDAQGRN--DGDTGNQRKHE   852
Pygmy chimpanzee                    LVRSQPGRYNRARGLTFAMLAYFITWVSFVPLLANVQVVLRPAVQMGALLLCVLGILAAFHLPRCYLLMWQPGLNTPEFFLGGGPGDAQGRN--DGDTGNQGKHE   852
Common chimpanzee                   LVRSQPGRYNRARGLTFAMLAYFITWVSFVPLLANVQVVLRPAVQMGALLLCVLGILAAFHLPRCYLLMWQPGLNTPEFFLGGGPGDAQGRN--DGDTGNQGKHE   852
Northern white-cheeked gibbon       LVRSQPGRYNRARGLTFAMLAYFITWVSFVPLLANVQVVLRPAVHMGALLLCVLGILAAFHLPRCYLLMQQPGLNTPEFFLGGGPGDAQGRN--DGDTGNQGKHE   852
Northern buffed-cheeked gibbon      LVQSQPDRYNRARGLTFAMLAYFITWVSFVPLLANVQVVLRPAVHMGALLLCVLGILAAFHLPRCYLLMQQPGLNTPEFFLGGGPGDAQGRN--NGDTGNQGKHE   852
Western hoolock gibbon              LVQSQPGRYNRARGLTFAMLAYFITWVSFVPLLANVQVVLRPAVHMGALLLCVLGILAAFHLPRCYLLMQQPGLNTPKFFLGGGPGDAQGRN--DGDTGNQGKHE   852
Hylobates muelleri abbotti          LVQSQPGRYNRARGLTFAMLAYFITWVSFVPLLANVQVVFRPAVHMGALLLCVLGILVAFHLPRCYLLMQQPGLNTPEFFLGGGPGDAQGRN--DGDTGNQGKHE   852
Agile gibbon                        LVQSQPGRYNRARGLTFAMLAYFITWVSFVPLLANVQVVFRPAVHMGALLLCVLGILAAFHLPRCYQLMQQPGLNTPEFFLGGGPGDAQGRN--DGDTGNQGKHE   852
Common gibbon                       LVQSQPGRYNRARGLTFAMLAYFITWVSFVPLLANVQVVLRPAVHMGALLLCVLGILAAFHLPRCYLLMQQPGLNTPEFFLGGGPGDAQGRN--DGDTGNQGKHE   852
Pileated gibbon                     LVQSQPGRYNRARGLTFAMLAYFITWVSFVPLLANVQVVLRPAVHMGALLLCVLGILAAFHLPRCYLLMQQPGLNTPEFFLGGGPGDAQGRN--DGDTGNQGKHE   852
Siamang                             LVQSQPGRYNRARGLTFAMLAYFITWVSFVPLLANVQVVLRPAVHMGALLLCVLGILAAFHLPRCYLLMQQPGLNTPEFFLGGGPGDAQGRN--DGDTGNQGKHE   852
Green monkey                        LVQSRPGRYNRARGLTFAMLAYFITWVSFVPLLANVQVVLRPAVQMGALLLCVLGILAAFHLPRCYLLVRQPELNTPEFFLGRGPGDARDRN--DGDTGNQGKHE   852
Golden-bellied mangabey             LVQSRPGRYNRARGLTFAMLAYFITWVSFVPLLANVQVVFRPAVQMGALLLCVLGILAAFHLPRCYLLVRQPELNTPEFFLGRGPGDARDRN--DGHTGNQGKHE   852
Sooty mangabey                      LVQSRPGRYNRARGLTFAMLAYFITWVSFVPLLANVQVVFRPAVQMGALLLCVLGILAAFHLPRCYLLVRQPELNTPEFFLGRGPGDARDRN--DGHTGNQGKHE   852
Blue monkey                         LVQSRPGRYNRARGLTFAMLAYFITWVSFVPLLANVQVVLRPAVQMGALLLCVLGILAAFHLPRCYLLVRQPELNTPEFFLGRGPGDARDRN--DGDTGNQGKHE   852
Sykes' monkey                       LVQSRPGRYNRARGLTFAMLAYFITWVSFVPLLANVQVVLRPAVQMGALLLCVLGILAAFHLPRCYLLVRQPELNTPEFFLGRGPGDARDRN--DGDTGNQGKHE   852
Assam macaque                       LVQSRPGRYNRARGLTFAMLAYFITWVSFVPLLANVQVVLRPAVQMGALLLCVLGILAAFHLPRCYLLVQQPELNTPEFFLGRGPGDARDRN--DGDTGNQGKHE   852
Stump-tailed macaque                LVQSRPGRYNRARGLTFAMLAYFITWVSFVPLLANVQVVLRPAVQMGALLLCVLGILAAFHLPRCYLLVRQPELNTPEFFLGRGPGDARDRN--DGDTGNQGKHE   852
Japanese macaque                    LVQSRPGRYNRARGLTFAMLAYFITWVSFVPLLANVQVVLRPAVQMGALLLCVLGILAAFHLPRCYLLVRQPELNTPEFFLGRGPGDARDRN--DGDTGNQGKHE   852
Crab-eating macaque                 LVQSRPGRYNRARGLTFAMLAYFITWVSFVPLLANVQVVLRPAVQMGALLLCVLGILAAFHLPRCYLLVRQPELNTPEFFLGRGPGDARDRN--DGDTGNQGKHE   852
Pig-tailed macaque                  LVQSRPGRYNRARGLTFAMLAYFITWVSFVPLLANVQVVLRPAVQMGALLLCVLGILAAFHLPRCYLLVRQPELNTPEFFLGRGPGDARDRN--DGDTGNQGKHE   852
Rhesus macaque                      LVQSRPGRYNRARGLTFAMLAYFITWVSFVPLLANVQVVLRPAVQMGALLLCVLGILAAFHLPRCYLLVRQPELNTPEFFLGRGPGDARDRN--DGDTGNQGKHE   852
Gelada baboon                       LVQSRPGRYNRARGLTFAMLAYFITWVSFVPLLANVQVVLRPAVQMGALLLCVLGILAAFHLPRCYLLVRQPELNTPEFFLGRGPGDARDRN--DGDTGNQGKHE   852
Hamadryas baboon                    LVQSRPGRYNRARGLTFAMLAYFITWVSFVPLLANVQVVLRPAVQMGALLLCVLGILAAFHLPRCYLLVRQPELNTPEFFLGRGPGDARDRN--DGDTGNQGKHE   852
Mandrill                            LVQSRPGRYNRARGLTFAMLAYFITWVSFVPLLANVQVVFRPAVQMGALLLCVLGILAAFHLPRCYLLVRQPELNTPEFFLGRGPGDARDRN--DGHTGNQGKHE   852
Drill                               LVQSRPGRYNRARGLTFAMLAYFITWVSFVPLLANAQVVFRPAVQMGALLLCVLGILAAFHLPRCYLLVRQPELNTPEFFLGRGPGDARDRN--DGHTGNQGKHE   852
Red guenon                          LVQSRPGRYNRARGLTFAMLAYFITWVSFVPLLANVQVVLRPAVQMGALLLCVLGILAAFHLPRCYLLVRQPELNTPEFFLGRGPGDARDRN--DGDTGNQGNHE   852
Ma's night monkey                   LVQSQPGRYNRARGLTFATLAYFITWVSFVPLLANVQVALRPAMQMGAFLLCTLGILAAFHLPRCYLLLWQPGLNTPEFFLGGARMPKAGMA--VGTEEAQGKNE   852
White-tufted-ear marmoset           LVQSQPGRYNRARGLTFAMLAYFITWVSFVPLLANVQVALRPAMQMGAFLLCTLGILAAFHLPRCYLLLWQPGLNTPEFFLGGARMPNAGMA--VGTEEAQGKIE   852
Panamanian white-faced capuchin     LVQSQPGRYNRARGLTFAMLAYFIIWVSFVPLLANVQVALRPAMQMGAFLLCTLGILTAFHLPRCYLLLWQPGLNTPEFFLGGAQMPKVGMV--VETEEAQGKK-   851
Brown-capped capuchin               LVQSQPGRYNRARGLTFAMLAYFIIWVSFVPLLANVQVALRPAMQMGAFLLCTLGILTAFHLPRCYLLLWQPGLNTPEFFLGGAQMPKVGMV--VETEEAQGKK-   851
Bolivian squirrel monkey            LVQSQPGRYNRARGLTFAMLAYFITWVSFVPLLANVEVALRPAVQMGAFLLCTLGILAAFHLPRCYLLLWQPGLNTPEFFLGGAQIPKVGMV--VGTEEAQGKNE   852
Common squirrel monkey              LVQSQPGRYNRARGLTFAMLAYFITWVSFVPLLANVEVALRPAVQMGAFLLCTLGILAAFHLPRCYLLLWQPGLNTPEFFLGGAQIPKVGMV--VGTEEAQGKNE   852
Coquerel's sifaka                   LVQSRPRRYSHARGITFAMLAYLIIWVSFVPLLANVQVAYQPAVQMGALLLCALGILAACHLPKCYLLLWQPELNTPEFFLGGGPGDATGRDSGQGEEGTRSKNK   854
Gray mouse lemur                    LVQSRPSRYSRARGITFATLAYLITWVSFVPLLANVQVAYQPAVQMGALLLCALGILAAVHLPKCYLLLWQPELNTPEFFLGGAPGEATGRDGSRGEEGTRGKSE   855
Ring-tailed lemur                   LVQSRPSRYSRARGITFATLAYLITWVSFVPLLANVQVAYQPAVQMGALLLSALGILAAFHLPKCYLLLWQPELNTPEFFLGGGPGDATGRDGNRAEEGTRGKSE   854
```
