# Supplementary material for: Systematic analysis reveals novel insight into the molecular determinants of function, diversity and evolution of sweet taste receptors T1R2/T1R3 in primates
Source: Front Mol Biosci. 2023 Jan 25;10:1037966. doi: 10.3389/fmolb.2023.1037966 (PMC9905694; doi:10.3389/fmolb.2023.1037966)
Supplement: Supplementary file 6 [file DataSheet1.DOCX]

**Supplemental Dataset**

Protein sequences of T1R2s in primates analyzed in this study (Common name, scientific name and Genbank accession number of the species are annotated respectively in the FASTA format):

>Human (*Homo sapiens*) Q8TE23

MGPRAKTISSLFFLLWVLAEPAENSDFYLPGDYLLGGLFSLHANMKGIVHLNFLQVPMCKEYEVKVIGYNLMQAMRFAVEEINNDSSLLPGVLLGYEIVDVCYISNNVQPVLYFLAHEDNLLPIQEDYSNYISRVVAVIGPDNSESVMTVANFLSLFLLPQITYSAISDELRDKVRFPALLRTTPSADHHIEAMVQLMLHFRWNWIIVLVSSDTYGRDNGQLLGERVARRDICIAFQETLPTLQPNQNMTSEERQRLVTIVDKLQQSTARVVVVFSPDLTLYHFFNEVLRQNFTGAVWIASESWAIDPVLHNLTELRHLGTFLGITIQSVPIPGFSEFREWGPQAGPPPLSRTSQSYTCNQECDNCLNATLSFNTILRLSGERVVYSVYSAVYAVAHALHSLLGCDKSTCTKRVVYPWQLLEEIWKVNFTLLDHQIFFDPQGDVALHLEIVQWQWDRSQNPFQSVASYYPLQRQLKNIQDISWHTINNTIPMSMCSKRCQSGQKKKPVGIHVCCFECIDCLPGTFLNHTEDEYECQACPNNEWSYQSETSCFKRQLVFLEWHEAPTIAVALLAALGFLSTLAILVIFWRHFQTPIVRSAGGPMCFLMLTLLLVAYMVVPVYVGPPKVSTCLCRQALFPLCFTICISCIAVRSFQIVCAFKMASRFPRAYSYWVRYQGPYVSMAFITVLKMVIVVIGMLATGLSPTTRTDPDDPKITIVSCNPNYRNSLLFNTSLDLLLSVVGFSFAYMGKELPTNYNEAKFITLSMTFYFTSSVSLCTFMSAYSGVLVTIVDLLVTVLNLLAISLGYFGPKCYMILFYPERNTPAYFNSMIQGYTMRRD

>Western lowland gorilla (*Gorilla gorilla gorilla*) A3QNZ9

MGTRATTICSLFFLLWVLAEPAENSDFYLPGDYLLGGLFSLHANMKGIVHLNFLQVPMCKEYEVKVIGYNLMQAMRFAVEEINNDSSLLPGVLLGYEIVDVCYISNNVQPVLYFLAHEDNLLPIQEDYSNYISRVVAVIGPDNSESVMTVANFLSLFLLPQITYSAISDELQDKVRFPALLRTTPSADHHVEAMVQLMLHFRWNWIIVLVSSDTYGRDNGQLLGERLARRDICIAFQETLPTLQPNQNMTSEERQRLVTIVDKLQQSTARVVVVFSPDLSLYDFFNEVLRQNFTGAVWIASESWAIDPVLHNLTELRHLGTFLGITIQSVPIPGFSEFREWSPQAGPPPLSRTSQSYTCNQECDNCLNATLSFNTILRLSGERVVYSVYSAVYAVAHALHSLLGCDNSTCTKRVVYPWQLLEEIWKVNFTLLDHQIFFDPQGDVALHLEIVQWQWDRSQNPFQSVASYYPLQRQLKHIQDISWHTINNTIPVSMCSKRCQSGQKKKPVGIHVCCFECIDCLPGTFLNHTEDEYECQACPNNEWSYQSETSCFKRQLVFLEWHEAPTIAVALLAALGFLSTLAILVIFWRHFQTPIVRSAG

GPMCFLMLTLLLVAYMVVPVYVGPPKVSTCLCRQALFPLCFTICISCIAVRSFQIVCAFKMASRFPRAYSYWVRYQGPYVSMAFITVLKMVIVVIGMLATGLSPTTRTDPDDPKITIVSCNPNYRNSLLFNTSLDLLLSVVGFSFAYMGKELPTNYNEAKFITLSMTFYFTSSVSLCTFMSAYSGVLVTIVDLLVTVLNLLAISLGYFGPKCYMILFYPERNTPAYFNSMIQGYTMRRD

>Sumatran orangutan (*Pongo abelii*) H2N8U4

MGPRATTICSLFFLLWVLAEPAENSDFYLPGDYLLGGLFSLHANMKGIVHLNFLQVPMCKEYEVKVIGYNLMQAMRFAVEEINNDSSLLPGVLLGYEMVDVCYVSNNVQPVLYFLAHEDNLLPIQEDYSDYVSRVVAVIGPDNSESVMTVANFLSLFLLPQITYSAISDELRDKVRFPALLRTTPSADHHIEAMVQLMLHFRWNWIIVLVSSDTYGRDNGQLLGERLARRDICIAFQETLPTLQPNQNMTSEERQRLVTIVDKLQQSTARVVVVFSPDLTLYDFFNEVLRQNFTGAVWIASESWAIDPVLHNLTELRHTGTFLGITIQSVPIPGFSEFRERDSQAGPPPLGKTSQRSTCNQECDNCLNATLSFNTILRLSGERVVYSVYSAVYAVAHALHSLLGCDHSTCTKRVVYPWQLLEEIWKVNFTLLDHQIFFDPQGDMALHLEIVQWQWDRSQNPFQSVASYHPLQRQLKNIQDISWHTINNTIPVSMCSKRCQSGQKKKPVGIHVCCFECIDCLPGTFLNHTEDEYECQACPSNEWSYQSETSCFKRQLAFLEWHEAPTIAVALLAALGFLSTLAILVIFWRHFQTPMVRSAGGPMCFLMLTLLLVAYMVVPVYVGPPKVSTCLCRQALFPLCFTICISCIAVRSFQIICAFKMASRFPRAYSYWVRYQGPYVSMAFITVLKMVIVVIGMLATGLSPTTRTDPDDPKIMIVSCNPNYRNSLLFNTSLDLLLSVVGFSFAYMGKELPTNYNEAKFITLSMTFYFTSSISLCTFMSAYSGVLVTIVDLLVTVLNLLAISLGYFGPKCYMILFYPERNTPAYFNSVIQGYTMTRD

>Bornean orangutan (*Pongo pygmaeus*) A3QP00

MGPRATTICSLFFLLWVLAEPAENSDFYLPGDYLLGGLFSLHANMKGIVHLNFLQVPMCKEYEVKVIGYNLMQDMRFSVEEINNDSSLLPGVLLGYEMVDVCYVSNNVQPVLYFLAHEDNLLPIQEDYSDYVSRVVAVIGPDNSESVMTVANFLSLFLLPQITYSAISDELRDKVRFPALLRTTPSADHHIEAMVQLMLHFRWNWIIVLVSSDTYGRDNGQLLGERLARRDICIAFQETLPTLQPNQNMTSEERQRLVTIVDKLQQSTARVVVVFSPDLTLYDFFNEVLRQNFTGAVWIASESWAIDPVLHNLTELRHTGTFLGITIQSVPIPGFSEFRERDSQAGPPPLGKTSQRSTCNQECDNCLNATLSFNTILRLSGERVVYSVYSAVYAVAHALHSLLGCDHSTCTKRVVYPWQLLEEIWKVNFTLLDHQIFFDPQGDMALHLEIVQWQWDRSQNPFQSVASYHPLQRQLKNIQDISWHTINNTIPVSMCSKRCQSGQKKKPVGIHVCCFECIDCLPGTFLNHTEDEYECQACPSNEWSYQSETSCFKRQLAFLEWHEAPTIAVALLAALGFLSTLAILVIFWRHFQTPMVRSAGGPMCFLMLTLLLVAYMVVPVYVGPPKVSTCLCRQALFPLCFTICISCIAVRSFQIICAFKMASRFPRAYSYWVRYQGPYVSMAFITVLKMVIVVIGMLATGLNPTTRTDPDDPKIMIVSCNPNYRNSLLFNTSLDLLLSVVGFSFANMGKELPTNYNEAKFITLSMTFYFTSSISLCTFMSAYSGVLVTIVDLLVTVLNLLAISLGYFGPKCYMILFYPERNTPAYFNSVIQGYTMTRD

>Pygmy chimpanzee (*Pan paniscus*) A0A2R9CCZ6

MGPRAKTICSLFFLLWVLAEPAENSDFYLPGDYLLGGLFSLHANMKGIVHLNFLQVPMCKEYEVKVIGYNLMQAMRFAVEEINNDSSLLPGVLLGYEIVDVCYISNNVQPVLYFLAHEDNLLPIQEDYSNYISRVVAVIGPDNSESVMTVANFLSLFLLPQITYSAIGDELRDKVRFPALLRTTPSADHHVEAMVQLMLHFRWNWIIVLVSSDTYGRDNGQLLGERLARRDICIAFQETLPALQPNQNMTSEERQRLVTIVDKLQQSTARVVVVFSPDLSLYDFFNEVLRQNFTGAVWIASESWAIDPVLHNLTELRHLGTFLGITIQSVPIPGFSEFREWGPQAGPPPLSRTSQSYTCNQECDNCLNATLSFNTILRLSGERVVYSVYSAVYAVAHALHSLLGCDNSTCTKRVVYPWQLLEEIWKVNFTLLDHQIFFDPQGDVALHLEIVQWQWDRSQNPFQSVASYYPLQRQLKNIQDISWHTINNTIPVSMCSKRCQSGQKKKPVGIHVCCFECIDCLPGTFLNHTEDEYECQACPNNEWSYQSETSCFKRQLVFLEWHEAPTIAVALLAALGFLSTLAILVIFWRHFQTPIVRSAGGPMCFLMLTLLLVAYMVVPVYVGPPKVSTCLCRQALFPLCFTICISCIAVRSFQIVCAFKMASRFPRAYSYWVRYQGPYVSMTFITVLKMVIVVIGMLATGLSPTTRTDPDDPKITIVSCNPNYRNSLLFNTSLDLLLSVVGFSFAYMGKELPTNYNEAKFITLSMTFYFTSSVSLCTFMSAYSGVLVTIVDLLVTVLNLLAISLGYFGPKCYMILFYPERNTPAYFNSMIQGYTMRRD

>Chimpanzee (*Pan troglodytes*) A3QNZ8

MGPRAKTICSLFFLLWVLAEPAENSDFYLPGDYLLGGLFSLHANMKGIVHLNFLQVPMCKEYEVKVIGYNLMQAMRFAVEEINNDSSLLPGVLLGYEIVDVCYISNNVQPVLYFLAHEDNLLPIQEDYSNYISRVVAVIGPDNSESVMTVANFLSLFLLPQITYSAIGDELRDKVRFPALLRTTPSADHHVEAMVQLMLHFRWNWIIVLVSSDTYGRDNGQLLGERLARRDICIAFQETLPALQPNQNMTSEERQRLVTIVDKLQQSTARVVVVFSPDLSLYDFFNEVLRQNFTGAVWIASESWAIDPVLHNLTELRHLGTFLGITIQSVPIPGFSEFREWGPQAGPPPLSRTSQSYTCNQECDNCLNATLSFNTILRLSGERVVYSVYSAVYAVAHALHSLLGCDNSTCTKRVVYPWQLLEEIWKVNFTLLDHQIFFDPQGDVALHLEIVQWQWDRSQNPFQSVASYYPLQRQLKNIQDISWHTINNTIPVSMCSKRCQSGQKKKPVGIHVCCFECIDCLPGTFLNHTEDEYECQACPNNEWSYQSETSCFKRQLVFLEWHEAPTIAVALLAALGFLSTLAILVIFWRHFQTPIVRSAGGPMCFLMLTLLLVAYMVVPVYVGPPKVSTCLCRQALFPLCFTICISCIAVRSFQIVCAFKMASRFPRAYSYWVRYQGPYVSMAFITVLKMVIVVIGMLATGLSPTTRTDPDDPKITIVSCNPNYRNSLLFNTSLDLLLSVVGFSFAYMGKELPTNYNEAKFITLSMTFYFTSSVSLCTFMSAYSGVLVTIVDLLVTVLNLLAISLGYFGPKCYMILFYPERNTSAYFNSMIQGYTMRRD

>Northern white-cheeked gibbon (*Nomascus leucogenys*) G1S0K8

MGPRATTICSLFFLLRILSEPAENSDFYLPGDYLLGGLFSLHANMKGIVHLNFLQVPMCKEYEVKVIGYNLMQAMRFAVEEINNDSSLLPGVLLGYEIVDVCYISNNVQPVLYFLAHEDNLLPIQEDYSNYSSRVVAVIGPDNSESVITVANFLSLFLLPQITYSAISDELRDKVRFPALLRTTPSADHHIEAMVQLMLHFRWNWIIVLVSSDTYGRDNGQLLGERLARRDICIAFQETLPTLQPNQNMTSDERQRLVTIVDKLQQSTARVVVVFSPDLTLYDFFNEVLRQNFTGAVWIASESWAIDPVLHNLTELRHMGTFLGITIQSVPIPGFSEFRERGPQAGPPPLSRSSQRSTCNQECDNCLNATLSFNTVLRLSGERVVYSVYSAVYAVAHALHSLLNCDHSTCTKREVYPWQLLEEVWKVNFTLLDHQIFFDSQGDLALHLEIVQWQWDQSQNPFQSVASYYPLQRQLKNIQDISWHTINNTIPVSMCSKRCQSGQKKKPVGIHVCCFECIDCLPGTFLNHTEDEYECQACPNNEWSHQSETSCFKRQLVFLEWHEAPTIAVALLAALGFLSTLAILVVFWRHFQTPMVRSAGGPMCFLMLTLLLVAYMVVPVYMGPPKVSTCLCRQALFPLCFTICISCIAVRSFQIVCAFKMASRFPRAYSYWVRYQGPYVSMAFITVLKMVIVVIGMLATGLSPTTRTDPDDPKITIVSCNPNYRNSLLFNTSLDLLLSVVGFSFAYMGRELPTNYNEAKFITLSMTFYFTSSVSLCTFMSAYNGVLVTIVDLLVTVLNLLAISLGYFGPKCYMILFYPERNTPAYFNSMIQGYTMRRD

>Northern buffed-cheeked gibbon (*Nomascus annamensis*) A0A0M4Q0M8

MGPRATTICSLFFLLRILSEPAENSDFYLPGDYLLGGLFSLHANMKGIVHLNFLQVPMCKEYEVKVIGYNLMQAMRFAVEEINNDSSLLPGVLLGYEIVDVCYISNNVQPVLYFLAHEDNLLPIQEDYSNYSSRVVAVIGPDNSESVMTVANFLSLFLLPQITYSAISDELRDKVRFPALLRTTPSADHHIEAMVQLMLHFRWNWIIVLVSSDTYGRDNGQLLGERLARRDICIAFQETLPTLQPNQNMTSDERQRLVTIVDKLQQSTARVVVVFSPDLTLYDFFNEVLRQNFTGAVWIASESWAIDPVLHNLTELRHMGTFLGITIQSVPIPGFSEFRERGPQAGPPPLSRSSQRSTCNQECDNCLNATLSFNTVLRLSGERVVYSVYSAVYAVAHALHSLLNCDHSTCTKREVYPWQLLEEVWKVNFTLLDRQIFFDSQGDLALHLEIVQWQWDQSQNPFQSVASYYPLQRQLKNIQDISWHTINNTIPVSMCSKRCQSGQKKKPVGIHVCCFECIDCLPGTFLNHTEDEYECQACPNNEWSHQSETSCFKRQLVFLEWHEAPTIAVALLAALGFLSTLAILVVFWRHFQTPMVRSAGGPMCFLMLTLLLVAYMVVPVYMGPPKVSTCLCRQALFPLCFTICISCIAVRSFQIVCAFKMASRFPRAYSYWVRYQGPYVSMAFITVLKMVIVVIGMLATGLSPTTRTDPDDPKITIVSCNPNYRNSLLFNTSLDLLLSVVGFSFAYMGRELPTNYNEAKFITLSMTFYFTSSVSLCTFMSAYNGVLVTIVDLLVTVLNLLAISLGYFGPKCYMILFYPERNTPAYFNSMIQGYTMRRD

>Western hoolock gibbon (*Hoolock hoolock*) A0A0M3TYC6

MGPRAMTICSLFFLLRILSESAENSDFYLPGDYLLGGLFSLHANMKGIVHLNFLQVPMCKEYEVKVIGYNLMQAMRFAVEEINNDSSLLPGVLLGYEIVDVCYISNNVQPVLYFLAHEDNLLPIQEDYSNYSSRVVAVIGPDNSESVMTVANFLSLFLLPQITYSAISDELRDKARFPALLRTTPSADHHIEAMVQLMLHFHWNWIIVLVSSDTYGRDNGQLLGERLARRDICIAFQETLPTLQPNQNMTSDERQRLVTIVDKLQQSTARVVVVFSPDLTLYDFFNEVLRQNFTGAVWIASESWAIDPVLHNLTELRHMGTFLGITIQSVPIPGFSEFRERGPQAGPPPLSRSSQRSTCNQECDNCLNATLSFNTVLRLSGERVVYSVYSAVYAVAHALHSLLGCDHSTCTKREVYPWQLLEEVWKVNFTLLDHQIFFDSQGDLALHLEIVQWQWDKSQNPFQSVASYYPLQRQLKNIQDISWHTINNTIPVSMCSKRCQSGQKKKPVGIHVCCFECIDCLPGTFLNHTEDEYECQACPSNEWSHQSETSCFKRQLVFLEWHEAPTIAVALLAALGFLSTLAILVVFWRHFQTPMVRSAGGPMCFLMLTLLLVAYMVVPVYMGPPKVSTCLCRQALFPLCFTICISCIAVRSFQIVCAFKMASRFPRAYSYWVRYQGPYVSMAFITVLKMVIVVIGMLATGLSPTTRTDPDDPKITIVSCNPNYRNSLLFNTSLDLLLSVVGFSFAYMGRELPTNYNEAKFITLSMTFYFTSSVSLCTFMSAYNGVLVTIVDLLVTVLNLLAISLGYFGPKCYMILFYPERNTPAYFNSMIQGYTMRRD

>Hylobates muelleri abbotti A0A0M4NXV1

MGPRATTICSLFFLLRILSESAENSDFYLPGDYLLGGLFSLHANMKGIVHLNFLQVPMCKEYEVKVIGYNLMQAMRFTVEEINNDSNLLPGVLLGYEIVDVCYISNNVQPVLYFLAHEDNLLPIQEDYSNYSSRVVAVIGPDNSESVMTVANFLSLFLLPQITYSAIGDELRDKARFPALLRTTPSADHHIEAMVQLMLHFRWNWIIVLVSSDTYGRDNGQLLGERLARRDICIAFQETLPTLQPNQNMTSDERQRLVTIVDKLQQSTARVVVVFSPDLTLYDFFNEVLRQNFTGAVWIASESWAIDPVLHNLTELRHMGTFLGITIQSVPIPGFSEFRERGQQAGPPPLSRTSQRSTCNQECDDCLNATLSFNTVLRLSGERVVYSVYSAVYAVAHALHSLLDCDHSTCTKREVYPWQLLEEVWKVNFTLLDHQIFFDSQGDLALHLEIVQWQWDQSQNPFQSVASYYPLQRQLKNIQDISWHTINNTIPVSMCSKRCQSGQKKKPVGIHVCCFECIDCLPGTFLNHTEDEYECQACPNNEWSHQSETSCFKRQLVFLEWHEAPTIAVALLAALGFLSTLAILVVFWRHFQTPMVRSAGGPMCFLMLTLLLVAYMVVPVYMGPPKVSTCLCRQALFPLCFTICISCITVRSFQIVCAFKMASRFPRAYSYWVRYQGPYVSMAFITVLKMVIVVIGMLATGLSPTTRTDPDDPKITIVSCNPNYRNSLLFNTSLDLLLSVVGFSFAYMGRELPTNYNEAKFITLSMTFYFTSSVSLCTFMSAYNGVLVTIVDLLVTVLNLLAISLGYFGPKCYMILFYPERNTPAYFNSMIQGYTMRRD

>Agile gibbon (*Hylobates agilis*) A0A0M4P9G0

MGPRATTICSLFFLLRILSESAENSDFYLPGDYLLGGLFSLHANMKGIVHLNFLQVPMCKEYEVKVIGYNLMQAMRFTVEEINNDSNLLPGVLLGYEIVDVCYISNNVQPVLYFLAHEDNLLPIQEDYSNYSSRVVAVIGPDNSESVMTVANFLSLFLLPQITYSAIGDELRDKARFPALLRTTPSADHHIEAMVQLMLHFRWNWIIVLVSSDTYGRDNGQLLGERLARRDICIAFQETLPTLQPNQNMTSDERQRLVTIVDKLQQSTARVVVVFSPDLTLYDFFNEVLRQNFTGAVWIASESWAIDPVLHNLTELRHMGTFLGITIQSVPIPGFSEFRERSQQAGPPPLSRTSQRSTCNQECDDCLNATLSFNTVLRLSGERVVYSVYSAVYAVAHALHSLLDCDHSTCTKREVYPWQLLEEVWKVNFTLLDHQIFFDSQGDLALHLEIVQWQWDQSQNPFQSVASYYPLQRQLKNIQDISWHTINNTIPVSMCSKRCQSGQKKKPVGIHVCCFECIDCLPGTFLNHTEDEYECQACPNNEWSHQSETSCFKRQLVFLEWHEAPTIAVALLAALGFLSTLAILVVFWRHFQTPMVRSAGGPMCFLMLTLLLVAYMVVPVYMGPPKVSTCLCRQALFPLCFTICISCITVRSFQIVCAFKMASRFPRAYSYWVRYQGPYVSMAFITVLKMVIVVIGMLATGLSPTTRTDPDDPKITIVSCNPNYRNSLLFNTSLDLLLSVVGFSFAYMGRELPTNYNEAKFITLSMTFYFTSSVSLCTFMSAYNGVLVTIVDLLVTVLNLLAISLGYFGPKCYMILFYPERNTPAYFNSMIQGYTMRRD

>Gibbon (*Hylobates lar*) A0A0M4PYL3

MGPRATTICSLFFLLRILSESAENSDFYLPGDYLLGGLFSLHANMKGIVHLNFLQVPMCKEYEVKVIGYNLMQAMRFAVEEINNDSNLLPGVLLGYEIVDVCYISNNVQPVLYFLAHEDNLLPIQEDYSNYSSRVVAVIGPDNSESVTTVANFLSLFLLPQITYSAISDELRDKARFPALLRTTPSADHHIEAMVQLMLHFRWNWIIVLVSSDTYGRDNGQLLGERLARRDICIAFQETLPTLQPNQNMTSDERQRLVTIVDKLQQSTARVVVVFSPDLTLYDFFNEVLRQNFTGAVWIASESWAIDPVLHNLTELRHMGTFLGITIQSVPIPGFSEFRERGQQAGPPPLSRTSQRSTCNQECDDCLNATLSFNTVLRLSGERVVYSVYSAVYAVAHALHSLLDCDHSTCTKREVYPWQLLEEVWKVNFTLLDHQIFFDSQGDLALHLEIVQWQWDQSQNPFQSVASYYPLQRQLKNIQDISWHTINNTIPVSMCSKRCQSGQKKKPVGIHVCCFECIDCLPGTFLNHTEDEYECQACPNNEWSHQSETSCFKRQLVFLEWHEAPTIAVALLAALGFLSTLAILVVFWRHFQTPMVRSAGGPMCFLMLTLLLVAYMVVPVYMGPPKVSTCLCRQALFPLCFTICISCITVRSFQIVCAFKMASRFPRAYSYWVRYQGPYVSMAFITVLKMVIVVIGMLATGLSPTTRTDPDDPKITIVSCNPNYRNSLLFNTSLDLLLSVVGFSFAYMGRELPTNYNEAKFITLSMTFYFTSSVSLCTFMSAYNGVLVTIVDLLVTVLNLLAISLGYFGPKCYMILFYPERNTPAYFNSMIQGYTMRRD

>Pileated gibbon (*Hylobates pileatus*) A0A0M5LHY5

MGPRATTICSLFFLLRILSESAENSDFYLPGDYLLGGLFSLHANMKGIVHLNFLQVPTCKEYEVKVIGYNLMQAMRFAVEEINNDSNLLPGVLLGYEIVDVCYISNNVQPVLYFLAHEDNLLPIQEDYSNYSSRVVAVIGPDNSESVTTVANFLSLFLLPQITYSAISDELRDKARFPALLRTTPSADHHIEAMVQLMLHFRWNWIIVLVSSDTYGRDNGQLLGERLARRDICIAFQETLPTLQPNQNMTSDERQRLVTIVDKLQQSTARVVVVFSPDLTLYDFFNEVLRQNFTGAVWIASESWAIDPVLHNLTELRHMGTFLGITIQSVPIPGFSEFRERGQQAGPPPLSRTSQRSTCNQECDDCLNATLSFNTVLRLSGERVVYSVYSAVYAVAHALHSLLDCDHSTCTKREVYPWQLLEEVWKVNFTLLDHQIFFDSQGDLALHLEFVQWQWDQSQNPFQSVASYYPLQRQLKNIQDISWHTINNTIPVSMCSKRCQSGQKKKPVGIHVCCFECIDCLPGTFLNHTEDEYECQACPNNEWSHQSETSCFKRQLVFLEWHEAPTIAVALLAALGFLSTLAILVVFWRHFQTPMVRSAGGPMCFLMLTLLLVAYMVVPVYMGPPKVSTCLCRQALFPLCFTICISCITVRSFQIVCAFKMASRFPRAYSYWVRYQGPYVSMAFITVLKMVIVVIGMLATGLSPTTRTDPDDPKITIVSCNPNYRNSLLFNTSLDLLLSVVGFSFAYMGRELPTNYNEAKFITLSMTFYFTSSVSLCTFMSAYNGVLVTIVDLLVTVLNLLAISLGYFGPKCYMILFYPERNTPAYFNSMIQGYTMRRD

>Siamang (*Symphalangus syndactylus*) A0A0M5LP70

MGPRAMTICSLFFLLRILSEPAENSDFYLPGDYLLGGLFSLHANMKGIVHLNFLQVPMCKEYEVKVIGYNLMQAMRFAVEEINNDSSLLPGVLLGYEIVDVCYISNNVQPVLYFLAHEDNLLPIQEDYSNYSSRVVAVIGPDNSESVLTVANFLSLFLLPQITYSAISDELRDKARFPALLRTTPSADHHIEAMVQLMLHFRWNWIIVLVSSDTYGRDNGQLLGERLARRDICIAFQETLPTLQPNQNMTSDERQRLVTIVDKLQQSTARVVVVFSPDLTLYDFFNEVLRQNFTGAVWIASESWAIDPVLHNLTELRHMGTFLGITIQSVPIQGFSEFRERGPQAGPPPLSRSSQRSTCNQECDNCLNATLSFNTVLRLSGERVVYSVYSAVYAVAHALHSLLGCDHSTCTKREVYPWQLLEEVWKVNFTLLDHQIFFDSQGDLALHLEIVQWQWDQSQNPFQSVASYYPLQRQLKNIQDISWHTINNTIPVSMCSKRCQSGQKKKPVGIHVCCFECIDCLPGTFLNHTEDEYECQACPSNEWSHQSETSCFKRQLVFLEWHEAPTIAVALLAALGFLSTLAILVVFWRHFQTPMVRSAGGPMCFLMLTLLLVAYMVVPVYMGPPKVSTCLCRQALYPLCFTICISCIAVRSFQIVCAFKMASRFPRAYSYWVRYQGPYVSMAFITVLKMVIVVIGMLATGLSPTTRTDPDDPKITIVSCNPNYRNSLLFNTSLDLLLSVVGFSFAYMGRELPTNYNEAKFITLSMTFYFTSSVSLCTFMSAYNGVLVTIVDLLVTVLNLLAISLGYFGPKCYMILFYPERNTPAYFNSMIQGYTMRRD

>Green monkey (*Chlorocebus sabaeus*) A0A0D9S8E7

MRPRATTICSLFFLLQVLAEPAKNSDFYLPGDYLLGGLFTLHANMKGIVHLDYLQVPMCKEYETKVIGYNLMQAMRFAVEEINNDSSLLPDVLLGYEMVDVCYVSNNVQPVLYFLAQEDDLLPIQENYSNYVPRVVAVIGPDNSDAVMTVANFLSLFLLPQITYSAISDELRDKVRFPALLRTAPSADHHIEAIVQLMLHFRWNWIIVLVSGDTYGRDNGQLLGDRLARGDICIAFQETLPTVQPNQNMTSEERQRLVTIVDKLQQSTARVVVVFSPDLTLYNFFNEVLRQNFTGAVWIASESWAIDPVLHNLTELRHMGTFLGITIQSVPIPGFSEFRVRDPQAGPPPLSRTSQRSTCNQECDSCLNGTLSFDNVLRLSGERVVYSVYSAVYAVAHALHSLLGCDHGTCTKKEVYPWQLLKEIWKVNFTLLDHQISFDPQGDMALHLEIVQWQWGLSQNPFQSVASYYPLQRQLKKIQDISWHTINNTIPVSMCSKRCQSGQKKKPVGIHICCFECIDCLPGTFLNQTEDEYECQACPSNEWSHQSEASCFKRRLAFLEWHEAPTIVVALLAALGFLSTLAILVIFWRHFQTPMVRSAGGPMCFLMLTLLLVAYMVVPVYVGPPKVSTCFCRQALFPLCFTICISCIAVRSFQIVCVFKMASRFPRAYSYWVRYQGPYVSMAFITVLKMVTVVIGMLATGLNPTTRIDPDDPKIMIVSCNPNYRNSLFFNTSLDLLLSVVGFSFAYMGKELPTNYNEAKFITLSMTFYFTSSVSLCTFMSASNGVLVTIMDLLVTVLNLLAISLGYFGPKCYMILFYPERNTPAYFNSMIQGYTMRRD

>Golden-bellied mangabey (*Cercocebus chrysogaster*) A0A0M5LHP3

MRPRATTICSLFFLLQVLAEPAKNSDFYLPGDYLLGGLFTLHANMKGIVHLDYLQVPMCKEYETKVIGYNLMQAMRFAVEEINNDSSLLPDVLLGYEMVDVCYVSNNVQPVLYFLAQEDDLLPIQENYSNYVPRVVAVIGPDNSDAVMTVANFLSLFLLPQITYSAISDELRDKVRFPALLRTAPSADHHIEAMVQLMLHFHWNWIIVLVSGDTYGRDNGQLLGDRLARGDICIAFQETLPTVQPNQNMTSEERQRLVTIVDKLQQSTARVVVVFSPDLTLYNFFNEVLRQNFTGAVWIASESWAIDPVLHNLTELRHMGTFLGITIQSVPIPGFSEFRVRDPQAGPPPLSRTSQRSTCNQECDSCLNGTLSFNNVLRLSGERVVYSVYAAVYAVAHALHSLLGCDHGTCTKTEVYPWQLLKEIWKVNFTLLDHEISFDPQGDMALHLEIVQWQWDLSQNPFQSVASYYPLQRQLKTIQDISWHTINNTIPVSMCSKRCQSGQKKKPVGIHICCFECIDCLPGTFLNQTEDEYECQACPSNEWSHQSEASCFKRRLAFLEWHEAPTIVVALLAALGFLSTLAILVIFWRHFQTPMVRSAGGPMCFLMLTLLLVAYMVVPVYVGPPKVSTCFCRQALFPLCFTICISCIAVRSFQIVCVFKMASRFPRAYSYWVRYQGPYVSMAFITVLKMVTVVIGMLATGLNPTTRIDPDDPKIMIVSCNPNYRNSLFFNTSLDLLLSVVGFSFAYMGKELPTNYNEAKFITLSMTFYFTSSVSLCTFMSAYNGVLVTIMDLLVTVLNLLAISLGYFGPKCYMILFYPERNTPAYFNSMIQGYTMRRD

>Sooty mangabey (*Cercocebus atys*) A0A2K5MW79

MRPRATTICSLFFLLQVLAEPAKISDFYLPGDYLLGGLFTLHANMKGIVHLDYLQVPMCKEYETKVIGYNLMQAMRFAVEEINNDSSLLPDVLLGYEMVDVCYVSNNVQPVLYFLAQEDDLLPIQENYSNYVPRVVAVIGPDNSDAVMTVANFLSLFLLPQITYSAISDELRDKVRFPALLRTAPSADHHIEAMVQLMLHFHWNWIIVLVSGDTYGRDNGQLLGDRLARGDICIAFQETLPTVQPNQNMTSEERQRLVTIVDKLQQSTARVVVVFSPDLTLYNFFNEVLRQNFTGAVWIASESWAIDPVLHNLTELRHMGTFLGITIQSVPIPGFSEFRVRDPQAGPPPLSRTSQRSTCNQECDSCLNGTLSFNNVLRLSGERVVYSVYAAVYAVAHALHSLLGCDHGTCTKKEVYPWQLLKEIWKVNFTLLDHEVSFDPQGDMALHLEIVQWQWDLSQNPFQSVASYYPLQRQLKTIQDISWHTINNTIPVSMCSKRCQSGQKKKPVGIHICCFECIDCLPGTFLNQTEDEYECQACPSNEWSHQSEASCFKRRLAFLEWHEAPTIVVALLAALGFLSTLAILVIFWRHFQTPMVRSAGGPMCFLMLTLLLVAYMVVPVYVGPPKVSTCFCRQALFPLCFTICISCIAVRSFQIVCVFKMASRFPRAYSYWVRYQGPYVSMAFITVLKMVTVVIGMLATGLNPTTRIDPDDPKIMIVSCNPNYRNSLFFNTSLDLLLSVVGFSFAYMGKELPTNYNEAKFITLSMTFYFTSSVSLCTFMSAYNGVLVTIMDLLVTVLNLLAISLGYFGPKCYMILFYPERNTPAYFNSMIQGYTMRRD

>Blue monkey (*Cercopithecus mitis*) A0A0M3TY77

MRPRATTICSLFFLLQVLAEPAKNSDFYLPGDYLLGGLFTLHANVKGIVHLDYLQVPMCKEYETKVIGYNLMQAMRFAVEEINNDSSLLPDVRLGYEMVDVCYISNNVQPVLYFLAQEDDLLPIQENYSNYVPRVVAVIGPDNSDAVMTVANFLSLFLLPQITYSAISDELRDKVRFPALLRTAPSADHHIEAMVQLMLYFHWNWIIVLVSGDTYGRDNGQLLGDRLARGDICIAFQETLPTVQPNQNMTSEERQRLVTIVDKLQQSTARVVVVFSPDLTLYNFFNEVLRQNFTGAVWIASESWAIDPVLHNLTELRHMGTFLGITIQSVPIPGFSEFRVRDPQAGPPPLSRTSQRSTCNQECDSCLNGTLSFDNVLRLSGERVVYSVYSAVYAVAHALHSLLGCDHGACIKKEVYPWKLLKEIWKVNFTLLDHQISFDPQGDMALHLEIVQWQWGLSQNPFQSVASYYPLQRQLKIIQDISWHTINNTIPVSMCSKRCQSGQKKKPVGIHICCFECIDCLPGTFLNQTEDEYECQACPSNEWSHQSEASCFKRRLAFLEWHEAPTIVVALLAALGFLSTLAILVIFWRHFQTPMVRSAGGPMCFLMLTLLLVAYMVVPVYVGPPKVSTCFCRQALFPLCFTICISCIAVRSFQIVCVFKMASRFPRAYSYWVRYQGPYVSMAFITVLKMVTVVIGMLATGLNPTTRIDPDDPKIMIVSCNPNYRNSLFFNTSLDLLLSVVGFSFAYMGKELPTNYNEAKFITLSMTFYFTSSVSLCTFMSAYNGVLVTIMDLLVTVLNLLAISLGYFGPKCYMILFYPERNTPAYFNSMIQGYTMRRD

>Sykes' monkey (*Cercopithecus albogularis*) A0A0M4PM65

MRPRATTICSLFFLLQVLAEPAKNSDFYLPGDYLLGGLFTLHANVKGIVHLDYLQVPMCKEYETKVIGYNLMQAMRFAVEEINNDSSLLPDVRLGYEMVDVCYISNNVQPVLYFLAQEDDLLPIQENYSNYVPRVVAVIGPDNSDAVMTVANFLSLFLLPQITYSAISDELRDKVRFPALLRTAPSADHHIEAMVQLMLYFHWNWIIVLVSGDTYGRDNGQLLGDRLARGDICIAFQETLPTVQPNQNMTSEERQRLVTIVDKLQQSTARVVVVFSPDLTLYNFFNEVLRQNFTGAVWIASESWAIDPVLHNLTELRHMGTFLGITIQSVPIPGFSEFRVRDPQAGPPPLSRTSQRSTCNQECDSCLNGTLSFDNVLRLSGERVVYSVYSAVYAVAHALHSLLGCDHGACIKKEVYPWKLLKEIWKVNFTLLDHQISFDPQGDMALHLEIVQWQWGLSQNPFQSVASYYPLQRQLKIIQDISWHTINNTIPVSMCSKRCQSGQKKKPVGIHICCFECIDCLPGTFLNQTEDEYECQACPSNEWSHQSEASCFKRRLAFLEWHEAPTIVVALLAALGFLSTLAILVIFWRHFQTPMVRSAGGPMCFLMLTLLLVAYMVVPVYVGPPKVSTCFCRQALFPLCFTICISCIAVRSFQIVCVFKMASRFPRAYSYWVRYQGPYVSMAFITVLKMVTVVIGMLATGLNPTTRIDPDDPKIMIVSCNPNYRNSLFFNTSLDLLLSVVGFSFAYMGKELPTNYNEAKFITLSMTFYFTSSVSLCTFMSAYNGVLVTIMDLLVTVLNLLAISLGYFGPKCYMILFYPERNTPAYFNSMIQGYTMRRD

>Assam macaque (*Macaca assamensis*) A0A0M3TYC2

MRPRATTICSLFFLLRVLAEPAKNSDFYLPGDYLLGGLFTLHANMKGIVHLDYLQVPMCKEYETKVIGYNLMQAMRFAVEEINNDSSLLPDVLLGYEMVDVCYVSNNVQPVLYFLAQEDDLLPIQENYSNYVPRVVAVIGPDNSDAVMTVANFLSLFLLPQITYSAISDELRDKVRFPALLRTAPSADHHIEAMVQLMLHFRWNWIIVLVSGDTYGRDNGQLLGDRLARGDICIAFQETLPTVQPNQNMTSEERQRLVTIVDKLQQSTARVVVVFSPDLTLYNFFNEVLRQNFTGAVWIASESWAIDPVLHNLTELRHMGTFLGITIQSVPIPGFSEFRVRDPQAGPPPLSRTSQRSTCNQECDSCLNGTLSFNNVLRLSGERVVYSVYSAVYAVAHALHSLLGCDHGTCTKREVYPWQLLKEIWKVNFTLLDHEISFDPQGDMALHLEIVQWQWGLSQNPFQSVASYYPLQRQLKKIQDISWHTINNTIPVSMCSKRCQSGQKKKPVGIHICCFECIDCLPGTFLNQTEDEYECQACPSNEWSHQSEASCFKRRLAFLEWHEAPTIVVALLAALGFLSTLAILVIFWRHFQTPMVRSAGGPMCFLMLTLLLVAYMVVPVYVGPPKVSTCFCRQALFPLCFTICISCIAVRSFQIVCVFKMASRFPRAYSYWVRYQGPYVSMAFITVLKMVTVVIGMLVTGLNPTTRIDPDDPKIMIVSCNPNYRNSLFFNTGLDLLLSVVGFSFAYMGKELPTNYNEAKFITLSMTFYFTSSVSLCTFMSAYNGVLVTIMDLLVTVLNLLAISLGYFGPKCYMILFYPERNTPAYFNSMIQGYTMRRD

>Stump-tailed macaque (*Macaca arctoides*) A0A0M4NXT5

MRPRATTICSLFFLLRVLAEPAKNSDFYLPGDYLLGGLFTLHANMKGIVHLDYLQVPMCKEYETKVIGYNLMQAMRFAVEEINNDSSLLPDVLLGYEMVDVCYVSNNVQPVLYFLAQEDDLLPIQENYSNYVPRVVAVIGPDNSDAVMTVANFLSLFLLPQITYSAISDELRDKVRFPALLRTAPSADHHIEAMVQLMLHFRWNWIIVLVSGDTYGRDNGQLLGDRLARGDICIAFQETLPTVQPNQNMTSEERQRLVTIVDKLQQSTARVVVVFSPDLTLYNFFNEVLRQNFTGAVWIASESWAIDPVLHNLTELRHMGTFLGITIQSVPIPGFSEFRVRDPQAGPPPLSRTSQRSTCNQECDSCLNGTLSFNNVLRLSGERVVYSVYSAVYAVAHALHSLLGCDHGTCTKREVYPWQLLKEIWKVNFTLLDHEISFDPQGDMALHLEIVQWQWGLSQNPFQSVASYYPLQRQLKKIQDISWHTINNTIPVSMCSKRCQSGQKKKPVGIHICCFECIDCLPGTFLNQTEDEFECQACPSNEWSHQSEASCFKRRLAFLEWHEAPTIVVALLAALGFLSTLAILVIFWRHFQTPMVRSAGGPMCFLMLTLLLVAYMVVPVYVGPPKVSTCFCRQALFPLCFTICISCIAVRSFQIVCVFKMASRFPRAYSYWVRYQGPYVSMAFITVLKMVTVVIGMLATGLNPTTRIDPDDPKIMIVSCNPNYRNSLFFNTGLDLLLSVVGFSFAYMGKELPTNYNEAKFITLSMTFYFTSSVSLCTFMSAYNGVLVTIMDLLVTVLNLLAISLGYFGPKCYMILFYPERNTPAYFNSMIQGYTMRRD

>Japanese macaque (*Macaca fuscata*) A0A0M4P9E3

MRPRATTICSLFFLLWVLAEPAKNSDFYLPGDYLLGGLFTLHANMKGIVHLDYLQVPMCKEYETKVIGYNLMQAMRFAVEEINNDSSLLPDVLLGYEMVDVCYVSNNVQPVLYFLAQEDDLLPIQENYSNYVPRVVAVIGPDNSDAVMTVANFLSLFLLPQITYSAISDELRDKVRFPALLRTAPSADHHIEAMVQLMLHFRWNWIIVLVSGDTYGRDNGQLLGDRLARGDICIAFQETLPTVQPNQNMTSEERQRLVTIVDKLQQSTARVVVVFSPDLTLYNFFNEVLRQNFTGAVWIASESWAIDPVLHNLTELRHMGTFLGITIQSVPIPGFSEFRVRDPQAGPPPLSRTSQRSTCNQECDSCLNGTLSFNNVLRLSGERVVYSVYSAVYAVAHALHSLLGCDHGTCTKREVYPWQLLKEIWKVNFTLLDHEISFDPQGDMALHLEIVQWQWGLSQNPFQSVASYYPLQRQLKKIQDISWHTINNTIPVSMCSKRCQSGQKKKPVGIHICCFECIDCLPGTFLNQTEDEYECQACPSNEWSHQSEASCFKRRLAFLEWHEAPTIVVALLAALGFLSTLAILVIFWRHFQTPMVRSAGGPMCFLMLTLLLVAYMVVPVYVGPPKVSTCFCRQALFPLCFTICISCIAVRSFQIVCVFKMASRFPRAYSYWVRYQGPYVSMAFITVLKMVTVVIGMLVTGLNPTTRIDPDDPKIMIVSCNPNYRNSLFFNTGLDLLLSVVGFSFAYMGKELPTNYNEAKFITLSMTFYFTSSVSLCTFMSAYNGVLVTIMDLLVTVLNLLAISLGYFGPKCYMILFYPERNTPAYFNSMIQGYTMRRD

>Crab-eating macaque (*Macaca fascicularis*) A0A2K5TR06

MRPRATTICSLFFLLRVLAEPAKNSDFYLPGDYLLGGLFTLHANMKGIVHLDYLQVPMCKEYETKVIGYNLMQAMRFAVEEINNDSSLLPDVLLGYEMVDVCYVSNNVQPVLYFLAQEDDLLPIQENYSNYVPRVVAVIGPDNSDAVMTVANFLSLFLLPQITYSAISDELRDKVRFPALLRTAPSADHHIEAMVQLMLHFRWNWIIVLVSGDTYGRDNGQLLGDRLARGDICIAFQETLPTVQPNQNMTSEERQRLVTIVDKLQQSTARVVVVFSPDLTLYNFFNEVLRQNFTGAVWIASESWAIDPVLHNLTELRHMGTFLGITIQSVPIPGFSEFRVRDPQAGPPPLSRTSQRSTCNQECDSCLNGTLSFNNVLRLSGERVVYSVYSAVYAVAHALHSLLGCDHGTCTKREVYPWQLLKEIWKVNFTLLDHEISFDPQGDMALHLEIVQWQWGLSQNPFQSVASYYPLQRQLKKIQDISWHTINNTIPVSMCSKRCQSGQKKKPVGIHICCFECIDCLPGTFLNQTEDEYECQACPSNEWSHQSEASCFKRRLAFLEWHEAPTIVVALLAALGFLSTLAILVIFWRHFQTPMVRSAGGPMCFLMLTLLLVAYMVVPVYVGPPKVSTCFCRQALFPLCFTICISCIAVRSFQIVCVFKMASRFPRAYSYWVRYQGPYVSMAFITVLKMVTVVIGMLVTGLNPTTRIDPDDPKIMIVSCNPNYRNSLFFNTGLDLLLSVVGFSFAYMGKELPTNYNEAKFITLSMTFYFTSSVSLCTFMSAYNGVLVTIMDLLVTVLNLLAISLGYFGPKCYMILFYPERNTPAYFNSMIQGYTMRRD

>Pig-tailed macaque (*Macaca nemestrina*) A0A2K6CUQ0

MRPRATTICSLFFLLRVLAEPAKNSDFYLPGDYLLGGLFTLHANMKGIVHLDYLQVPMCKEYETKVIGYNLMQAMRFAVEEINNDSSLLPDVLLGYEMVDVCYVSNNVQPVLYFLAQEDDLLPIQENYSNYVPRVVAVIGPDNSDAVMTVANFLSLFLLPQITYSAISDELRDKVRFPALLRTAPSADHHIEAMVQLMLHFRWNWIIVLVSGDTYGRDNGQLLGDRLARGDICIAFQETLPTVQPNQNMTSEERQRLVTIVDKLQQSTARVVVVFSPDLTLYNFFNEVLRQNFTGAVWIASESWAIDPVLHNLTELRHMGTFLGITIQSVPIPGFSEFRVRDPQAGPPPLSRTSQRSTCNQECDSCLNGTLSFNNVLRLSGERVVYSVYSAVYAVAHALHSFLGCDHGTCTKREVYPWQLLKEIWKVNFTLLDHEISFDPQGDMALHLEIVQWQWGLSQNPFQSVASYYPLQRQLKKIQDISWHTINNTIPVSMCSKRCQSGQKKKPVGIHICCFECIDCLPGTFLNQTEDEYECQACPSNEWSHQSEASCFKRRLAFLEWHEAPTIVVALLAALGFLSTLAILVIFWRHFQTPMVRSAGGPMCFLMLTLLLVAYMVVPVYVGPPKVSTCFCRQALFPLCFTICISCIAVRSFQIVCVFKMASRFPRAYRYWVRYQGPYVSMAFITVLKMVTVVIGMLATGLNPTTRIDPDDPKIMIVSCNPNYRNSLFFNTGLDLLLSVVGFSFAYMGKELPTNYNEAKFITLSMTFYFTSSVSLCTFMSAYNGVLVTIMDLLVTVLNLLAISLGYFGPKCYMILFYPERNTPAYFNSMIQGYTMRRD

>Rhesus macaque (*Macaca mulatta*) A3QP01

MRPRATTICSLFFLLRVLAEPAKNSDFYLPGDYLLGGLFTLHANMKGIVHLDYLQVPMCKEYETKVIGYNLMQAMRFAVEEINNDSSLLPDVLLGYEMVDVCYVSNNVQPVLYFLAQEDDLLPIQENYSNYVPRVVAVIGPDNSDAVMTVANFLSLFLLPQITYSAISDELRDKVRFPALLRTAPSADHHIEAMVQLMLHFRWNWIIVLVSGDTYGRDNGQLLGDRLARGDICIAFQETLPTVQPNQNMTSEERQRLVTIVDKLQQSTARVVVVFSPDLTLYNFFNEVLRQNFTGAVWIASESWAIDPVLHNLTELRHMGTFLGITIQSVPIPGFSEFRVRDPQAGPPPLSRTSQRSTCNQECDSCLNGTLSFNNVLRLSGERVVYSVYSAVYAVAHALHSLLGCDHGTCTKREVYPWQLLKEIWKVNFTLLDHEISFDPQGDMALHLEIVQWQWGLSQNPFQSVASYYPLQRQLKKIQDISWHTINNTIPVSMCSKRCQSGQKKKPVGIHICCFECIDCLPGTFLNQTEDEFECQACPSNEWSHQSEASCFKRRLAFLEWHEAPTIVVALLAALGFLSTLAILVIFWRHFQTPMVRSAGGPMCFLMLTLLLVAYMVVPVYVGPPKVSTCFCRQALFPLCFTICISCIAVRSFQIVCVFKMASRFPRAYSYWVRYQGPYVSMAFITVLKMVTVVIGMLATGLNPTTRIDPDDPKIMIVSCNPNYRNSLFFNTGLDLLLSVVGFSFAYMGKELPTNYNEAKFITLSMTFYFTSSVSLCTFMSAYNGVLVTIMDLLVTVLNLLAISLGYFGPKCYMILFYPERNTPAYFNSMIQGYTMRRD

>Gelada baboon (*Theropithecus gelada*) A0A0M4NTR8

MRPRETTICSLFFLLRVLAEPAKNSDFYLPGDYLLGGLFTLHANVKGIVHLDYLQVPMCKEYETKVIGYNLMQAMRFAVEEINNDSSLLPDVLLGYEMVDVCYVSNNVQPVLYFLAQEDDLLPIQENYSNYVPRVVAVIGPDNSDAVMTVANFLSLFLLPQITYSAISDELRDKVRFPALLRTAPSADHHIEAMVQLMLHFRWNWIIVLVSGDTYGRDNGQLLGDRLARGDICIAFQETLPTVQPNQNMTSEERQRLVTIVDKLQQSTARVVVVFSPDLTLYNFFNEVLRQNFTGAVWIASESWAIDPVLHNLTELRHVGTFLGITIQSVPIPGFSEFRVRDPQAGPPPLSRTSQRSTCNQECDSCLNGTLSFNNVLRLSGERVVYSVYSAVYAVAHALHSLLGCDHGTCTKTEVYPWQLLKEIWKVNFTLLDHEISFDPQGDMALHLEIVQWQWDLSQNPFQSVASYYPLQRQLKKIQDISWHTINNTIPVSMCSKRCQSGQKKKPVGIHICCFECIDCLPGTFLNQTEDEYECQACPSNEWSHQSEASCFKRRLAFLEWHEAPTIVVALLAALGFLSTLAILVIFWRHFQTPMVRSAGGPMCFLMLTLLLVAYMVVPVYVGPPKVSTCFCRQALFPLCFTICISCIAVRSFQIVCVFKMASRFPRAYSYWVRYQGPYVSMAFITVLKMVTVVIGMLATGLNPTTRIDPDDPKIMIVSCNPNYRNSLFFNTGLDLLLSVVGFSFAYMGKELPTNYNEAKFITLSMTFYFTSSVSLCTFMSAYNGVLVTIMDLLVTVLNLLAISLGYFGPKCYMILFYPERNTPAYFNSMIQGYTMRRD

>Hamadryas baboon (*Papio hamadryas*) A3QP07

MRPRATTICSLFFLLRVLAEPAKNSDFYLPGDYLLGGLFTLHANMKGIVHLDYLQVPMCKEYETKVIGYNLMQAMRFAVEEINNDSSLLPDVLLGYEMVDVCYVSNNVQPVLYFLAQEDDLLPIQENYSNYVSRVVAVIGPDNSDAVMTVANFLSLFLLPQITYSAISDELRDKVRFPALLRTAPSADHHIEAMVQLMLHFRWNWIIVLVSGDTYGRDNGQLLGDRLARGDICIAFQETLPTVQPNQNMTSEERQRLVTIVDKLQQSTARVVVVFSPDLTLYNFFNEVLRQNFTGAVWIASESWAIDPVLHNLTELRHMGTFLGITIQSVPIPGFSEFRVRDPQAGPPPLSRTSQRSTCNQECDSCLNGTLSFNNVLRLSGERVVYSVYSAVYAVAHALHSLLGCDHGTCTKTEVYPWQLLKEIWKVNFTLLDHQISFDPQGDMALHLEIVQWQWDLSQNPFQSVASYYPLQRQLKTIQDISWHTINNTIPVSMCSKRCQSGQKKKPVGIHICCFECIDCLPGTFLNQTEDEYECQACPSNEWSHQSEASCFKRRLAFLEWHEAPTIVVALLAALGFLSTLAILVIFWRHFQTPMVRSAGGPMCFLMLTLLLVAYMVVPVYVGPPKVSTCFCRQALFPLCFTICISCIAVRSFQIVCVFKMASRFPRAYSYWVRYQGPYVSMAFITVLKMVTVVIGMLATGLNPTTRIDPDDPKIMIVSCNPNYRNSLFFNTGLDLLLSVVGFSFAYMGKELPTNYNEAKFITLSMTFYFTSSVSLCTFMSAYNGVLVTIMDLLVTVLNLLAISLGYFGPKCYMILFYPERNTPAYFNSMIQGYTMRRD

>Black crested mangabey (*Lophocebus aterrimus*) A0A0M4P9T4

MRPRATTICSLFFLLRVLAEPAKNSDFYLPGDYLLGGLFTLHANMKGIVHLDYLQVPMCKEYETKVIGYNLMQAMRFAVEEINNDSSLLPDVLLGYEMVDVCYVSNNVQPVLYFLAQEDDLLPIQENYSNYVPRVVAVIGPDNSDAVMTVANFLSLFLLPQITYSAISDELRDKVRFPALLRTAPSADHHIEAMVQLMLHFRWNWIIVLVSGDTYGRDNGQLLGDRLARGDICIAFQETLPTVQPNQNMTSEERQRLVTIVDKLQQSTARVVVVFSPDLTLYDFFNEVLRQNFTGAVWIASESWAIDPVLHNLTELRHMGTFLGITIQSVPIPGFSEFRVRDPQAGPPPLSRTSQRSTCNQECDSCLNGTLSFNNVLRLSGERVVYSVYSAVYAVAHALHSLLGCDHGTCAKTEVYPWQLLKEIWKVNFTLLDHQIAFDPQGDMALHLEIVQWQWDLSQNPFQSVASYYPLQRQLKTIQDISWHTINNTIPVSMCSKRCQSGQKKKPVGIHICCFECIDCLPGTFLNQTEDEYECQACPSNEWSHQSEASCFKRRLAFLEWHEAPTIVVALLAALGFLSTLAILVIFWRHFQTPMVRSAGGPMCFLMLTLLLVAYMVVPVYVGPPKVSTCFCRQALFPLCFTICISCIAVRSFQIVCVFKMASRFPRAYSYWVRYQGPYVSMAFITVLKMVTVVIGMLATGLNPTTRIDPDDPKIMIVSCNPNYRNSLFFNTSLDLLLSVVGFSFAYMGKELPTNYNEAKFITLSMTFYFTSSVSLCTFMSAYNGVLVTIMDLLVTVLNLLAISLGYFGPKCYMILFYPERNTPAYFNSMIQGYTMRRD

>Mandrill (*Mandrillus sphinx*) A0A0M4Q0L2

MRPRATTICSLFFLLRVLAEPAKNSDFYLPGDYLLGGLFTLHANMKGIVHLDYLQVPMCKEYETKVIGYNLMQAMRFAVEEINNDSSLLPDVLLGYEMVDVCYVSNNVQPVLYFLAQEDDLLPIQENYSNYMPRVVAVIGPDNSDAVMTVANFLSLFLLPQITYSAISDELRDKVRFPALLRTAPSADHHIEAMVQLMLHFRWNWIIVLVSGDTYGRDNGQLLGDRLARGDICIAFQETLPTVQPNQNMTSEERQRLVTIVDKLQQSTARVVVVFSPDLTLYNFFNEVLRQNFTGAVWIASESWAIDPVLHNLTELRHMGTFLGITIQSVPIPGFSEFRVRDPQAGPPPLSRTSQRSTCNQECDSCLNGTLSFNNVLRLSGERVVYSVYSAVYAVAHALHSLLGCDYGTCAKKEVYPWQLLKEIWKVNFTLLDHEISFDPQGDMALHLEIVQWQWDLSQNPFQSVASYYPLQRQLKTIQDISWHTINNTIPVSMCSKRCQSGQKKKPVGIHICCFECIDCLPGTFLNQTEDEYECQACPSNEWSHQSEASCFKRRLAFLEWHEAPTIVVALLAALGFLSTLAILVIFWRHFQTPMVRSAGGPMCFLMLTLLLVAYMVVPVYVGPPKVSTCFCRQALFPLCFTICISCIAVRSFQIVCVFKMASRFPRAYSYWVRYQGPYVSMAFITVLKMVTVVIGMLATGLNPTTRIDPDDPKIMIVSCNPNYRNSLFFNTSLDLLLSVVGFSFAYMGKELPTNYNEAKFITLSMTFYFTSSVSLCTFMSVYNGVLVTIMDLLVTVLNLLAISLGYFGPKCYMILFYPERNTPAYFNSMIQGYTMRRD

>Drill (*Mandrillus leucophaeus*) A0A2K6A7C6

MRPRATTICSLFFLLQVLAEPAKNSDFYLPGDYLLGGLFTLHANMKGIVHLDYLQVPMCKEYETKVIGYNLMQAMRFAVEEINNDSSLLPDVLLGYEMVDVCYVSNNVQPVLYFLAQEDDLLPIQENYSNYMPRVVAVIGPDNSDAVMTVANFLSLFLLPQITYSAISDELRDKVRFPALLRTAPSADHHIEAMVQLMLHFRWNWIIVLVSGDTYGRDNGQLLGDRLARGDICIAFQETLPTVQPNQNMTSEERQRLVTIVDKLQQSTARVVVVFSPDLTLYNFFNEVLRQNFTGAVWIASESWAIDPVLHNLTELRHMGTFLGITIQSVPIPGFSEFRVRDPQAGPPPLSRTSQRSTCNQECDSCLNGTLSFNNVLRLSGERVVYSVYCAVYAVAHALHSLLGCDYGTCAKKEVYPWQLLKEIWKVNFTLLDHEISFDPQGDMALHLEIVQWQWDLSQNPFQSVASYYPLQRQLKTIQDISWHTINNTIPVSMCSKRCQSGQKKKPVGIHICCFECIDCLPGTFLNQTEDEYECQACPSNEWSHQSEASCFKRRLAFLEWHEAPTIVVALLAALGFLSTLAILVIFWRHFQTPMVRSAGGPMCFLMLTLLLVAYMVVPVYVGPPKVSTCFCRQALFPLCFTICISCIAVRSFQIVCVFKMASRFPRAYSYWVRYQGPYVSMAFITVLKMVTVVIGMLATGLNPTTRIDPDDPKIMIVSCNPNYRNSLFFNTSLDLLLSVVGFSFAYMGKELPTNYNEAKFITLSMTFYFTSSVSLCTFMSVYNGVLVTIMDLLVTVLNLLAISLGYFGPKCYMILFYPKRNTPAYFNSMIQGYTMRRD

>Red guenon (*Erythrocebus patas*) A0A0M5LHS1

MRPRATTICSLFFLLQVLAEPAKNSDFYLPGDYLLGGLFTLHANMKGIVHLDYLQVPMCKEYETKVIGYNLMQAMRFAVEEINNDSSLLPDVLLGYEMVDVCYVSNNVQPVLYFLAQEDDLLPIQENYSNYVPRVVAVIGPDNSDAVMTVANFLSLFLLPQITYSAISDELRDKVRFPALLRTAPSADHHIEAMVQLMLYFHWNWIIVLVSGDTYGRDNGQLLGDRLARGDICIAFQETLPTVQPNQNMTSEERQRLVTIVDKLQQSTARVVVVFSPDLTLYNFFNEVLRQNFTGAVWIASESWAIDPVLHNLTELRHMGTFLGITIQSVPIPGFSEFRVRDPQAGPPPLSRSSQRSTCNQECDSCLNGTLSFDNVLRLSGERVVYSVYSAVYAVAHALHSLLGCDHGTCTKKEVYPWQLLKEIWKVNFTLLDHQISFDPQGDMALHLEIVQWQWGLSQNPFQSVASYYPLQRQLKKIQDISWHTINNTIPVSMCSKRCQSGQKKKPVGIHICCFECIDCLPGTFLNQTEDEYECQACPSNEWSHQSEASCFKRRLAFLEWHEAPTIVVALLAALGFLSTLAILVIFWRHFQTPMVRSAGGPMCFLMLTLLLVAYMVVPVYVGPPKVSTCFCRQALFPLCFTICISCIAVRSFQIVCVFKMASRFPRAYSYWVRYQGPYVSMAFITVLKMVTVVIGMLATGLNPTTRIDPDDPKIMIVSCNPNYRNSLFFNTSLDLLLSVVGFSFAYMGKELPTNYNEAKFITLSMTFYFTSSVSLCTFMSAYNGVLVTIMDLLVTVLNLLAISLGYFGPKCYMILFYPERNTPAYFNSMIQGYTMRRD

>Ma's night monkey (*Aotus nancymaae*) A0A2K5C879

MGSRARTVCFLFFLLWVLAEPAENSDFHLPGDYLLGGLFTLHANMKGTVHLNFLQVPMCKEYEMKVSGYNLMQAMRFAVEEINNDSSLLPDVLLGYEMVDVCYISNNVQPVLYFLAQEDSLLPIQEDYSNYVPRVVAVIGPENSESVTTVANFLSLFLLPQITYSAISDQLRDKQRFPALLRTAPSAKHHIEAMVQLMLHFHWNWISVLVSSDTYGRDNGQLLGDRLAGGDICIAFQETLPTLQPNQDITPEDRQRLVSIVEKLQQSTARVVVVFSPDLNLYDFFREVVRQNFTGAVWIASESWAIDPVLHNVTGLHRTGTFLGITIQNVPIPGFSEFRVRGPQAGPTNQRSTCNQECDTCLNSTLSFNTILRLSGERIVYSVYSAVYAVAHALHSLLGCDHSACTKRVVYPWQLLEEIWKVNFTLLDHQIFFDPQGDVALHLEIVQWQWDLSQNPFQSIASYSPLQGQLKHIQDISWHTVNNTIPVSMCSKRCQSGQKKKPVGIHTCCFECIDCLPGTFLNQTANEYDCQTCPSNEWSHQSETSCFKRRLSFLEWHEAPTIAVALLAALGFLSTLAILVIFWRHFQTPMVRSAGGPMCFLMLTLLLVAYTVVPVYVGPPKVSTCLCRQALFPVCFTICISCITVRSFQIVCVFKMASRFPRAYSYWVRYQGTYVSVAFITALKIVTVVISSLATGLNPTTRTDTDDPKIMIISCNPNYRNSLLFNTSLDLLLSVVGFSFAYMGKELPTNYNEAKFITFSMTFYFTSSVSLCTFMSVYNGVLVTIVDLLVTVFNLLAISLGYFGPKCYMILFYPERNTPAYFNSMIQGYTMRRD

>White-tufted-ear marmoset (*Callithrix jacchus*) XP_017829865

MGPRARTVCFLFFLLWVLAELAENSDFHLPGDYLLGGLFTLHANMKGIVHLNFLQVPMCKEYEMKVSGYNLMQAMRFAVEEINNDSSLLPNVLLGYEMVDVCYISNNVQPALYFLAQEDNLLPIREDYSNYVPRVVAVIGPENSESVMTVAHFLSLFLLPQITYSAISDQLQDKQRFPALLRTTPSAKHHIEAMVQLMLHFHWNWISVLVSSDTYGRDNGQMLGDRLAGGDICIAFQETLPTLQSNQDIMPEDHQRLVSIVEKLQQSTARVVVVFSPDLSLYNFFREVLRQNFTGAVWIASESWAIDPVLHNLTGLHRTGTFLGITIQNVPIPGFSEFRVRGPQAEPTNQRSTCNQECDTCLNSTLSFNTVLRLSGERIVYSVYSAVYAVAHALHSLLSCDHSTCTKRVVYPWQLLEEIWKVNFTLLDHQIFFDPQGDVALHLEIVQWQWDLSQNPFQSIASYNPLQGRLKHIQDISWHTINNTIPVSMCSKRCQSGQKKKPVGIHTCCFECIDCLPGTFLNQTANEYDCQACLSNEWSHQSETSCFKRRLSFLEWHEAPTIAVALLAALGFLSTLAILVIFWRHFHTPMVRSAGGPMCFLMLTLLLVAYMVVPVYVGPPKVTTCLCRQALFPVCFTICISCITMRSFQIVCVFKMASRFPRAYSYWVRYQGSYVSVAFITALKVVTVVISLLATGLNPTTRADTDDPKIMIISCNPNYRNSLLFNTSLDLLLSVVGFSFAYMGKELPTNYNEAKFITFSMTFYFTSSVSLCTFMSVYDGVLVTIVDLLVTVFNLLAISLGYFGPKCYMILFYPERNTPAYFNSMIQGYTMRRD

>Panamanian white-faced capuchin (*Cebus imitator*) A0A2K5SEL6

MGPRARTVCFLFFLLWVLAELAENSDFHLPGDYLLGGLFTLHANMKGTVHLNFLQVPMCKEYEMKVSGYNLMQAMRFAVEEINNDSSLLPGVLLGYEMVDVCYISNNVQPVLYFLAQEDSLLPIQEDYSNYVPRVVAVIGPENSESVMTVANFLSLFLLPQITYSAISDQLRDKQRFPALLRTAPSAKHHIEAMVQLMLHFHWNWISVLVSSDTYGRDNGQLLGDRLAGSDICIAFQETLPTLQPNQDIMPEDRQRLVSIVEKLQHSTARVVVVFSPDLTLYDFFREVLRQNFTGAVWIASESWSIDPVLHNLTGLHRTGTFLGITIQNVPIPGFSEFRVRGPQAEPTNQRSTCNQECDTCLNSTLSFNSILRLSGERIVYSVYSAVYVVAHALHSLLGCDHSACTKRVVYPWQLLEEIWKVNFTLLDHQISFDPHGDVALHLEIVQWQWDLNQNLFQSVASYSPLQGHLKDIQDISWHTVNNTIPVSMCSKRCQSGQKKKPMGIHTCCFECIDCLPGTFLNQTANEYDCQACPSNEWSHQSETSCFKRRLSFLEWHEAATIAVALLAALGFLSTLAILVIFWRHFQTPMVRSAGGPMCFLMLTLLLVAYMVVPVYVGLPKVSTCLCRQALFPVCFTICISCIAVRSFQIVCVFKMASRFPRAYSYWVRYQGSYVSVAFITALKMITVVISLLATGLNPTTRTDTDDPKIMIISCNPNYRNSLLFNTSLDLLLSVVGFSFAYMGKELPTNYNEAKFITFSMTFYFTSSVSLCTFMSVYDGVLVTIVDLLVTVFNLLAISLGYFGPKCYMILFYPERNTPAYFNSMIQGYTMRRD

>Brown-capped capuchin (*Sapajus apella*) A0A6J3JPS2

MGPRVRTVCFLFFLLWVLAEPAETSDFHLPGDYLLGGLFTLHANMKGTVHLNFLQVPMCKEYEMKVSGYNLMQAMRFAVEEINNDSSLLPDVLLGYEMVDVCYISNNVQPVLYFLAQEDSLLPIQEDYSNYVPRVVAVIGPENSESVMTVANFLSLFLLPQITYSAISDQLRDKQRFPALLRTAPSAKHHIEAMVQLMLHFHWNWISVLVSSDTYGRDNGQLLGDRLVGSDICIAFQETLPTLQPNQDIMPEDRQRLVSIVEKLQHSTARVVVVFSPDLTLYDFFREVLRQNFTGAVWIASESWSIDPVLHNLMGLHRTGTFLGITIQNVPIPGFSEFRVRGPQAEPTNQRSTCNQECDTCLNSTLSFNSILRLSGERIVYSVYSAVYAVAHALHSLLGCDHSACTKRVVYPWQLLEEIWKVNFTLLDHQISFDPQGDVALHLEIVQWQWDLNQNLFQSVASYSPLQGHLKDIQDISWHTVNNTIPVSMCSKRCQSGQKKKPMGIHTCCFECIDCLPGTFLNQTANEYDCQACPSNEWSHQSETSCFKRRLSFLEWHEAATIAVALLAALGFLSTLAILVIFWRHFQTPMVRSAGGPMCFLMLTLLLVAYMVVPVYVGLPKVSTCLCRQALFPVCFTICISCIAVRSFQIVCVFKMASRFPRAYSYWVRYQGSYVSVAFITALKMITVVISLLATGLNPTTRTDTDDPKIMIISCNPNYRNSLLFNTSLDLLLSVVGFSFAYMGKELPTNYNEAKFITFSMTFYFTSSVSLCTFMSVYDGVLVTIVDLLVTVFNLLAISLGYFGPKCYMILFYPERNTPAYFNSMIQGYTMRRD

>Bolivian squirrel monkey (*Saimiri boliviensis boliviensis*) A0A2K6V4A0

MEPRVRTVCFLFFLLWVLAEPAENSDFHLPGDYLLGGLFTLHANMKGIVHLNFLQVPMCKEYEVKLSGYNLMQAMRFAVEEINNDSSLLPDVRLGYEMVDVCYVSNNVQPVLYFLAQEDNLLPIQEDYSNYVPRVVAVIGPENSESVTTVANFLSLFLLPQITYSAISDQLRDKQRFPALLRTTPSAKHHIEAMVQLMLHFRWNWISVLVSSDTYGRDNGQLLGDRLAGGDICIAFQETLPTLQPNQDMMPEDRQRLVSIVEKLQQSTARVVVVFSPDLTLYDFFREVLRQNFTGAVWIASESWAIDPVLHNLTGLHRTGTFLGITLQNVPIPGFNEFRVRGPQAGPTHQRSTCNQECDTCLNSTLSFNTILRLSGERVVYSVYSAVYAVAHALHSLLGCDHSACTKRVVYPWQLLEEIWKVNFTLLDHQISFDPQGDVALHLEIVQWQWDLSQNFFQSVASYSPLQGHLKDIQDISWHTVNNTIPVSMCSKRCQSGQKKKPVGIHTCCFECIDCPPGTFLNQTANEYDCQACPSNEWSHQSETSCFKRRLSFLEWHEAATIAVALLAALGFLSTLAILVIFWRHFETPMVRSAGGPMCFLMLTLLLVAYMVVPVYVGLPKVSTCLCRQALFPVCFTICISCIAVRSFQIVCVFKMASRFPRAYSYWVRYQGSYVSVAFITALKMVTVVISLLATGLNPTTRTDTDDPKIMIISCNPNYRNSLLFNTSLDLLLSVAGFSFAYMGKELPTNYNEAKFITFSMTFYFTSSVSLCTFMSVYDGVLVTIVDLLVTVFNLLAISLGYFGPKCYMILFYPERNTPAYFNSMIQGYTMRRD

>Squirrel monkey (*Saimiri sciureus*) A3QP08

MEPRVRTVCFLFFLLRVLAEPAKNSDFYLPGDYLLGGLFTLHANMKGTVHLNFLQVPMCKEYEVKLSGYNLMQAMRFAVEEINNDSSLLPDVRLGYEMVDVCYVSNNVQPVLYFLAQEDNLLPIQEDYSNYVPRVVAVIGPENSESVTTVANFLSLFLLPQITYSAISDQLRDKQRFPALLRTTPSAKHHIEAMVQLMLHFRWNWISVLVSSDTYGRDNGQLLGDRLAGGDICIAFQETLPTLQPNQDMMPEDRQRLVSIVEKLQQSTARVVVVFSPDLTLYDFFREVLRQNFTGAVWIASESWAIDPVLHNLTGLHRTGTFLGITLQNVPIPGFNEFRVRGPQAGPTHQRSTCNQECDTCLNSTLSFNTILRLSGERVVYSVYSAVYAVAHALHSLLGCDHSACTKRGVYPWQLLEEIWKVNFSLLDHQIFFDPQGDVALHLEIVQWQWDLSQNPFQSVASYQPLQGHLKDIQDISWHTVNNTIPVSMCSKRCQSGQKKKPVGIHTCCFECIDCPPGTFLNQTANEYDCQACPSNEWSHQSETSCFKRRLSFLEWHEAATIAVALLAALGFLSTLAILVIFWRHFETPMVRSAGGPMCFLMLTLLLVAYMVVPVYVGLPKVSTCLCRQALFPVCFTICISCIAVRSFQIVCVFKMASRFPRAYSYWVRYQGSYVSVAFITALKMVTVVISLLATGLNPTTRTDTDDPKIMIISCNPNYRNSLLFNTSLDLLLSVAGFSFAYMGKELPTNYNEAKFITFSMTFYFTSSVSLCTFMSVYDGVLVTIVDLLVTVFNLLAISLGYFGPKCYMILFYPERNTPAYFNSMIQGYTMRRD

>Coquerel's sifaka (*Propithecus coquereli*) A0A2K6FEA4

MGRWARMICSLFLLLQVLVEPAENSDFYLTGDYLLGGLFSLHANVKGTVHLNYLQVPKCKEYEMKVLGYNLMQAMRFAVEEINNHSSLLPGVLLGYEMVDVCYISNNVQPVLYFLAQKDYFLPIQEDYSSYVPRVVAVIGPDNSESVVTVANFLSLFLLPQITYSAISDQLRNKHRFPALLRTMPSADHHVEAIAQLMLHFRWNWIVVLVSSDDYGRDNGQRLSERLTGRDICIAFQESLPAPQPNQVATPEEHSRLGAIVDKLQQSTARVVVVFSPDLALHNFFREVLRRNFTGVVWIASESWAIDPVLHSFTELRHAGTFLAVTTQSVPIPGFSEFRVRRWQAGPPSGRNSQGSTCNQECDECFNTTKSFNTILTLSGERVVYGVYSAVYAVAHALHSLLGCGETNCTKEVVYPWQLLKEIWKVNFTLLGHQIIFDHEGDMAMPLDIIQWQWNMSQSPFQSIASYYPAQRQLKNISYISWHTPNNTVPVSMCSKSCQPGQRKKPVGTHSCCFECIDCLPGTFLNQTADEYDCQSCPNNKWSHRSGTSCFRRRLAFLEWHEAPTIIVAMLAALGFFSTLAIMVIFWRHFQTPMVRSAGGPMCFLMLTPLLVAYMVVPVYVGSPTVSTCLCRQTLFPICFTICISCITVRSFQIVCIFKMASRLPRAYSYWVRYHGPYVSVGFITALKVATVVGILLTTSPNPTTRADPDDPQIMILSCNPNFRGLLLFSTGLDLALSVVGFSFAYMGKELPTNYNEAKFITLSMTFYFTSSVSLCTFMSVYSGVLVTIMDLLVTVLNLLAISLGYFGPKCYMILFYPERNTQAYFNSMIQGYTMRKD

>Gray mouse lemur (*Microcebus murinus*) XP_012609522

MGPWARMVCSLSLLLQVPAGPAESSDFYLAGDYLLGGLFTLHANVKGTVHLNYLQVPKCKEYEMKVLGYNLMQAMRFAVEEINNHSSLLPGVQLGYEMVDVCYISNNVQPVLYFLAREDYFLPIKEDYSQYVPRVVAVIGPDNSESVVTVANFLSLFLLPQITYSAISDQLRDKDRFPALLRTMPSADHHVEAIAQLLLHFRWNWIVVLVSSDDYGRDNGQRLSERLTGRDICIAFQESLPTPQPNQVMTTEEHRRLRAIVDKLQQTSARVVVVFSPDLALHNFFHEVVRWNFTGAVWIASESWAIDPVLHNLTELRHTGTFLGVTIQNVPIPGFSEFRMRRWQAGSPPSRNGRGATCNQECDKCFNTTETFNEVLTLSGERIVYSVYSAVYAVAHALHSLLGCGETGCTKEVVYPWQLLKEIWKVNFTLLGHQIAFDKDGDMPMPLEVVQWQWDLSQSPFQSVASYYPLQRQLTNIGHISWHTPNNMVPVSMCSKHCQPGQRKKPVGIHPCCFECIDCLPGTFLNQTADEYDCQPCPSNKWSHRNGTSCFKRRLAFLEWHEAPTIIVAMLAALGFLSTLAILAIFWRHLQTPMVRSAGGPMCFLMLAPLLVSYMVVPVYIGPPTAATCLCRQTLFPICFTVCISCITVRSFQIVYVFKMASHLPRAYGYWVRYHGPCVSVAFITSLKVAIVVGNLLTTSPSPTTRADPDDPQIMILSCNPSFRGLLMISTGLDLALSVVGFSFAYMGKELPTNYNEAKFITLSMTFYFTSSVSLCTFMSVYSGVLVTIMDLLVTVLNLLAISLGYFGPKCYMILFYPERNTQAYFNSMIQGYTMRKD

>Ring-tailed lemur (*Lemur catta*) XP_045402430

MGPWARVFCSLFLLLQVLAEPAGNSDFYLAGDYLLGGLFTLHANVKGIVHLNYLQVPKCKEYEMKVLGYNLMQAMRFAVEEINNHSSLLPGVVLGYEMVDVCYVSNNVQPVLYFLAQDNYFLPIQEDYSQYVPRVVAVIGPDNSEAVVTVANFLSLFLLPQITYSAISDQLRDKQRYPALLRTMPSADHHVEAIAQLMLYFRWNWIIVLVSSDDYGRDNGQRLSERLTGHDICIAFQESLPVPQPNQVVTPEEHSRLGAIVEKLQQSSARVVVVFSPDLALHNFFLEVMRRNYTGAVWIASESWAIDPVLHNLIELRHTGTFLGVTTQNVPIPSFSEFRVRSRRAGPPPRRSSQASTCNQECDECFNTTENYNNVLTLSGERVVYSVYTAVYAVAHALHNLLGCSETGCTKKVVYPWQLLKELWKVNFTLLGHQIKFNQEGDMPIPLEVIQWQWGMNQNPFQSVASYYPTQRQLTNIGSISWHTPNNTVPLSMCSRSCHPGQRKKSVGIHPCCFECIDCLPGTFLNQSADEYDCQPCPSTKWSHRNATSCFKRRLAFLEWHEAPTIFVALLAALGFLSTLAILLIFWRHFQTPVVRSAGGPMCFLMLTPLLVAYTVVPVYVGLPTVSTCLWRLTVFPICFTVCISCITVRSFQIVYIFKMASRLPRAYSYWVRYHGPYVSVVVTTALKVAIVVGNLLTTSPSPTARADPDDPQVMILSCNPNFRSLLLVNAGLDLALSVAGFGFAYVGKELPTNYNEAKFITLSMTFYFTSSVSLCTFMSVYSGVLVTIMDLLVTVLNLLAISLGYFGPKCYMILFYPERNTQAYFNSMIQGYTMGKD

Protein sequences of T1R3s in primates analyzed in this study (Common name, scientific name and Genbank accession number of the species are annotated respectively in the FASTA format):

>Human (*Homo sapiens*) Q7RTX0

MLGPAVLGLSLWALLHPGTGAPLCLSQQLRMKGDYVLGGLFPLGEAEEAGLRSRTRPSSPVCTRFSSNGLLWALAMKMAVEEINNKSDLLPGLRLGYDLFDTCSEPVVAMKPSLMFLAKAGSRDIAAYCNYTQYQPRVLAVIGPHSSELAMVTGKFFSFFLMPQVSYGASMELLSARETFPSFFRTVPSDRVQLTAAAELLQEFGWNWVAALGSDDEYGRQGLSIFSALAAARGICIAHEGLVPLPRADDSRLGKVQDVLHQVNQSSVQVVLLFASVHAAHALFNYSISSRLSPKVWVASEAWLTSDLVMGLPGMAQMGTVLGFLQRGAQLHEFPQYVKTHLALATDPAFCSALGEREQGLEEDVVGQRCPQCDCITLQNVSAGLNHHQTFSVYAAVYSVAQALHNTLQCNASGCPAQDPVKPWQLLENMYNLTFHVGGLPLRFDSSGNVDMEYDLKLWVWQGSVPRLHDVGRFNGSLRTERLKIRWHTSDNQKPVSRCSRQCQEGQVRRVKGFHSCCYDCVDCEAGSYRQNPDDIACTFCGQDEWSPERSTRCFRRRSRFLAWGEPAVLLLLLLLSLALGLVLAALGLFVHHRDSPLVQASGGPLACFGLVCLGLVCLSVLLFPGQPSPARCLAQQPLSHLPLTGCLSTLFLQAAEIFVESELPLSWADRLSGCLRGPWAWLVVLLAMLVEVALCTWYLVAFPPEVVTDWHMLPTEALVHCRTRSWVSFGLAHATNATLAFLCFLGTFLVRSQPGCYNRARGLTFAMLAYFITWVSFVPLLANVQVVLRPAVQMGALLLCVLGILAAFHLPRCYLLMRQPGLNTPEFFLGGGPGDAQGQNDGNTGNQGKHE

>Western lowland gorilla (*Gorilla gorilla gorilla*) Q717C1

MLGPAVLGLSLWALLQPGAGAPLCLSQQLRMKGDYMLGGLFPLGEAEEAGFRSRTRPSSPVCTRFSSNGLLWALAMKMAVEEINNKSDLLPGLRLGYDLFDTCSEPVVAMKPSLMFLAKAGSRDIAAYCNYTQYQPRVLAVIGPHSSELAMVTGKFFSFFLMPQVSYGASMELLSARETFPSFFRTVPSDRVQLTAAAELLQEFGWNWVAALGSDDEYGRQGLSIFSALAAARGICIAHEGLVPLPRADDSRLGKVQDVLHQVNQSSVQVVLLFASVHAAHALFNYSISSRLSPKVWVASEAWLTSDLVMGLPGMAQMGTVLGFLQRGAQLHEFPQYVKTHLALAADPAFCSALGEREQGLEEDVVGQRCPQCDCITLQNVSAGLNHHQTFSVYAAVYSVAQALHNTLQCNASGCPAQDPVKPWQLLENMYNLTFHAGGLMLRFDSSGNVDMEYDLKLWVWQGSVPRLHDVGRFNGSLRTERLKIRWHTSDNQKPVSRCSRQCQEGQVRRVKGFHSCCYDCVDCEAGSYRQNPDDVTCTSCGQDEWSPERSTRCFHRRSRFLAWGEPAVLLLLLLLSLALGLVLAALGLFVHHRDSPLVQASGGPLACFGLVCLGLVCLSVLLFPGQPSPAQCLAQQPLSHLPLTGCLSTLFLQAAEIFVESELPLSWADRLSGCLRGPWAWLVVLLAMLVEVALCTWYLVAFPPEVVTDWHMLPTEALVHCRTRSWVSFGLAHATNATLAFLCFLGTFLVRSQPGRYNRARGLTFAMLAYFITWVSFVPLLANVQVVLRPAVQMGALLLCVLGILAAFHLPRCYLLIRQPGLNTPEFFLGGGPGDAQGRNDGDTGNQGKHE

>Sumatran orangutan (*Pongo abelii*) BCT43468

MLGPAVLGLSLWALLHSGTGAPLCLSQQLRMKGDYVLGGLFPLGEAEEAGLRSRTRPSSPVCTRFSSNGLLWALAMKMAVEEINNKSDLLPGLRLGYDLFDTCSEPVVAMKPSLMFLAKADSRDIAAYCNYTQYQPRVLAVIGPHSSELALVTGKFFSFFLMPQVSYGASMELLSARETFPSFFRTVPSDRVQLTAAAELLQQFGWNWVAALGSDDEYGRQGLSIFSALAAARGICIAHEGLVPLPRADDLRLGKVQDVLHQVNQSSVQVVLLFASVHAAYALFNYSISSRLSPKVWVASEAWLTSDLIMGLPGMAQVGTVLGFLQKGAQLHEFSQYVKTHLALAADPAFCAALGEREQGLEEDVVGQRCPQCDCITLQNVSAGLNHHQMFSVYAAVYSVAQALHNTLQCNASGCPAQDPVKPWQLLENMYNLTFHVGGLTLRFNSSGNVDMEYDLKLWVWQGSVPKLHNVGGFNGSLWTERLKIRWHTPDNQKPVSQCSRQCQEGQVRRVKGFHSCCYDCVDCKAGSYRHSPDDLACTFCRQDEWSPERSTRCFRRRSRFLAWGEPAVLLLLLLLSLALGLVLAALGLFIRHRDSPLVRASGGPLACFGLVCLGLVCLSVLLFPGRPGTARCLAQQPLSHLPLTGCLSTLFLQAAEIFVESELPLSWADRLSGCLRGPWAWLVVLLAMLVEVALCTWYLVAFPPEVVTDWHILPTEALVHCRTRSWVSFGLAHATNATLAFLCFLGTFLVQSRPGRYNRARGLTFAMLAYFITWVSFVPLLANVQVVLRPAVQMGALLLCVLGILAAFHLPRCYLLMRQPGLNTPEFFLGGGPGDAQGRNDGDTGNQRKHE

>Bornean orangutan (*Pongo pygmaeus*) A2T0N1

MLGPAVLGLSLWALLHSGTGAPLCLSQQLRMKGDYVLGGLFPLGEAEEAGLRSRTRPSSPVCTRFSSNGLLWALAMKMAVEEINNKSDLLPGLRLGYDLFDTCSEPVVAMKPSLMFLAKADSRDIAAYCNYTQYQPRVLAVIGPHSSELALVTGKFFSFFLMPQVSYGASMELLSARETFPSFFRTVPSDRVQLTAAADLLQQFGWNWVAALGSDDEYGRQGLSIFSALAAARGICIAHEGLVPLPRADDLRLGKVQDVLHQVNQSSVQVVLLFASVHAAYALFNYSISSRLSPKVWVASEAWLTSDLIMGLPGMAQVGTVLGFLQKGAQLHEFSQYVKTHLALAADPAFCAALGEREQGLEEDVVGQRCPQCDCITLQNVSAGLNHHQMFSVYAAVYSVAQALHNTLQCNASGCPAQDPVKPWQLLENMYNLTFHVGGLTLRFNSSGNVDMEYDLKLWVWQGSVPKLHNVGGFNGSLWTERLKIRWHTPDNQKPVSQCSRQCQEGQVRRVKGFHSCCYDCVDCKAGSYRHSPDDLACTFCRQDEWSPERSTRCFRRRSRFLAWGEPAVLLLLLLLSLALGLVLAALGLFIRHRDSPLVRASGGPLACFGLVCLGLVCLSVLLFPGRPGTARCLAQQPLSHLPLTGCLSTLFLQAAEIFVESELPLSWADRLSGCLRGPWAWLVVLLAMLVEVALCTWYLVAFPPEVVTDWHILPTEALVHCRTRSWVSFGLAHATNATLAFLCFLGTFLVQSRPGRYNRARGLTFAMLAYFITWVSFVPLLANVQVVLRPAVQMGALLLCVLGILAAFHLPRCYLLMRQPGLNTPEFFLGGGPGDAQGRNDGDTGNQRKHE

>Pygmy chimpanzee (*Pan paniscus*) A0A2R9B4E3

MLGPAVLGLSLWALLHPGTGAPLCLSQQLRMKGDYVLGGLFPLGEAEEAGLRSRTRPSSPVCTRFSSNGLLWALAMKMAVEEINNKSDLLPGLRLGYDLFDTCSEPVVAMKPSLVFLAKAGSRDIAAYCNYTQYQPRVLAVIGPHSSELAMVTGKFFSFFLMPQVSYGASMELLSARETFPSFFRTVPSDRVQLTAAAELLQEFGWNWVAALGSDDEYGRQGLSIFSALAAARGICIAHEGLVPLPRADDSRLGKVQDVLHQVNQSSVQVVLLFASVHAAHALFNYSISSRLSPKVWVASEAWLTSDLVMGLPGMAQMGTVLGFLQRGAQLHEFPQYVKTHLALAADPAFCSSLGEREQGLEEDVVGQRCPQCDCITLQNVSAGLNHHQTFSVYAAVYSVAQALHNTLQCNASGCPAQDPVKPWQLLENMYNLTFHAGGLMMRFDSSGNVDMEYDLKLWVWQGSVPRLHDVGRFNGSLRTERLKIHWHTSDNQKPVSRCSRQCQEGQVRRVKGFHSCCYDCVDCEAGSYRQNPDDIACTFCGQDEWSPERSTRCFRRRSRFLVWGEPAVLLLLLLLSLALGLVLAALGLFIHHRDSPLVQASGGPLACFGLVCLGLVCLSVLLFPGQPSPARCLAQQPLSHLPLTGCLSTLFLQAAEIFVESELPLSWADRLSGCLRGPWAWLVVLLAMLVEVALCTWYLVAFPPEVVTDWHMLPTEALVHCRTRSWVSFGLAHATNATLAFLCFLGTFLVRSQPGRYNRARGLTFAMLAYFITWVSFVPLLANVQVVLRPAVQMGALLLCVLGILAAFHLPRCYLLMWQPGLNTPEFFLGGGPGDAQGRNDGDTGNQGKHE

>Chimpanzee (*Pan troglodytes*) Q717C2

MLGPAVLGLSLWALLHPGTGAPLCLSQQLRMKGDYVLGGLFPLGEAEEAGLRSRTRPSSPVCTRFSSNGLLWALAMKMAVEEINNKSDLLPGLRLGYDLFDTCSEPVVAMKPSLVFLAKAGSRDIAAYCNYTQYQPRVLAVIGPHSSELAMVTGKFFSFFLMPQVSYGASMELLSARETFPSFFRTVPSDRVQLTAAAELLQEFGWNWVAALGSDDEYGRQGLSIFSALAAARGICIAHEGLVPLPRADDSRLGKVQDVLHQVNQSSVQVVLLFASVHAAHALFNYSISSRLSPKVWVASEAWLTSDLVMGLPGMAQMGTVLGFLQRGAQLHEFPQYVKTHLALAADPAFCSALGEREQGLEEDVVGQRCPQCDCITLQNVSAGLNHHQTFSVYAAVYSVAQALHNTLQCNASGCPAQDPVKPWQLLENMYNLTFHAGGLMLRFDSSGNVDMEYDLKLWVWQGSVPRLHDVGRFNGSLRTERLKIRWHTSDNQKPVSRCSRQCQEGQVRRVKGFHSCCYDCVDCEAGSYRQNPDDIACTFCGQDEWSPERSTRCFRRRSRFLAWGEPAVLLLLLLLSLALGLVLAALGLFIHHRDSPLVQASGGPLACFGLVCLGLVCLSVLLFPGQPSPARCLAQQPLSHLPLTGCLSTLFLQAAEIFVESELPLSWADRLSGCLRGPWAWLVVLLAMLVEVALCTWYLVAFPPEVVTDWHMLPTEALVHCRTRSWVSFGLAHATNATLAFLCFLGTFLVRSQPGRYNRARGLTFAMLAYFITWVSFVPLLANVQVVLRPAVQMGALLLCVLGILAAFHLPRCYLLMWQPGLNTPEFFLGGGPGDAQGRNDGDTGNQGKHE

>Northern white-cheeked gibbon (*Nomascus leucogenys*) A0A0M4P6G3

MLGPAVLGLSLWALLHPGTGAPLCLSQQLRMKGDYVLGGLFPLGEAEEAGIRSRTRPSSPVCTRFSSNGLLWALAMKMAVEEINNKSDLLPGLRLGYDLFDTCSEPVVAMKPSLMFLARADSRDIAAYCNYTQYQPRVLAVIGPHSSELALVTGKFFSFFLMPQVSYGASMELLSARETFPSFFRTVPSDRVQLTAAAELLQEFGWNWVAALGSDDEYGRQGLSIFSALAAARGICIAHEGLVPLPRADDLRLGKVQDVLHQVNQSNVQVVLLFASVHAAHALFNYSISSKLSPKVWVASEAWLTSDLVMGLPGMAQVGTVLGFLQRGAQLHEFSQYVKTHLALAADPAFCATLGKREQVLEEDVVGQRCPQCDCITLQNVSAGLNHHQTFSVYAAVYSVAQALHNTLQCNASGCPTQDPVKPWQLLENMYNLTFHAGGLTLRFNSSGNVDMEYDLKLWVWQGSVPKLHNVGRFNGSLWTEHLKIRWHTLDNQKPVSQCSQQCQEGQVRRVKGFHSCCYDCVDCKAGSYRHSPDDLACTFCRQDEWSPERSTRCFRRRYRFLAWGEPAVLLLLLLLSLALGLVLAALGLFIRHRDSPLVQASGGPLACFGLVCLGLVCLSVLLFPGQPSPARCLAQQPLSHLPLTGCLSTLFLQAAEIFVESELPLSWADRLSGCLRGPWAWLVVLLAMLVEAALCTWYLVAFPPEVVTDWRMLPKEALVHCRTRSWVSFGLAHATNATLAFLCFLGTFLVRSQPGRYNRARGLTFAMLAYFITWVSFVPLLANVQVVLRPAVHMGALLLCVLGILAAFHLPRCYLLMQQPGLNTPEFFLGGGPGDAQGRNDGDTGNQGKHE

>Northern buffed-cheeked gibbon (*Nomascus annamensis*) A0A0M4P9W8

MLGPAVLGLSLWALLHPGTGAPLCLSQQLRMKGDYVLGGLFPLGEAEEAGIRSRTRPSSPVCTRFSSNGLLWALAMKMAVEEINNKSDLLPGLRLGYDLFDTCSEPVVAMKPSLMFLARADSRDIAAYCNYTQYQPRVLAVIGPHSSELALVTGKFFSFFLMPQVSYGASMELLSARETFPSFFRTVPSDRVQLTAAAELLQEFGWNWVAALGSDDEYGRQGLSIFSALAAARGICIAHEGLVPLPRADDLRLGKVQDVLHQVNQSNVQVVLLFASVHAAHALFNYSISSKLSPKVWVASEAWLTSDLVMGLPGMAQVGTVLGFLQRGAQLHEFSQYVKTHLALAADPAFCATLGKREQVLEEDVVGQRCPQCDCITLQNVSAGLNHHQTFSVYAAVYSVAQALHNTLQCNASGCPTQDPVKPWQLLENMYNLTFHAGGLTLRFNSSGNVDMEYDLKLWVWQGSVPKLHNVGRFNGSLWTEHLKIRWHTLDNQKPVSQCSQQCQEGQVRRVKGFHSCCYDCVDCKAGSYRHSPDDLACTFCRQDEWSPERSTRCFRRRYRFLAWGEPAVLLLLLLLSLALGLVLAALGLFIRHRDSPLVQASGGPLACFGLVCLGLVCLSVLLFPGQPSPARCLAQQPLSHLPLTGCLSTLFLQAAEIFVESELPLSWADRLSGCLRGPWAWLVVLLAMLVEAALCTWYLVAFPPEVVTDWRMLPKEALVHCRTRSWVSFGLAHATNATLAFLCFLGTFLVQSQPDRYNRARGLTFAMLAYFITWVSFVPLLANVQVVLRPAVHMGALLLCVLGILAAFHLPRCYLLMQQPGLNTPEFFLGGGPGDAQGRNNGDTGNQGKHE

>Western hoolock gibbon (*Hoolock hoolock*) A0A0M4PYM9

MLGPAVLGLSLWALLHPGMGAPLCLSQQLRMKGDYVLGGLFPLGEAEEAGIHSRTRPSSPVCTRFSSNGLLWALAMKMAVEEINNKSDLLPGLRLGYDLFDTCSEPVVAMKPSLMFLARADSRDIAAYCNYTQYQPRVLAVIGPHSSELALVTGKFFSFFLMPQVSYGASMELLSARETFPSFFRTVPSDRVQLTAAAELLQEFGWNWVAALGSDDEYGRQGLSIFSALAAARGICIAHEGLVPLPHADDLRLGKVQDVLHQVNQSNVQVVLLFASVHAAHALFNYSISSRLSPKVWVASEAWLTSDLVMGLPGMAQVGTVLGFLQRGAQLHEFSQYVKTHLALAADPAFCATLGKREQVLEEDVVGQRCPQCDCITLQNVSAGLNHHQTFSVYAAVYSVAQALHNTLQCNASGCPTQDPVKPWQLLENMYNLTFHAGGLTLRFNSSGNVDMEYDLKLWVWQGSVPKLHNVGRFNGSLWTEHLKIRWHTLDNQKPVSQCSQQCQEGQVRRVKGFHSCCYDCVDCKAGSYRHSPDDLACTFCRQDEWSPERSTRCFRRRYRFLAWGEPAVLLLLLLLSLALGLVLAALGLFIRHRDSPLVQASGGPLACFGLVCLGLVCLSVLLFPGQPSPARCLAQQPLSHLPLTGCLSTLFLQAAEIFVESELPLSWADRLSGCLRGPWAWLVVLLAMLVEAALCTWYLVAFPPEVVTDWRMLPKEALVHCRTRSWVSFGLAHATNATLAFLCFLGTFLVQSQPGRYNRARGLTFAMLAYFITWVSFVPLLANVQVVLRPAVHMGALLLCVLGILAAFHLPRCYLLMQQPGLNTPKFFLGGGPGDAQGRNDGDTGNQGKHE

>Hylobates muelleri abbotti A0A0M5LHY7

MLGPAVLGLSLWALLHPGTGAPLCLSQQLRMKGDYMLGGLFPLGEAEEAGIRSRTRPSSPVCTRFSSNGLLWALAMKMAVEEINNKSDLLPGLRLGYDLFDTCSEPVVAMKPSLMFLARADSRDIAAYCNYTQYQPRVLAVIGPHSSELALVTGKFFSFFLMPQVSYGASMELLSARETFPSFFRTVPSDRVQLTAAAELLQQFGWNWVAALGSDDEYGRQGLSIFSALAAARGICIAHEGLVPLPRADDLRLGKVQDVLHQVNQSNVQVVLLFASVHAAHALFNYSISSRLSPKVWVASEAWLTSDLVMGLPGMAQVGTVLGFLQRGAQLHEFSQYVKTHLALAADPAFCATLGKREQVLEEDVVGQRCPQCDCITLQNVSAGLNHHQTFSVYAAVYSVAQALHNTLQCNASGCPTQDPVKPWQLLENMYNLTFHAGGLTLRFNSSGNVDMEYDLKLWVWQGSVPKLHNVGRFNGSLWTEHLKIRWHTLDNQKPVSQCSQQCQEGQVRRVKGFHSCCYDCVDCKAGSYRHSPDDLACTFCRQDEWSPERSTRCFRRRYRFLAWGEPAVLLLLLLLSLALGLVLAALGLFIRHRDSPLVQASGGPLACFGLVCLGLVCLSVLLFPGQPSPARCLAQQPLSHLPLTGCLSTLFLQAAEIFVESELPLSWADRLSGCLRGPWAWLVVLLAMLVEAALCTWYLVAFPPEVVTDWRMLPKEALVHCRTRSWVSFGLAHATNATLAFLCFLGTFLVQSQPGRYNRARGLTFAMLAYFITWVSFVPLLANVQVVFRPAVHMGALLLCVLGILVAFHLPRCYLLMQQPGLNTPEFFLGGGPGDAQGRNDGDTGNQGKHE

>Agile gibbon (*Hylobates agilis*) A0A0M4Q0P2

MLGPAVLGLSLWALLHPGTGAPLCLSQQLRMKGDYMLGGLFPLGEAEEAGIRSRTRPSSPVCTRFSSNGLLWALAMKMAVEEINNKSDLLPGLRLGYDLFDTCSEPVVAMKPSLMFLARADSRDIAAYCNYTQYQPRVLAVIGPHSSELALVTGKFFSFFLMPQVSYGASMELLSARETFPSFFRTVPSDRVQLTAAAELLQQFGWNWVAALGSDDEYGRQGLSIFSALAAARGICIAHEGLVPLPRADDLRLGKVQDVLHQVNQSNVQVVLLFASVHAAHALFNYSISSRLSPKVWVASEAWLTSDLVMGLPGMAQVGTVLGFLQRGAQLHEFSQYVKTHLALAADPAFCATLGKREQVLEEDVVGQRCPQCDCITLQNVSAGLNHHQTFSVYAAVYSVAQALHNTLQCNASGCPTQDPVKPWQLLENMYNLTFHAGGLTLRFNSSGNVDMEYDLKLWVWQGSVPKLHNVGRFNGSLWTEHLKIRWHTLDNQKPVSQCSQQCQEGQVRRVKGFHSCCYDCVDCKAGSYRHSPDDLACTFCRQDEWSPERSTRCFRRRYRFLAWGEPAVLLLLLLLSLALGLVLAALGLFIRHRDSPLVQASGGPLACFGLVCLGLVCLSVLLFPGQPSPARCLAQQPLSHLPLTGCLSTLFLQAAEIFVESELPLSWADRLSGCLRGPWAWLVVLLAMLVEAALCTWYLVAFPPEVVTDWRMLPKEALVHCRTRSWVSFGLAHATNATLAFLCFLGTFLVQSQPGRYNRARGLTFAMLAYFITWVSFVPLLANVQVVFRPAVHMGALLLCVLGILAAFHLPRCYQLMQQPGLNTPEFFLGGGPGDAQGRNDGDTGNQGKHE

>Gibbon (*Hylobates lar*) A0A0M3TYD2

MLGPAVLGLSLWALLHPGTGAPLCLSQQLRMKGDYMLGGLFPLGEAEEAGIRSQTRPSSPVCTRFSSNGLLWALAMKMAVEEINNKSDLLPGLRLGYDLFDTCSEPVVAMKPSLMFLARADSRDIAAYCNYTQYQPRVLAVIGPHSSELALVTGKFFSFFLMPQVSYGASMELLSARETFPSFFRTVPSDRVQLTAAAELLQQFGWNWVAALGSDDEYGRQGLSIFSALAAARGICIAHEGLVPLPRADDLRLGKVQDVLHQVNQSNVQVVLLFASVHAAHALFNYSISSRLSPKVWVASEAWLTSDLVMGLPGMAQVGTVLGFLQRGAQLHEFSQYVKTHLALAADPAFCATLGKREQVLEEDVVGQRCPQCDCITLQNVSAGLNHHQTFSVYAAVYSVAQALHNTLQCNASGCPTQDPVKPWQLLENMYNLTFHAGGLTLRFNSSGNVDMEYDLKLWVWQGSVPKLHNVGRFNGSLWTEHLKIRWHTLDNQKPVSQCSQQCQEGQVRRVKGFHSCCYDCVDCKAGSYRHSPDDLACTFCRQDEWSPERSTRCFRRRYRFLAWGEPAVLLLLLLLSLALGLVLAALGLFIRHRDSPLVQASGGPLACFGLVCLGLVCLSVLLFPGQPSPARCLAQQPLSHLPLTGCLSTLFLQAAEIFVESELPLSWADRLSGCLRGPWAWLVVLLAMLVEAALCTWYLVAFPPEVVTDWRMLPKEALVHCRTRSWVSFGLAHATNATLAFLCFLGTFLVQSQPGRYNRARGLTFAMLAYFITWVSFVPLLANVQVVLRPAVHMGALLLCVLGILAAFHLPRCYLLMQQPGLNTPEFFLGGGPGDAQGRNDGDTGNQGKHE

>Pileated gibbon (*Hylobates pileatus*) A0A0M4P9H4

MLGPAVLGLSLWALLHPGTGAPLCLSQQLRMKGDYMLGGLFPLGEAEEAGIRSRTRPSSPVCTRFSSNGLLWALAMKMAVEEINNKSDLLPGLRLGYDLFDTCSEPVVAMKPSLMFLARADSRDIAAYCNYTQYQPRVLAVIGPHSSELALVTGKFFSFFLMPQVSYGASMELLSARETFPSFFRTVPSDRVQLTAAAELLQQFGWNWVAALGSDDEYGRQGLSIFSALAAARGICIAHEGLVPLPRADDLRLGKVQDVLHQVNQSNVQVVLLFASVHAAHALFNYSISSRLSPKVWVASEAWLTSDLVMGLPGMAQVGTVLGFLQRGAQLHEFSQYVKTHLALAADPAFCATLGKREQVLEEDVVGQRCPQCDCITLQNVSAGLNHHQTFSVYAAVYSVAQALHNTLQCNASGCPTQDPVKPWQLLENMYNLTFHAGGLTLRFNSSGNVDMEYDLKLWVWQGSVPKLHNVGRFNGSLWTEHLKIRWHTLDNQKPVSQCSQQCQEGQVRRVKGFHSCCYDCVDCKAGSYRHSPDDLACTFCRQDEWSPERSTRCFRRRYRFLAWGEPAVLLLLLLLSLALGLVLAALGLFIRHRDSPLVQASGGPLACFGLVCLGLVCLSVLLFPGQPSPARCLAQQPLSHLPLTGCLSTLFLQAAEIFVESELPLSWADRLSGCLRGPWAWLVVLLAMLVEAALCTWYLVAFPPEVVTDWRMLPKEALVHCRTRSWVSFGLAHATNATLAFLCFLGTFLVQSQPGRYNRARGLTFAMLAYFITWVSFVPLLANVQVVLRPAVHMGALLLCVLGILAAFHLPRCYLLMQQPGLNTPEFFLGGGPGDAQGRNDGDTGNQGKHE

>Siamang (*Symphalangus syndactylus*) A0A0M4NXW8

MLGPAVLGLSLWALLHPGTGAPLCLSQQLRMKGDYMLGGLFPLGEAEEAGIRSRTRPSSPVCTRFSSNGLLWALAMKMAVEEINNKSDLLPGLRLGYDLFDTCSEPVVAMKPSLMFLARADSRDIAAYCNYTRYQPRVLAVIGPHSSELALVTGKFFSFFLMPQVSYGASMELLSARETFPSFFRTVPSDRVQLTAAAELLQEFGWNWVAALGSDDEYGRQGLSIFSALAAARGICIAHEGLVPLPRADDLRLGKVQDVLHQVNQSNVQVVLLFASVHAAHALFNYSISSRLSPKVWVASEAWLTSDLVMGLPGMAQVGTVLGFLQRGAQLHEFSQYVKTHLALAADPAFCATLGKREQVLEEDVVGQRCPQCDCITLQNVSTGLNHHQTFSVYAAVYSVAQALHNTLQCNASGCPTQDPVKPWQLLENMYNLTFHAGGLTLRFNSSGNVDMEYDLKLWVWQGSVPKLHNVGRFNGSLWTERLKIRWHTLDNQKPVSQCSQQCQEGQVRRVKGFHSCCYDCVDCKAGSYRHSPDDLACTFCRQDEWSPERSTRCFRRRYRFLAWGEPAVLLLLLLLSLALGLVLAALGLFIRHRDSPLVQASGGPLACFGLVCLGLVCLSVLLFPGQPSPARCLAQQPLSHLPLTGCLSTLFLQAAEIFVESELPLSWADRLSGCLRGPGAWLVVLLAMLVEAALCTWYLVAFPPEVVTDWRMLPKEALVHCRTRSWVSFGLAHATNATLAFLCFLGTFLVQSQPGRYNRARGLTFAMLAYFITWVSFVPLLANVQVVLRPAVHMGALLLCVLGILAAFHLPRCYLLMQQPGLNTPEFFLGGGPGDAQGRNDGDTGNQGKHE

>Green monkey (*Chlorocebus sabaeus*) ALE59905

MLHPAVLGLSLWALLHLGTGAPLCLSQQLRMKGDYVLGGLFPLGEAEEAGLGSRTRPSSPVCTRFSSNGLLWALAMKMAVEEINNRSDLLPGLRLGHDLFDTCSEPVVAMKPSLMFLAKADSRNIAAYCNYTQYQPRVLAVIGPHSSELAVVTGKFFGFFLMPQVSYGASMELLSARETFPSFFRTVPSDRVQLTAAAELLQEFGWNWVAALGSDDEYGRQGLSIFSALAAARGICIAHEGLVPLPRANSLLLGKVQEVLHQVNQSSVQVVLLFASARAAHALFSYSISSKLSRKVWVASEAWLTSDLVMGLPGMAQVGTVLGFLQRGAQLHKFSQYVKTRLALAADPVFCTALGEREQGLEEDVVGRRCPQCDCITLQNVSAGLNHHQTFSVYAAVYSVAQALHNTLQCNASGCPMRDP

VKPWQLLENMYNLTFHAGGLTLRFNSNGNVDMEYDLKLWVWQGPVPELHDVGRFNGSLWIDSLKIHWHTSNNQKPVSQCSRQCQEGQVRRVKGFHSCCYDCVDCKAGSYRKSPDDLACTFCSQDEWSPERSTRCFRRRLRFLAWGEPAVLLLLLLLGLALGLVLAALGLFIRHRDSPLVQASGGPLACFGLVCLGLVCISVLLFPGQPSPARCLAQQPSSHLPLTGCLSTLILQAAEIFVESELPLSWADRLSGCLRGPWAWLVVLLAVLVEAALCAWYLMVFPPEVVTDWRMLPTEALVHCRARSWVSFGLVHATNATLAFLCFLGTFLVQSRPGRYNRARGLTFAMLAYFITWVSFVPLLANVQVVLRPAVQMGALLLCVLGILAAFHLPRCYLLVRQPELNTPEFFLGRGPGDARDRNDGDTGNQGKHE

>Golden-bellied mangabey (*Cercocebus chrysogaster*) ALE59897

MLRPAVLGLSLWAVLHLGTGAPLCLSQQLRMKGDYVLGGLFPLGEAEEAGLGSRTRPSSPVCTRFSSNGLLWALAMKMAVEEINNRSDLLPGLRLGHDLFDTCSEPVVAMKPSLMFLAKADSRDIAAYCNYTQYQPRVLAVIGPHSSELAVVTGKFFGFFLMPQVSYGASMELLSARETFPSFFRTVPSDRVQLTAAAELLQEFGWNWVAALGSDDEYGRQGLSIFSALAAARGICIAHEGLVPLPRANSPLLGKVQEVLHQVNQSSVQVVLLFASARAAHALFSYSISSKLSRKVWVASEAWLTSDLVMGLPGMAQVGTVLGFLQRGAQLHKFSQYVKTRLALAADPAFCAALGEREQGLEEDVVGRRCPQCDCITLQNVSAGLNHHQTFSVYAAVYSVAQALHNTLHCNASGCPVQEPVKPWQLLDNMYNLTFHAGGLTLRFNSNGNVDMEYDLKLWVWQGPVPELHNVGRFNGSLWIDSLKIRWHTSNNQKPVSQCSRQCQEGQVRRVKGFHSCCYDCVDCKAGSYRKSPDDLACTFCSQDEWSPERSTRCFRRRLRFLAWGEPAVLLLLLLFGLALGLVLAALGLFIRHRDSPLVQASGGPLACFGLVCLGLVCISVLLFPGQPSPARCLAQQPSSHLPLTGCLSTFILQAAEIFMESELPLSWADRLSGCLRGPWAWLVVLLAMLVEAALCAWYLVAFPPEVVTDWRMLPTEALVHCRARSWVSFGLVHATNATLAFLCFLGTFLVQSRPGRYNRARGLTFAMLAYFITWVSFVPLLANVQVVFRPAVQMGALLLCVLGILAAFHLPRCYLLVRQPELNTPEFFLGRGPGDARDRNDGHTGNQGKHE

>Sooty mangabey (*Cercocebus atys*) XP_011886488.1

MLRPAVLGLSLWAVLHLGTGAPLCLSQQLRMKGDYVLGGLFPLGEAEEAGLGSRTRPSSPVCTRFSSNGLLWALAMKMAVEEINNRSDLLPGLRLGHDLFDTCSEPVVAMKPSLMFLAKADSRDIAAYCNYTQYQPRVLAVIGPHSSELAVVTGKFFGFFLMPQVSYGASMELLSARETFPSFFRTVPSDRVQLTAAAELLQEFGWNWVAALGSDDEYGRQGLSIFSALAAARGICIAHEGLVPLPRANSPLLGKVQEVLHQVNQSSVQVVLLFASARAAHALFSYSISSKLSRKVWVASEAWLTSDLVMGLPGMAQVGTVLGFLQRGAQLHKFSQYVKTRLALAADPAFCAALGEREQGLEEDVVGRRCPQCDCITLQNVSAGLNHHQTFSVYAAVYSVAQALHNTLHCNASGCPVQEPVKPWQLLDNMYNLTFHAGGLTLRFNSNGNVDMEYDLKLWVWQGPVPELHNVGRFNGSLWIDSLKIRWHTSNNQKPVSQCSRQCQEGQVRRVKGFHSCCYDCVDCKAGSYRKSPDDLACTFCSQDEWSPERSTRCFRRRLRFLAWGEPAVLLLLLLFGLALGLVLAALGLFIRHRDSPLVQASGGPLACFGLVCLGLVCISVLLFPGQPSPARCLAQQPSSHLPLTGCLSTFILQAAEIFMESELPLSWADRLSGCLRGPWAWLVVLLAMLVEAALCAWYLVAFPPEVVTDWRMLPTEALVHCRARSWVSFGLVHATNATLAFLCFLGTFLVQSRPGRYNRARGLTFAMLAYFITWVSFVPLLANVQVVFRPAVQMGALLLCVLGILAAFHLPRCYLLVRQPELNTPEFFLGRGPGDARDRNDGHTGNQGKHE

>Blue monkey (*Cercopithecus mitis*) A0A0M4NXW0

MLRPAVLGLSLWALLHLGTGAPLCLSQQLRMKGDYVLGGLFPLGEAEEAGLGSRTRPSSPVCTRFSSNGLLWALAMKMAVEEINNRSDLLPGLRLGHDLFDTCSEPVVAMKPSLMFLAKADSRDIAAYCNYTQYQPRVLAVIGPHSSELAVVTGKFFGFFLMPQVSYGASMELLSARETFPSFFRTVPSDRVQLTAAAELLQEFGWNWVAALGSDDEYGRQGLSIFSALAAARGICIAHEGLVPLPRANSPLLGKVQEVLHQVNQSSVQVVLLFASVRAAHALFSYSISSKLSRKVWVASEAWLTSDLVMGLPGMAQVGTVLGFLQRGAQLHKFSQYVKTRLALAADPVFCAALGEREQGLEEDVVGRRCPQCDCITLQNVSAGLNHHQTFSVYAAVYSVAQALHNTLQCNASGCPMRDP

VKPWQLLENMYNLTFHAGGLMLRFNSNGNVDMEYDLKLWVWQGPVPELHDVGRFNGSLWVDSLKIRWHTSNNQMPVSQCSRQCQEGQVRRVKGFHSCCYDCVDCKAGSYRKSPDDLACTFCSQEEWSPERSTRCFRRRLRFLAWGEPAVLLLLLLLGLALGLVLAALGLFIRHRDSPLVQASGGPLACFGLVCLGLVCLSVLLFPGQPSPARCLAQQPSSHLPLTGCLSTLILQAAEIFVESELPLSWADRLSGCLRGPGAWLVVLLAMLVEAALCAWYLVAFPPEVVTDWRMLPTEALVHCRARSWVSFGLVHATNATLAFLCFLGTFLVQSRPGRYNRARGLTFAMLAYFITWVSFVPLLANVQVVLRPAVQMGALLLCVLGILAAFHLPRCYLLVRQPELNTPEFFLGRGPGDARDRNDGDTGNQGKHE

>Sykes' monkey (Cercopithecus albogularis) A0A0M3TY83

MLRPAVLGLSLWALLHLGTGAPLCLSQQLRMKGDYVLGGLFPLGEAEEAGLGSRTRPSSPVCTRFSSNGLLWALAMKMAVEEINNRSDLLPGLRLGHDLFDTCSEPVVAMKPSLMFLAKADSRDIAAYCNYTQYQPRVLAVIGPHSSELAVVTGKFFGFFLMPQVSYGASMELLSARETFPSFFRTVPSDRVQLTAAAELLQEFGWNWVAALGSDDEYGRQGLSIFSALAAARGICIAHEGLVPLPRANSPLLGKVQEVLHQVNQSSVQVVLLFASVRAAHALFSYSISSKLSRKVWVASEAWLTSDLVMGLPGMAQVGTVLGFLQRGAQLHKFSQYVKTRLALAADPVFCAALGEREQGLEEDVVGRRCPQCDCITLQNVSAGLNHHQTFSVYAAVYSVAQALHNTLQCNASGCPMRDPVKPWQLLENMYNLTFHAGGLMLRFNSNGNVDMEYDLKLWVWQGPVPELHDVGRFNGSLWVDSLKIRWHTSNNQMPVSQCSRQCQEGQVRRVKGFHSCCYDCVDCKAGSYRKSPDDLACTFCSQEEWSPERSTRCFRRRLRFLAWGEPAVLLLLLLLGLALGLVLAALGLFIRHRDSPLVQASGGPLACFGLVCLGLVCLSVLLFPGQPSPARCLAQQPSSHLPLTGCLSTLILQAAEIFVESELPLSWADRLSGCLRGPGAWLVVLLAMLVEAALCAWYLVAFPPEVVTDWRMLPTEALVHCRARSWVSFGLVHATNATLAFLCFLGTFLVQSRPGRYNRARGLTFAMLAYFITWVSFVPLLANVQVVLRPAVQMGALLLCVLGILAAFHLPRCYLLVRQPELNTPEFFLGRGPGDARDRNDGDTGNQGKHE

>Assam macaque (*Macaca assamensis*) A0A0M4PM80

MLCPAVLGLSLWALLHLGTGAPLCLSQQLRMKGDYVLGGLFPLGEAEEAGLGSRTRPSSPVCTRFSSNGLLWALAMKMAVEEINNRSDLLPGLRLGHDLFDTCSEPVVAMKPSLMFLAKADSRDIAAYCNYTQYQPRVLAVIGPHSSELAVVTGKFFGFFLMPQVSYGAGMELLSARETFPSFFRTVPSDRVQLVAAAELLQEFGWNWVAALGSDDEYGRQGLSTFSALAASRGICIAHEGLVPLPRANSPLLGKVQEVLHQVNQSSVQVVLLFASARAAHALFSYSISSKLSRKVWVASEAWLTSDLVMGLPGMAQVGTVLGFLQRGAQLHKFSQYVKTRLALAADPAFCAALGEREQGLEEDVVGRRCPQCDCITLQNVSAGLNHHQTFSVYAAVYSVAQALHNALQCSASGCPVQDPVKPWQLLENMYNLTFHAGGLTLRFNSNGNVDMEYDLKLWVWQGPVPELHDVGRFNGNLWIDSPKIRWHTSNNQKPVSQCSRQCQEGQVRRVKGFHSCCYDCVDCKAGSYRKSPDDLACTFCGQEEWSPERSTRCFRRRLRFLAWGEPAVLLLLLLFGLALGLVLAALGLFIRHRDSPLVQASGGPLACFGLVCLGLVCISVLLFPGQPSPARCLAQQPSSHLPLTGCLSTFILQAAEIFAESELPLSWADRLSGCLRGPWAWLVVLLAMLVEAALCAWYLVAFPPEVVTDWRMLPTEALVHCRTRSWVSFGLVHATNATLAFLCFLGTFLVQSRPGRYNRARGLTFAMLAYFITWVSFVPLLANVQVVLRPAVQMGALLLCVLGILAAFHLPRCYLLVQQPELNTPEFFLGRGPGDARDR

NDGDTGNQGKHE

>Stump-tailed macaque (*Macaca arctoides*) A0A0M5LP12

MLCPAVLGLSLWALLHLGTGAPLCLSQQLRMKGDYVLGGLFPLGEAEEAGLGSRTRPSSPVCTRFSSNGLLWALAMKMAVEEINNRSDLLPGLRLGHDLFDTCSEPVVAMKPSLMFLAKADSRDIAAYCNYTQYQPRVLAVIGPHSSELAVVTGKFFGFFLMPQVSYGAGMELLSARETFPSFFRTVPSDRVQLVAAAELLQEFGWNWVAALGSDDEYGRQGLSTFSALAASRGICIAHEGLVPLPRANSPLLGKVQEVLHQVNQSSVQVVLLFASARAAHALFSYSISSKLSRKVWVASEAWLTSDLVMGLPGMAQVGTVLGFLQRGAQLHKFSQYVKTRLALAADPAFCAALGEREQGLEEDVVGRRCPQCDCITLQNVSAGLNHHQTFSVYAAVYSVAQALHNALQCSASGCPVQDPVKPWQLLENMYNLTFHAGGLTLRFNSNGNVDMEYDLKLWVWQGPVPELHDVGRFNGSLWIDSPKIRWHTSNNQKPVSQCSRQCQEGQVRRVKGFHSCCYDCVDCKAGSYRKSPDDLACTFCGQEEWSPERSTRCFRRRLRFLAWGEPAVLLLLLLFGLALGLVLAALGLFIRHRDSPLVQASGGPLACFGLVCLGLVCISVLLFPGQPSPARCLAQQPSSHLPLTGCLSTFILQAAEIFAESELPLSWADRLSGCLRGPWAWLVVLLAMLVEAALCAWYLVAFPPEVVTDWRMLPTEALVHCRTRSWVSFGLVHATNATLAFLCFLGTFLVQSRPGRYNRARGLTFAMLAYFITWVSFVPLLANVQVVLRPAVQMGALLLCVLGILAAFHLPRCYLLVRQPELNTPEFFLGRGPGDARDRNDGDTGNQGKHE

>Japanese macaque (*Macaca fuscata*) A0A0M4PYM0

MLRPAVLGLSLWALLHLGTGAPLCLSQQLRMKGDYVLGGLFPLGEAEEAGLGSRTRPSSPVCTRFSSNGLLWALAMKMAVEEINNRSDLLPGLRLGHDLFDTCSEPVVAMKPSLMFLAKADSRDIAAYCNYTQYQPRVLAVIGPHSSELAVVTGKFFGFFLMPQVSYGAGMELLSARETFPSFFRTVPSDRVQLVAAAELLQEFGWNWVAALGSDDEYGRQGLSIFSALAASRGICIAHEGLVPLPRANSPLLGKVQEVLHQVNQSSVQVVLLFASARAAHALFSYSISSKLSRKVWVASEAWLTSDLVMGLPGMAQVGTVLGFLQRGAQLHKFSQYVKTRLALAADPAFCAALGEREQGLEEDVVGRRCPQCDCITLQNVSAGLNHHQTFSVYAAVYSVAQALHNALQCSASGCPVQDPVKPWQLLENMYNLTFHAGGLTLRFNSNGNVDMEYDLKLWVWQGPVPELHDVGRFNGSLWIDSPKIRWHTSNNQKPVSQCSRQCQEGQVRRVKGFHSCCYDCVDCKAGSYRKSPDDFACTFCGREEWSPERSTRCFRRRLRFLAWGEPAVLLLLLLFGLALGLVLAALGLFIRHRDSPLVQASGGPLACFGLVCLGLVCISVLLFPGQPSPARCLAQQPSSHLPLTGCLSTFILQAAEIFVESELPLSWADRLSGCLRGPWAWLVVLLAMLVEAALCAWYLVAFPPEVVTDWRMLPTEALVHCRTRSWVSFGLVHATNTTLAFLCFLGTFLVQSRPGRYNRARGLTFAMLAYFITWVSFVPLLANVQVVLRPAVQMGALLLCVLGILAAFHLPRCYLLVRQPELNTPEFFLGRGPGDARDRNDGDTGNQGKHE

>Crab-eating macaque (*Macaca fascicularis*) XP_005545155.1

MLCPAVLGLSLWALLHLGTGAPLCLSQQLRMKGDYVLGGLFPLGEAEEAGLGSRTRPSSPVCTRFSSNGLLWALAMKMAVEEINNRSDLLPGLRLGHDLFDTCSEPVVAMKPSLMFLAKADSRDIAAYCNYTQYQPRVLAVIGPHSSELAVVTGKFFGFFLMPQVSYGAGMELLSARETFPSFFRTVPSDRVQLVAAAELLQEFGWNWVAALGSDDEYGRQGLSTFSALAASRGICIAHEGLVPLPRANSPLLGKVQEVLHQVNQSSVQVVLLFASARAAHALFSYSISSKLSRKVWVASEAWLTSDLVMGLPGMAQVGTVLGFLQRGAQLHKFSQYVKTRLALAADPAFCAALGEREQGLEEDVVGRRCPQCDCITLQNVSAGLNHHQTFSVYAAVYSVAQALHNALQCSASGCPVQDPVKPWQLLENMYNLTFHAGGLTLRFNSNGNVDMEYDLKLWVWQGPVPELHDVGRFNGSLWIDSPKIRWHTSNNQKPVSQCSRQCQEGQVRRVKGFHSCCYDCVDCKAGSYRKSPDDLACTFCGQEEWSPERSTRCFRRRLRFLAWGEPAVLLLLLLFGLALGLVLAALGLFIRHRDSPLVQASGGPLACFGLVCLGLVCISVLLFPGQPSPARCLAQQPSSHLPLTGCLSTFILQAAEIFAESELPLSWADRLSGCLRGPWAWLVVLLAMLVEAALCAWYLVAFPPEVVTDWRMLPTEALVHCRTRSWVSFGLVHATNATLAFLCFLGTFLVQSRPGRYNRARGLTFAMLAYFITWVSFVPLLANVQVVLRPAVQMGALLLCVLGILAAFHLPRCYLLVRQPELNTPEFFLGRGPGDARDRNDGDTGNQGKHE

>Pig-tailed macaque (*Macaca nemestrina*) A0A2K6DXL7

MLCPAVLGVSLWALLHLGTGAPLCLSQQLRMKGDYVLGGLFPLGEAEEAGLGSRTRPSSPVCTRFSSNGLLWALAMKMAVEEINNRSDLLPGLRLGHDLFDTCSEPVVAMKPSLMFLAKADSRDIAAYCNYTQYQPRVLAVIGPHSSELAVVTGKFFGFFLMPQVSYGAGMELLSARETFPSFFRTVPSDRVQLVAAAELLQEFGWNWVAALGSDDEYGRQGLSTFSALAASRGICIAHEGLVPLPRANSPLLGKVQEVLHQVNQSSVQVVLLFASARAAHALFSYSISSKLSRKVWVASEAWLTSDLVMGLPGMAQVGTVLGFLQRGAQLHKFSQYVKTRLALAADPAFCAALGEREQGLEEDVVGRRCPQCDCITLQNVSAGLNHHQTFSVYAAVYSVAQALHNALRCSASGCPVQDPVKPWQLLENMYNLTFHAGGLTLRFNSNGNVDMEYDLKLWVWQGPVPELHDVGRFNGSLWIDSPKIRWHTSNNQKPVSQCSRQCQEGQVRRVKGFHSCCYDCVDCKAGSYRKSPDDLACTFCGREEWSPERSTRCFRRRLRFLAWGEPAVLLLLLLFGLALGLVLAALGLFIRHRDSPLVQASGGPLACFGLVCLGLVCISVLLFPGQPSPARCLAQQPSSHLPLTGCLSTFILQAAEIFAESELPLSWADRLSGCLRGPWAWLVVLLAMLVEAALCAWYLVAFPPEVVTDWRMLPTEALAHCRTRSWVSFGLVHATNATLAFLCFLGTFLVQSRPGRYNRARGLTFAMLAYFITWVSFVPLLANVQVVLRPAVQMGALLLCVLGILAAFHLPRCYLLVRQPELNTPEFFLGRGPGDARDRNDGDTGNQGKHE

>Rhesus macaque (Macaca mulatta) A0A5F8AJP6

MLCPAVLGLSLWALLHLGTGAPLCLSQQLRMKGDYVLGGLFPLGEAEEAGLGSRTRPSSPVCTRFSSNGLLWALAMKMAVEEINNRSDLLAGLRLGHDLFDTCSEPVVAMKPSLMFLAKADSRDIAAYCNYTQYQPRVLAVIGPHSSELAVVTGKFFGFFLMPQVSYGAGMELLSARETFPSFFRTVPSDRVQLVAAAELLQEFGWNWVAALGSDDEYGRQGLSIFSALAASRGICIAHEGLVPLPRANSPLLGKVQEVLHQVNQSSVQVVLLFASARAAHALFSYSISSKLSRKVWVASEAWLTSDLVMGLPGMAQVGTVLGFLQRGAQLRKFSQYVKTRLALAADPAFCAALGEREQGLEEDVVGRRCPQCDCITLQNVSAGLNHHQTFSVYAAVYSVAQALHNALQCSASGCPVQDPVKPWQLLENMYNLTFHAGGLTLRFNSNGNVDMEYDLKLWVWQGPVPELHDVGRFNGSLWIDSPKIRWHTSNNQKPVSQCSRQCQEGQVRRVKGFHSCCYDCVDCKAGSYRKSPDDLACTFCGREEWSPERSTRCFRRRLRFLAWGEPAVLLLLLLFGLALGLVLAALGLFIRHRDSPLVQASGGPLACFGLVCLGLVCISVLLFPGQPSPARCLAQQPSSHLPLTGCLSTFILQAAEIFVESELPLSWADRLSGCLRGPWAWLVVLLAMLVEAALCAWYLVAFPPEVVTDWRMLPTEALVHCRTRSWVSFGLVHATNATLAFLCFLGTFLVQSRPGRYNRARGLTFAMLAYFITWVSFVPLLANVQVVLRPAVQMGALLLCVLGILAAFHLPRCYLLVRQPELNTPEFFLGRGPGDARDRNDGDTGNQGKHE

>Gelada baboon (*Theropithecus gelada*) ALE59895

MLRPAVLGLSLWALLHLGTGAPLCLSQQLRMKGDYVLGGLFPLGEAEEAGLSSRTRPSSPVCTRLSSNGLLWALAMKMAVEEINNRSDLLPGLRLGHDLFDTCSEPVVAMKPSLMFLAKADSRNIAAYCNYTQYQPRVLAVIGPHSSELAVVTGKFFGFFLMPQVSYGASMELLSARETFPSFFRTVPSDRVQLTAAAELLQEFGWNWVAALGSDDEYGRQGLSIFSALAAARGICIAHEGLVPLPRANSPLLGKVQEVLHQVNQSSVQVVLLFASPRAAHALFSYSISSKLSRKVWVASEAWLTSDLVMGLPGMAQVGTVLGFLQRGAQLHKFSQYVKTRLALAADPVFCAALGEREQGLEEDVVGRRCPQCDCITLQNVSAGLNHHQTFSVYAAVYSVAQALHNTLQCNASGCPVQDPVKPWQLLDNMYNLTFHAGGLTLRFNSNGNVDMEYDLKLWVWQGPVPELHDVGRFNGSLWIDSLKIRWHTSNNQKPVSQCSRQCQEGQVRRVKGFHSCCYDCVDCKAGSYRKSPDDLACTFCSQDEWSPERSTRCFRRRLRFLAWGEPAVLLLLLLFGLALGLVLAALGLFIRHRDSPLVQASGGLLACFSLVCLGLVCISVLLFPGQPSPARCLAQQPSSHLPLTGCLSTFILQAAEIFVESELPLSWADRLSGCLRGPWAWLVVLVAMLVEAALCAWYLVAFPPEMVTDWRMLPTEALVHCRARSWVSFGLVHATNATLAFLCFLGTFLVQSRPGRYNRARGLTFAMLAYFITWVSFVPLLANVQVVLRPAVQMGALLLCVLGILAAFHLPRCYLLVRQPELNTPEFFLGRGPGDARDRNDGDTGNQGKHE

>Hamadryas baboon (*Papio hamadryas*) A2T0N3

MLRPAVLGLSLWALLHLGTGAPLCLSQQLRMKGDYVLGGLFPLGEAEEAGLRSRTRPSSPVCTRFSSNGLLWALAMKVAVEEINNKSDLLPGLRLGYDLFDTCSEPVVAMKPSLMFLAKAGSRDIAAYCNYTQYQPRVLAVIGPHSSELAMVTGKFFSFFLMPQVSYGASMELLSARETFPSFFRTVPSDRVQLTAAAELLQEFGWNWVAALGSDDEYGRQGLSIFSALAAARGICIAHEGLVPLPRANSPLLGKVQEVLHQVNQSSVQVVLLFASARAAHALFSYSISSKLSRKVWVASEAWLTSDLVMGLPGMAQVGTVLGFLQRGAQLHKFSQYVKTRLALAADPVFCATLGEREQGLEEDVVGRRCPQCDCITLQNVSAGLNHHQTFSVYAAVYSVAQALHNTLQCNASGCPVQDPVKPWQLLDNMYNLTFHAGGLTPRFNSSGNVDMEYDLKLWVWQGSVPRLHDVGRFNGSLRTERLKIRWHTSDNQKPVSRCSRRCQEGQVRRVKGFHSCCYDCVDCEAGSYRQNPDDIACPFCGQDEWSPERSTRCFRRRSRFLAWGEPAVLLLLLLLSLALGLVLAALGLFVHHRDSPLVQASGGPLACFGLVCLGLVCLSVLLFPGQPSPARCLAQQPLSHLPLTGCLSTLFLQAAEIFVESELPLSWADRLSGCLRGPWAWLVVLLAMLVEVALCTWYLVAFPPEVVTDWHMLPTEALVHCRTRSWVSFGLVHATNATLAFLCFLGTFLVQSRPGRYNRARGLTFAMLAYFITWVSFVPLLANVQVVLRPAVQMGALLLCVLGILAAFHLPRCYLLVRQPELNTPEFFLGRGPGDARDRNDGDTGNQGKHE

>Mandrill (*Mandrillus sphinx*) A0A0M4NTT1

MLRPAVLGLSLWAVLHLGTGAPLCLSQQLRMKGDYVLGGLFPLGEAEEAGLGSRTRPSSPVCTRFSSNGLLWALAMKMAVEEINNRSDLLPGLRLGHDLFDTCSEPVVAMKPSLMFLAKADSRDIAAYCNYTQYQPRVLAVIGPHSSELAVVTGKFFGFFLMPQVSYGASMELLSARETFPSFFRTVPSDRVQLTAAAELLQEFGWNWVAALGSDDEYGRQGLSIFSALAAARGICIAHEGLVPLPRANSPLLGKVQEVLHQVNQSSVQVVLLFASARAAHALFSYSISSKLSRKVWVASEAWLTSDLVMGLPGMAQVGTVLGFLQRGAQLHKFSQYVKTRLALAADPAFCAALGEREQGLEEDVVGRRCPQCDCITLQNVSAGLNHHQTFSVYAAVYSVAQALHNTLHCNASGCPVQEPVKPWQLLDNMYNLTFHAGGLTLRFNSNGNVDMEYDLKLWVWQGPVPELHDVGRFNGSLWIDSLKIRWHTSNNQKPVSQCSRQCQEGQVRRVKGFHSCCYDCVDCKAGSYRKSPDDLACTFCSQDEWSPERSTRCFRRRLRFLAWGEPAVLLLLLLFGLALGLVLAALGLFIRHRDSPLVQASGGPLACFGLVCLGLVCISVLLFPGQPSPARCLAQQPSSHLPLTGCLSTFILQAAEIFVESELPLSWADRLSGCLRGPWAWLVVLLAMLVEAALCAWYLVAFPPEVVTDWRMLPTEALVHCRARSWVSFGLVHATNATLAFLCFLGTFLVQSRPGRYNRARGLTFAMLAYFITWVSFVPLLANVQVVFRPAVQMGALLLCVLGILAAFHLPRCYLLVRQPELNTPEFFLGRGPGDARDRNDGHTGNQGKHE

>Drill (*Mandrillus leucophaeus*) XP_011857372

MLRPAVLGLSLWAVLHLGTGAPLCLSQQLRMKGDYVLGGLFPLGEAEEAGLGSRTRPSSPVCTRFSSNGLLWALAMKMAVEEINNRSDLLPGLRLGHDLFDTCSEPVVAMKPSLMFLAKADSRDIAAYCNYTQYQPRVLAVIGPHSSELAVVTGKFFGFFLMPQVSYGASMELLSARETFPSFFRTVPSDRVQLTAAAELLQEFGWNWVAALGSDDEYGRQGLSIFSALAAARGICIAHEGLVPLPRANSPLLGKVQEVLHQVNQSSVQVVLLFASARAAHALFSYSISSKLSRKVWVASEAWLTSDLVMGLPGMAQVGTVLGFLQRGAQLHKFSQYVKTRLALAADPAFCAALGEREQGLEEDVVGRRCPQCDCITLQNVSAGLNHHQTFSVYAAVYSVAQALHNTLHCNASGCPVQEPVKPWQLLDNMYNLTFHAGGLTLRFNSNGNVDMEYDLKLWVWQGPVPELHDVGRFNGSLWIDSLKIRWHTSNNQKPVSQCSRQCQEGQVRRVKGFHSCCYDCVDCKAGSYRKSPDDLACTFCSQDEWSPERSTRCFRRRLRFLAWGEPAVLLLLLLFGLALGLVLAALGLFIRHRDSPLVQASGGPLACFGLVCLGLVCISVLLFPGQPSPARCLAQQPSSHLPLTGCLSTFILQAAEIFVESELPLSWADRLSGCLRGPWAWLVVLLAMLVEAALCAWYLVAFPPEVVTDWRMLPTEALVHCRARSWVSFGLVHATNATLAFLCFLGTFLVQSRPGRYNRARGLTFAMLAYFITWVSFVPLLANAQVVFRPAVQMGALLLCVLGILAAFHLPRCYLLVRQPELNTPEFFLGRGPGDARDRNDGHTGNQGKHE

>Red guenon (*Erythrocebus patas*) A0A0M4P9G6

MLRPAVLGLSLWALLHLGTGAPLCLSQQLRMKGDYVLGGLFPLGEAEEVGLGSRTRPSSPVCTRFSSNGLLWALAMKMAVEEINNRSDLLPGLRLGHDLFDTCSEPVVAMKPSLMFLAKADSRNIAAYCNYTQYQPRVLAVIGPHSSELAVVTGKFFGFFLMPQVSYGASMELLSARETFPSFFRTVPSDRVQLTAAAELLQEFGWNWVAALGSDDEYGRQGLSIFSALAAARGICIAHEGLVPLPRANSLLLGKVQEVLHQVNQSSVQVVLLFASARAAHALFSYSISSKLSRKVWVASEAWLTSDLVMGLPGMAQVGTVLGFLQRGAQLHKFSQYVKTRLALAADPVFCAALGEREQGLEEDVVGRRCPQCDCITLQNVSAGLNHHQTFSVYAAVYSVAQALHSTLQCNASGCPMRDPVKPWQLLENMYNLTFHAGGLTLRFNSNGNVDMEYDLKLWVWQGPVPELHDVGRFNGSLWIDSLKIHWHTSNNQKPVSQCSRQCQEGQVRRVKGFHSCCYDCVDCKAGSYRKSPDDLACTFCSQDEWSPERSTQCFRRRLRFLAWGEPAVLLLLLLLSLALGLVLAALGLFIRHRDSPLVQASGGPLACFGLVCLGLVCISVLLFPGQPSPARCLAQQPSSHLPLTGCLSTLILQAAEIFVESELPLSWADRLSGCLRGPWAWLVVLLAMLVEAALCTWYLMAFPPEVVTDWRMLPTEALVHCRARSWVSFGLVHATNATLAFLCFLGTFLVQSRPGRYNRARGLTFAMLAYFITWVSFVPLLANVQVVLRPAVQMGALLLCVLGILAAFHLPRCYLLVRQPELNTPEFFLGRGPGDARDRNDGDTGNQGNHE

>Ma's night monkey (*Aotus nancymaae*) XP_012323332

MLGSAVLGLSLWALLHLRTGAPLCLSRQLKMKGDYMLGGLFPLGEAGEAALHSRTRPTSLVCTRFSWNGLLWALTMKMAVEEINNRSDLLPGLRLGYDLFDTCSESVVTMKPSLMFLAKANSHDIAAYCNYTQYQPRVLAVIGPHSSELALVTGKFFGFFLMPQVSYGASMDLLSTRETFPSFFRTVPSDRVQLTAAVELLQRLGWNWVAALGSDDEYGRQGLSIFSGLAAARGICIAHEGLVPLPRADSPWAGKVQELLPQVNQSGVQVVLLFASARAAHAFFSYSISRRLLPKVWVASEAWLTSDLVMGLPGMAEVGTVLGFLQRGAPLPKFSQYVKTHLALAADPAFCASLGESEQGLEEHVVGPRCPQCDNVTLQNVSAGLPHHRTFSVYAAVYGVAQALHNTLRCSASGCPTQDPVKPWQLLHNMYNMTFRAAGLALRFDGSGNVDAEYDLKLWVWRGPVPELHNVGVFNGSLWPERLKMRWHTPGNQEPVSQCSRQCQEGQVRRVKGFHSCCYDCVDCEAGSYRRNPDDPACTPCRHDQWSPERSTRCFRRRPRFLTWGEPAVLLLLLLLGLALGLVLAALGLFVRHRDSPLVQASGGALACFGLVCLGLVCLSVLLFPGQPSPARCLAQQPLSQLPLTGCLSTLFLQAAETFVESELPQSWADRLRGCLRGPRAWLAVLLAMLVEAALCAWYLLAFPPEVVTDWRVLPTEALVHCRTRSWVSFGLVHATNAILAFLCFLGTFLVQSQPGRYNRARGLTFATLAYFITWVSFVPLLANVQVALRPAMQMGAFLLCTLGILAAFHLPRCYLLLWQPGLNTPEFFLGGARMPKAGMAVGTEEAQGKNE

>White-tufted-ear marmoset (*Callithrix jacchus*) JAB38243

MLGSAVLGLSLWALLHLRTGAPLCLSRQLKMKGDYMLGGLFPLGEAGEAAFHSRTRPSSLVCTRFSWNGLLWALAMKMAVEEINNQSDLLPGLRLGYDLFDTCSESVVTMKPSLMFLAKVNSHDIAAYCNYTQYQPRVLAVIGPHSSELALVTGKFFGFFLMPQVSYGASMDLLSTRETFPSFFRTVPSDRVQLMATVELLQQLGWNWVAALGSDDEYGRQGLSIFSGLAAARGICIAHEGLVPLPRADGLWVGKVQELLPQVNQSSIQVVLLFASAHAAHAFFRHSINRRLLPKVWVASEAWLTSDLVMGLPGMAEVGTVLGFLQRGAQLPKFPQYVKTHLALAADPAFCASLSEREQGLEEHVVGPRCPQCDDVTLQNMSTRLQHHQTFSVYAAVYSVAQALHNTLRCNASGCPKQDPVKPWQLLQNMYNMTFHAAGLALRFDSSGNVDVEYDLKLWVWRGPVPELHDVGVFNGSLWPERLKMRWHTPDNQEPVSQCSRQCQEGQVRRVKGFHSCCYDCVDCEAGSYRRNPDDPTCTPCRHDQWSPERSTRCFRRRPRFLTWSEPAVLLLLLLLGLALGLVLAALGLFIHHRDSPLVQASGGGLACFGLVCLGLVCLSVLLFPGQPSPARCLAQQPLSHLPLTGCLSTLFLQAAETFVESELPQSWADRVRGCLRGSRAWLAVLLAMLVEAALCAWYLLAFPPEVVTDWRVLPTEALVHCCTRSWVSFGLVHATNAILAFLSFLGTFLVQSQPGRYNRARGLTFAMLAYFITWVSFVPLLANVQVALRPAMQMGAFLLCTLGILAAFHLPRCYLLLWQPGLNTPEFFLGGARMPNAGMAVGTEEAQGKIE

>Panamanian white-faced capuchin (*Cebus imitator*) XP_017365973

MVGSAVLGLGLWALLHLRTGAPLCLSQQLKMKGDYVLGGLFPLGEAGETALHSRTQPNSLVCTRFSWNGLLWALAMKMAVEEINNRSDLLPGLRLGYDVFDTCSEPTVTMKPSLMFLAKANSHDIAAYCNYTQYQPRVLAVIGPHSSELALVTGKFFGFFLMPQVSYGASMDLLSTRETFPSFFRTVPSDRVQLVATVELLQQLGWNWVAALGSDDEYGRQGLSIFSGLAAARGICIAHEGLVPLPHADSPWVSKVQELLPQVNQSSIQVVLLFASARAAQAFFSYGISRRLSPKVWVASEAWLTSDLVMGLPGMTEVGTVLGFLQRGAQLPKFSQYVKTHLALAADPAFCASLGEREQGLEEHVVGPRCPQCDDVTLQNVSARLQHHQTFSVYAAVYSVAQGLHNTLRCNASGCPVQDPVKPWQLLQNMYNMTFRVAGLVLRFDSSGNVDMEYDLKLWVWRGPVPELHNVGIFNGSLWPERLKMRWHTPDNQEPVSQCSRQCQEGQVRRVKGFHSCCYDCVDCEAGSYRRNPDDPTCTPCRHDQWSPERSTRCFRRRPRFLTWGEPAVLLLLLLLGLALGLVLAALGLFIRHRDSPLVQASGGALACFGLVCLGLVCLSVLLFPGQPSPARCLVQQPLSHLPLTGCLSTLFLQAAETFVESELPQSWADRLRGCLQGPWAWLAVLLAMLVEAALCAWYLLAFPPEVVTDWRVLPTEALVHCRTRSWVSFGLVHATNAILAFLCFLGTFLVQSQPGRYNRARGLTFAMLAYFIIWVSFVPLLANVQVALRPAMQMGAFLLCTLGILTAFHLPRCYLLLWQPGLNTPEFFLGGAQMPKVGMVVETEEAQGKK

>Brown-capped capuchin (*Sapajus apella*) XP_032117956

MLGSAVLGLGLWALLHLRTGAPLCLSQQLKMKGDYVLGGLFPLGEAGETALHSRTRPNSLVCTRFSWNGLLWALAMKMAVEEINNRSDLLPGLRLGYDLFDTCSEPTVTMKPSLMFLAKANSHDIAAYCNYTQYQPRVLAVIGPHSSELALVTGKFFGFFLMPQVSYGASMDLLSTRETFPSFFRTVPSDRVQLVATVELLQQLGWNWVAALGSDDEYGRQGLSIFSGLAAARGICIAHEGLVPLPHADSPWVSKVQELLPQVNQSSIQVVLLFASVRAAQAFFSYGISRRLSPKVWVASEAWLTSDLVMGLPGMAEVGTVLGFLQRGAQLPKFSQYVKTHLALAADPAFCASLGEREQGLEEHVVGPRCPQCDDVTLQNVSAKLQHHQTFSVYAAVYSVAQGLHNTLRCNASGCPMQDPVKPWQLLQNMYNMTFRVAGLVLRFDSSGNVDMEYDLKLWVWRGPVPELHDVGIFNGSLWPERLKMRWHTPDNQEPVSQCSRQCQEGQVRRVKGFHSCCYDCVDCEAGSYRRNPDDPTCTPCRHDQWSPERSTRCFRRRPRFLTWGEPAVLLLLLLLGLALGLVLAALGLFIRHRDSPLVQASGGALACFGLVCLGLVCLSVLLFPGQPSPARCLAQQPLSHLPLTGCLSTLFLQAAETFVESELPQSWADRLRGCLQGPWAWLVVLLAMLVEAALCAWYLLAFPPEVVTDWRVLPTEALVHCRTRSWVSFGLVHATNAILAFLCFLGTFLVQSQPGRYNRARGLTFAMLAYFIIWVSFVPLLANVQVALRPAMQMGAFLLCTLGILTAFHLPRCYLLLWQPGLNTPEFFLGGAQMPKVGMVVETEEAQGKK

>Bolivian squirrel monkey (*Saimiri boliviensis boliviensis*) XP_003939708

MLGSGVLGLSLWTLLHLRTGAPSCLSRQLKMKGDYVLGGLFPLGEAGEAALHSRTRPSSLVCTRFSWNGLLWALAMKMAVEEINNRLDLLPGLRLGYDLFDTCSEPTVTMKPSLMFLAKANSHDIAAYCNYTQYQPRVLAVIGPHSSELALVTGKFFGFFLMPQVSYGASMDLLSTRETFPSFFRTVPSDRVQLVATVELLQQLGWNWVAALGSDDEYGRQGLSIFSGLAAARGICIAHEGLVPLPRANSPWVGKVQELLPQLNQTSIQVVLLFASARAAHTFFSHIISRRLSPKVWVASEAWLTSDLVMGLPGMAEVGTVLGFLQRGAQLPEFSQYVKTHLALAADPAFCTSLGEREQGLEEHVVGPRCPQCDDITLQNVPARLQHHQTFSVYAAVYSVAQALHNTLGCNASGCPMQDPVKPWQVLQNMYNMTFHAAGQVLRFDSSGNVDVEYDLKLWVWRGPVPELHNVGIFNGSLWPERLKMRWHTPDNQEPVSQCSRQCQEGQVRRVKGFHSCCYDCVDCEAGSYRRNPDDPTCTPCRHDQWSPKRSTRCFHRRPRFLTWGEPAVLLLLLLLGLALGLVLATLGLFIRHRDSPLVQASGGALACFGLVCLGLVCLSVLLFPGQPSPARCLAQQPLSHLPLTGCLSTLFLQAAETFVESELPPSWADRLWGCLRGPRAWLAVLLAMLVEAALCAWYLLAFPPEVVTDWRVLPTEALVHCRTRSWVSFGLVHTTNAILAFLCFLGTFLVQSQPGRYNRARGLTFAMLAYFITWVSFVPLLANVEVALRPAVQMGAFLLCTLGILAAFHLPRCYLLLWQPGLNTPEFFLGGAQIPKVGMVVGTEEAQGKNE

>Squirrel monkey (*Saimiri sciureus*) ABD14701

MLGSGVLGLSLWTLLHLRTGAPSCLSRQLKMKGDYVLGGLFPLGEAGEAALHSRTRPSSLVCTRFSWNGLLWALAMKMAVEEINNRLDLLPGLRLGYDLFDTCSEPTVTMKPSLMFLAKANSHDIAAYCNYTQYQPRVLAVIGPHSSELALVTGKFFGFFLMPQVSYGASMDLLSTRETFPSFFRTVPSDRVQLVATVELLQQLGWNWVAALGSDDEYGRQGLSIFSGLAAARGICIAHEGLVPLPRANSPWVGKVQELLPQLNQTSIQVVLLFASARAAHTFFSHIISRRLSPKVWVASEAWLTSDLVMGLPGMAEVGTVLGFLQKGAQLPEFSQYVKTHLALAADPAFCTSLGEREQGLEEHVVGPRCPQCDDITLQNVPARLQHHQTFSVYAAVYSVAQALHNTLGCNASGCPMQDPVKPWQVLQNMYNMTFHAAGQVLRFDSSGNVDVEYDLKLWVWRGPVPELHNVGIFNGSLWPERLKMRWHTPDNQEPVSQCSRQCQEGQVRRVKGFHSCCYDCVDCEAGSYRRNPDDPTCTPCRHDQWSPKRSTRCFHRRPRFLTWGEPAVLLLLLLLGLALGLVLATLGLFIRHRDSPLVQASGGALACFGLVCLGLVCLSVLLFPGQPSPARCLAQQPLSHLPLTGCLSTLFLQAAETFVESELPPSWADRLWGCLRGPRAWLAVLLAMLVEAALCAWYLLTFPPEVVTDWRVLPTEALVHCRTRSWVSFGLVHTTNAILAFLCFLGTFLVQSQPGRYNRARGLTFAMLAYFITWVSFVPLLANVEVALRPAVQMGAFLLCTLGILAAFHLPRCYLLLWQPGLNTPEFFLGGAQIPKVGM

VVGTEEAQGKNE

>Coquerel's sifaka (*Propithecus coquereli*) XP_012513011

MPSLAVLGLSLAALLSPGTGAPLCLSQQLKMSGDYMLGGLFPLGTAEEASLRGRMQPSSPVCTRFSAPGLLWALTVKMAVEEINNESTLLPGLRLGYDLFDTCSEPVVAMKPSLMFLAEIGSRDIAAYCDYTRYRPRVLAVIGPHSSELALVTGKFFSFFLMPQVSYGASMDRLSTRETFPSFFRTVPSDRVQLTAMVELLQEFGWNWVAALGSDDEYGRQGLSIFSSLANAQGICIAHEGLVPLPHADGPQLGKVQDVLHQVNQSNVQVVVLFASVHAAYNLFSYSIRYKLSPKVWVASEAWLTSNQVMALPGMAQVGTVLGFLHRGTPLPEFSNYVQTCLALAADPAFCASLSMEQPDLEEHVVGPRCPQCDHITLQNVSAELRNHRAFAAYAAVYSVAQALHNTLQCNSSGCPAREPVQPWELLENMYNMSFHVRGLALQFDINGNVDMEHDLKLWVWGQRTPTLLTVGTFHKHLQLQHGKISWHTAHNQTPVSQCSRQCREGQVRRVKGFHSCCYDCVDCKAGSYQHSADDLTCTPCNQDEWSPERSTRCFRRTTKFLAWGEPAVLLLLLLLGLVLGLVLAALGLFVHHLDSPLVQASGGLLACFGLACLGLVCLGSLLFPGRPSPASCLAQQPLSHLPLTGCLSTLFLQAAKTFVESELPPSWADWLRGRLQGPWAWLAVLLAVLVEAALCTWYLTAFPPEVVTDWQVLPTEALVHCRMHSWVSLGLVHATNATLASLCFLGTFLVQSRPRRYSHARGITFAMLAYLIIWVSFVPLLANVQVAYQPAVQMGALLLCALGILAACHLPKCYLLLWQPELNTPEFFLGGGPGDATGR

DSGQGEEGTRSKNK

>Gray mouse lemur (*Microcebus murinus*) XP_012631854

MPSLAVLGLSLAALLSPGTGDPLCLSRQLKMSGDYVLGGLFPLGTAEDASLRGRTQPRGPVCSRFSALGLLWALTVTMAVEEINNGSALLPGLRLGYDLFDTCSEPVVAMKPSLMFLARTGSCDIAAYCDYTRYRPRVLAVIGPHSSELALVTGKFFSFFLVPQVSYGASMDRLSTRETFPSFFRTVPSDRVQLTATVELLQEFGWNWVAALGSDDEYGRQGLSIFSGLANAQGICIAHEGLVPLSHTDRQQLGKVQDILHQVNQSNVQVVVLFASAHAASALFTYSIRYKLSPKVWVASEAWLTSERVMALPGMAQVGTVLGFLHRSLPLPKFANYVKTRLHLAANPAFCASLSAEQPGLEEHVVGPRCPQCDCISLQNVSAELANQRTFPAYAAVYSVAQALHNTLQCNSSGCPAHEPVQPWQLLENMYNMSFRAHGLTLQFDVNGNVDMEYDLKLWEWKEPTPMLRTVGTFHRRLHLLQHSQIVWHTAHNEVPVSQCSRQCREGQVRRVKGFHSCCYDCVDCKAGSYQHRADDFICTTCNHDEWSPDRSTRCFPRTTRFLAWGDPAVLGLLLLLALVLGLVLAALGLFVHHQDSPLVQASGGLLACFGLACLGLVCLGSLLFPGRPSPASCLAQQPLSHLPLTGCLSTLFLQAAEIFVESELPPSWAAWLRGHLQGPWAWLVVLLAILVEAALCAWYLTAFPPEVVTDWQVLPTEALVHCRVHSWVGLGLVHAANAALASLCFLGTFLVQSRPSRYSRARGITFATLAYLITWVSFVPLLANVQVAYQPAVQMGALLLCALGILAAVHLPKCYLLLWQPELNTPEFFLGGAPGEATGRDGSRGEEGTRGKSE

>Ring-tailed Lemur (*Lemur catta*) XP_045404737

MPSFAVLGLSLAAFLSPGMGAPLCLSRQLKMSGDYVLGGLVPLGTAEETSLRGRTQPSSPVCTRFSAVGLLWALTVKMAVEEINNGSALLPGLHLGYDLFDTCSEPVVAMKPSLMFLARVGSCDIAAYCDYTRYRPRVLAVIGPHSSELALVTGKFFSFFLVPQVSYGASMDRLSTRETFPSFFRTVPSDRVQLTAMVELLQEFGWNWVAALGSDDEYGRQGLSIFSGLANAQGICIAHEGLVPLSQADGLQLGKVQDVLHQVNQSNVQVVVLFATAHAAYALFTYSISCKLSPKVWVASEPWLTSERVMALAGMAQVGTVLGFMQRGLLLPEFSDYVKTRLALAADPAFCASLSVEQPDLEEHVVGPRCPQCDHITLQNVSAELMYHRTFPAYAAVYSVAQALHNTLQCNSSGCPAREPVQPWQLLENMYNMSFRARSLELQFDVNGNVDMEFDLKLWVWEQQTPKLLTVGTFHRHLQVQHGKILWHTAHNQTPVSQCSRQCREGQVRRVKGFHSCCYDCVDCRAGSYQSSTDDLTCTLCNRDEWSPERSTRCFPRRTRFLAWGDPAVLLLLLLLGLVLGLVLAALGLFVHHQDSPLVQASGGLLACFGLACLGLVCLSSLLFPGRPSPTSCLAQQPLAHLPLTGCLSTLLLQAAEIFVESELPQSWADWLRGRLQGPWAWLAVLLPILVEAALCTWYLTAFPPAVVTDWQVLPTEALVHCRVHSWVSLGLVHATNATLASLCFLGTFLVQSRPSRYSRARGITFATLAYLITWVSFVPLLANVQVAYQPAVQMGALLLSALGILAAFHLPKCYLLLWQPELNTPEFFLGGGPGDATGRDGNRAEEGTRGKSE
